# Pangenome analysis of *Corynebacterium striatum*: insights into a neglected multidrug-resistant pathogen

**DOI:** 10.1186/s12866-023-02996-6

**Published:** 2023-09-08

**Authors:** Wedad M. Nageeb, Helal F. Hetta

**Affiliations:** 1 Department of Medical Microbiology and Immunology, Faculty of Medicine, Suez Canal University, Ismailia, 41111 Egypt; 2 Department of Medical Microbiology and Immunology, Faculty of Medicine, Assiut University, Assiut, 71515 Egypt

**Keywords:** *Corynebacterium striatum*, Pangenome analysis, *Corynebacterium antimicrobial resistance*, *Corynebacterium virulence*, Neglected pathogens, Prophage elements, Integrons

## Abstract

**Background:**

Over the past two decades, *Corynebacterium striatum* has been increasingly isolated from clinical cultures with most isolates showing increased antimicrobial resistance (AMR) to last resort agents. Advances in the field of pan genomics would facilitate the understanding of the clinical significance of such bacterial species previously thought to be among commensals paving the way for identifying new drug targets and control strategies.

**Methods:**

We constructed a pan-genome using 310 genome sequences of *C. striatum*. Pan-genome analysis was performed using three tools including Roary, PIRATE, and PEPPAN. AMR genes and virulence factors have been studied in relation to core genome phylogeny. Genomic Islands (GIs), Integrons, and Prophage regions have been explored in detail.

**Results:**

The pan-genome ranges between a total of 5253–5857 genes with 2070 − 1899 core gene clusters. Some antimicrobial resistance genes have been identified in the core genome portion, but most of them were located in the dispensable genome. In addition, some well-known virulence factors described in pathogenic *Corynebacterium* species were located in the dispensable genome. A total of 115 phage species have been identified with only 44 intact prophage regions.

**Conclusion:**

This study presents a detailed comparative pangenome report of *C. striatum.* The species show a very slowly growing pangenome with relatively high number of genes in the core genome contributing to lower genomic variation. Prophage elements carrying AMR and virulence elements appear to be infrequent in the species. GIs appear to offer a prominent role in mobilizing antibiotic resistance genes in the species and integrons occur at a frequency of 50% in the species. Control strategies should be directed against virulence and resistance determinants carried on the core genome and those frequently occurring in the accessory genome.

**Supplementary Information:**

The online version contains supplementary material available at 10.1186/s12866-023-02996-6.

## Background

Recent developments in sequencing technologies have aided in providing an in-depth understanding of bacterial species characteristics and behavior through studying its pan-genome. Tettelin et al. [1] was the first to use the term pangenome to describe the entire gene repertoire of a species by analyzing a number of individual members. A large revolution has then followed in the field of bacterial pangenome studies where many important species, genera or phyla have been comparatively analyzed using this approach [2, 3]. Defining the structure of the pangenome helps to understand the composition of its genomic repertoire and to select for therapeutic targets [4].

Non-diphtherial *Corynebacterium* species including *Corynebacterium striatum* are known as part of the human microbiota living as commensals on skin, mucus membranes and also in the respiratory tract [5]. However, their pathogenic potential has been growing recently being identified as the etiologic agent of different types of invasive infections [6]. Over the past 5 decades, reports from different countries have identified the organism as a causative agent in cases of bacteremia, septic arthritis, endocarditis, meningitis, osteomyelitis, sinusitis, and pulmonary infection [7]. Additionally, it has been incriminated in cases of keratitis, liver abscesses, peritonitis, skin wounds, and surgical infections [8].

Despite its significance, *C. striatum* is still considered a neglected pathogen and is commonly overlooked in routine microbiologic diagnosis. It is usually treated as a part of mixed infection if not overlooked. In these situations, empiric therapy is used facilitating the selection for multi-resistant strains or encouraging the horizontal transfer of interspecies resistance or virulence genes [7]. This emerging organism also exhibits outbreak potential necessitating surveillance of resistance profiles due to the increasing prevalence of Multidrug resistance (MDR) reports to several antibiotic classes including daptomycin [9].

Different toolboxes have been developed to analyze and describe pangenomes by cataloging and clustering genes and other genomic features. Different tools use different algorithms to identify gene families and to classify genes into core and accessory categories. Earlier tools depended on strict cutoffs of sequence identity thresholds to identify genes. There may arise the problem of over clustering or under clustering of gene families which may subsequently create a misleading impression about genome diversity and misidentify core and accessory genome composition. For this reason, we chose to assess the pangenome using three different bioinformatics approaches in order to be more reflective of the gene content and allelic diversity within the pangenome using different clustering algorithms.

The aim of this work is to describe and comprehensively analyze the pangenome structure of *C. striatum*. Such an analysis would provide valuable source of information on the species’ features and dynamics which provides a rich resource to understand its pathogenicity paving the way for better treatment and control strategies. Such information will also help to identify new drug targets to an organism exhibiting an increased MDR against which vancomycin, teicoplanin, and linezolid are considered the most active drugs [10, 11]. In addition, we aim to analyze the distribution of previously reported virulence factors and important antibiotic-resistance genes in order to understand the species’ dynamics, its relation to the host and its interaction with other commensal and pathogenic species. We also shed the light on the role of genomic islands, integrons and phages as agents of horizontal gene transfer in the species.

## Results

PIRATE output showed that the pangenome comprised 14,308 representative loci passed to blast. The loci clustered into 5253 gene families in the 310 analyzed genome sequences of which 2070 (39.4%) were considered core. Appendix A shows different gene clusters at the different thresholds analyzed. Because a steep increase in the number of unique clusters per threshold has been observed at 90%, this value has been used to identify the highest threshold at which core and accessory genome size can be determined using other tools.

Pangenome analysis using Roary revealed a total of 5857 genes composing the pangenome of the species at the 90% threshold. The number of core genes that are shared by 99–100% of strains was 1899 genes observed in 306 to < 310 strains while the soft core or near core cluster of accessory genes that are shared by 95% to < 99% of strains were 154 genes (Supplementary Fig. 1). The accessory set of gene clusters that are widely distributed in the population forming the shell genes were 963 genes shared by 15% to < 95% of the strains. The accessory set of genes that are rare in the population forming the cloud and occurring in 0% to < 15% of the strains showed the highest number of 2841 genes. These are also called unique genes (Supplementary Fig. 1).

PEPPAN output showed a total of 5614 genes composing the pangenome with 1967 gene clusters forming the strict core genes observed in 99%–100% of analyzed sequences. Soft core or near core clusters that are shared by 95% to < 99% of strains were 126 genes. The accessory set of genes forming the shell genes shared by 15% to < 95% of the strains were 1083 gene clusters while the cloud unique genes observed in 0% to < 15% of the strains were 2438 gene clusters in total.

The curves illustrated in Supplementary Fig. 5 and Supplementary Fig. 6 (Appendix A) indicate that the gene repertoire is open accepting new genes, however, the process may be limited as more sequenced genomes are added. Heaps’ law model as calculated using PEPPAN showed Gamma = 0.120 +/- 0.001 and Alpha = 1.009 +/- 0.004 with ~ 1.769–2.212 new genes per new genome and ~ 0.394 fewer core genes per new genome when genomes are ordered in a random way. Alpha ≤ 1 and 0 < gamma < 1 indicate an open pangenome. With the available sequences, this indicates that adding new genomes will not increase the number of new genes significantly. Because alpha is nearly = 1, it is concluded that the pangenome grows very slowly but it is still technically unbounded and may be still consistent with an open pangenome. This finding agrees with the finding of Jesus, H.N.R. et al., [12], however, slightly different values may be attributable to differences in the number and diversity of genome sequences studied and the different analysis approaches used.

**Fig. 1:**
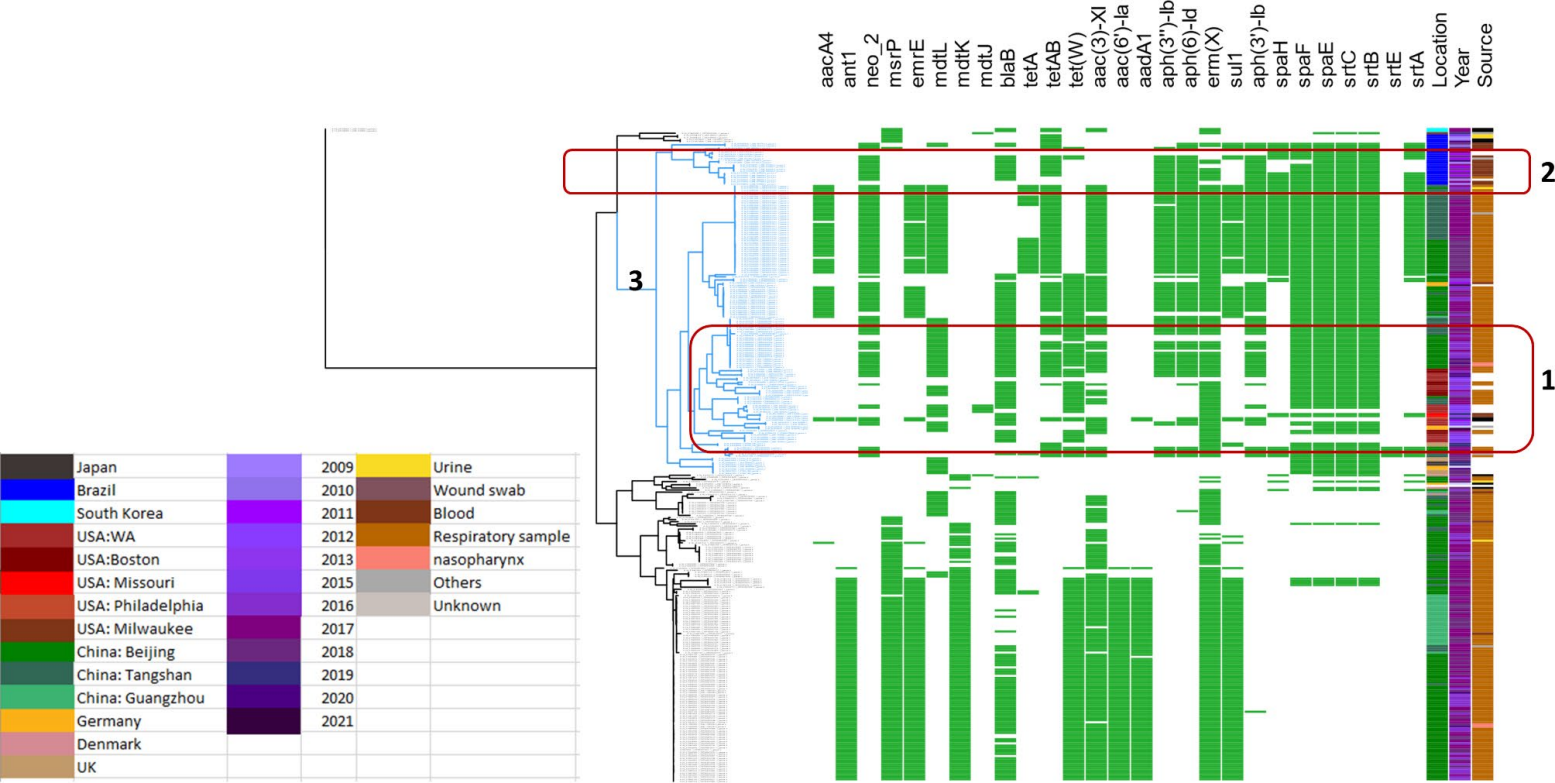
Distribution of resistance and virulence genes, year, source, and place of isolation in relation to the *C. striatum* core genome phylogenetic tree

The distribution of resistance and virulence genes, year, source, and place of isolation in relation to core genome phylogeny are shown in Fig. 1.

**Fig. 2:**
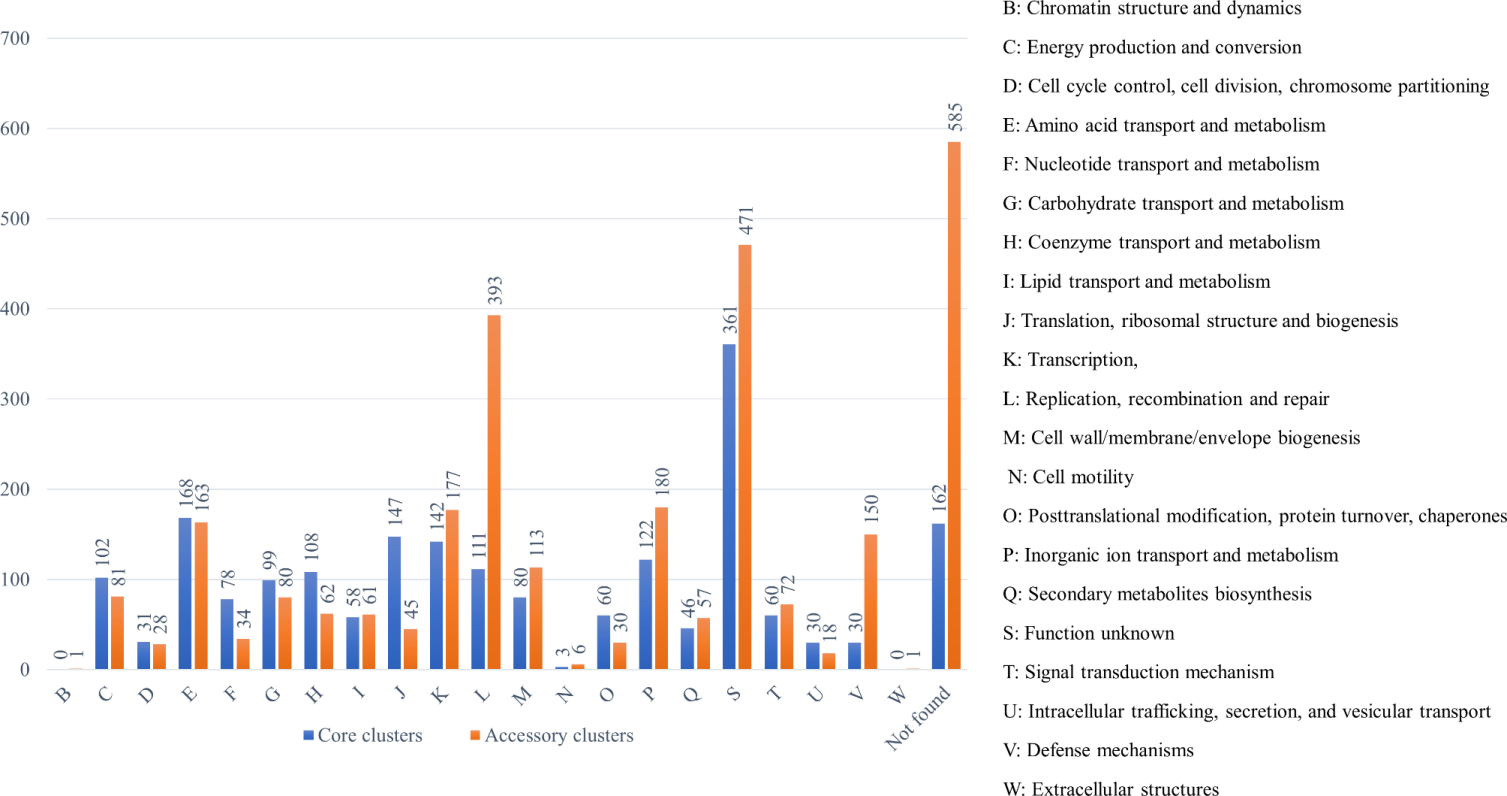
COG classes annotation for core and accessory clusters of *C. striatum* pangenome

1708 clusters were annotated among the core genome and 2058 clusters were annotated among the accessory genome using eggNOG (Fig. 2).

**Fig. 3:**
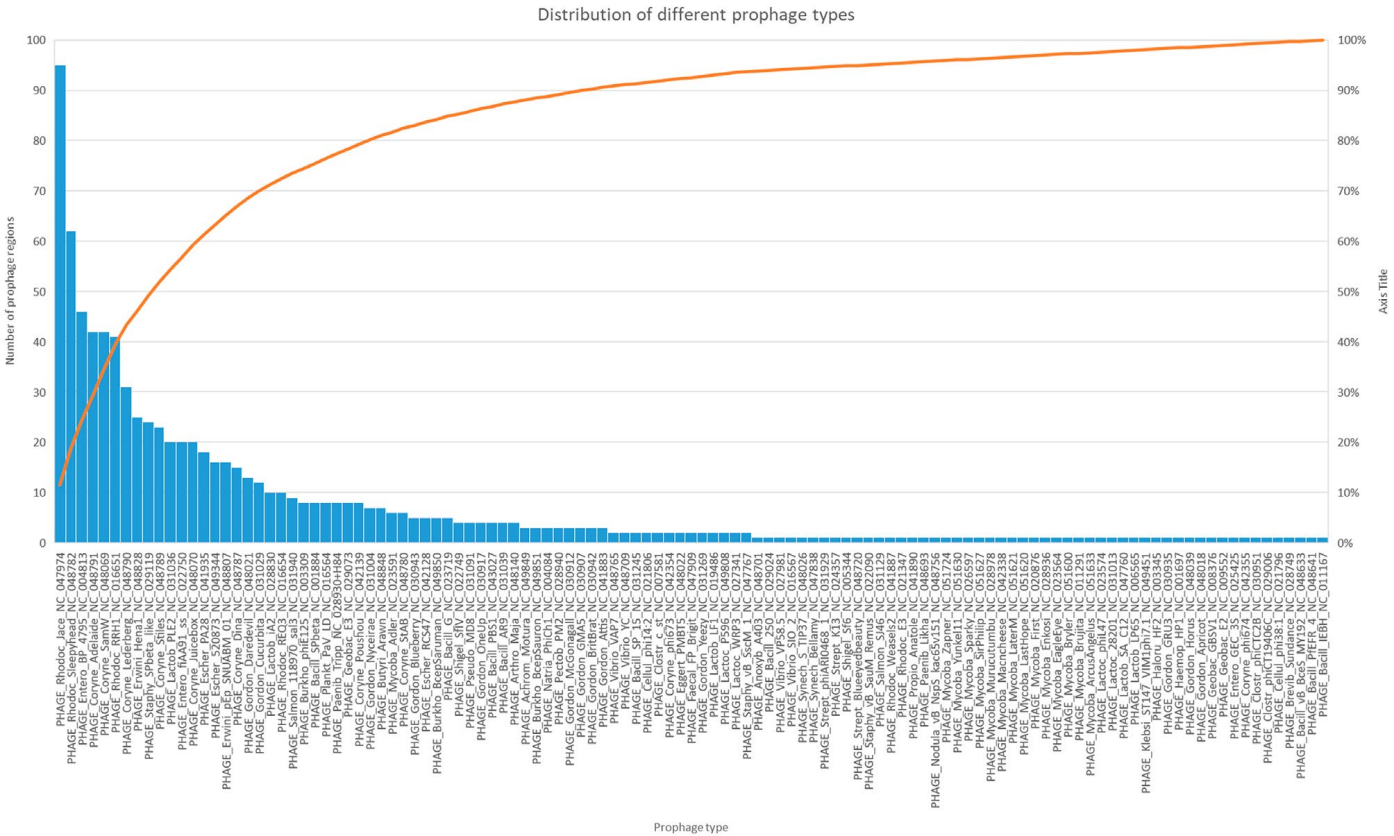
Most common types of identified phage sequences and their frequency among all studied 310 *C. striatum* genome sequences

A total of 115 phage species have been identified with 681 incomplete prophage sequences (PHASTER score < 70), 105 questionable prophage sequences (PHASTER score 70–90), and only 44 intact prophage sequences (PHASTER score > 90), including 20 different phage species, identified among all studied genomes. Most common types of identified phage sequences are shown in Fig. 3.

Prophage sequences showed carriage of antibiotic resistance genes as identified using ResFinder in only 6 of all studied genome sequences. These sequences were isolated from 3 different geographic locations including USA, UK, and Germany. Identified resistance genes carried on prophage region included *sul*1 (*sul*1_U12338), *sul*1 (*sul*1_AY963803), *aad*A1 (*aad*A1_JQ480156), *aac*(6’)-Ib-cr (*aac*(6’)-Ib-cr_EF636461), *aac*(6’)-Ib3 (*aac*(6’)-Ib3_X60321), *qac*E (*qac*E_X68232), *erm*(X) (*erm*(X)_U21300), *erm*(X) (*erm*(X)_X51472), *aph*(3’)-Ia (*aph*(3’)-Ia_X62115), *aph*(3’’)-Ib (*aph*(3’’)-Ib_AF321551), and *aph*(3’’)-Ib (*aph*(3’’)-Ib_AF321550). Each of these genes was identified in prophage regions from 2 genome sequences. Each of *aph*(3’’)-Ib (*aph*(3’’)-Ib_AF313472) and *aph*(3’’)-Ib (*aph*(3’’)-Ib_AF024602) was identified in prophage region from 1 sequence, and *cmx* (*cmx*_U85507) was identified in prophage regions from 4 sequences.

On the other hand, virulence genes were also infrequently present in prophage regions including the SpaD- type pilli *srt*C from *Corynebacterium* which was identified only in 1 prophage region among all studied genome sequences. Interestingly, sigma D (*sig*D) gene from *Mycobacterium* belonging to regulation virulence class genes was identified in prophage regions from 3 studied genome sequences while *sig*A/*rpo*V was identified in 9 prophage regions. ABC transporters: *fag*A, *fag*B, and *fag*C genes belonging to the iron uptake virulence class were identified among 4 prophage regions while *irt*A and *irt*B ABC transporters from *Mycobacterium* belonging to the same virulence class were identified in 2 prophage regions. Structural features of representative prophages carrying AMR genes are shown in Fig. 4A and 4B-4C.

**Fig. 4A:**
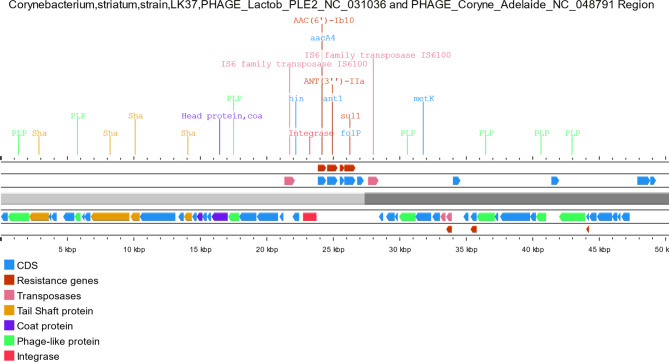
Structural features of prophage regions identified from RAQW01000001.1 *C. striatum* strain LK37 1 carrying PHAGE_Lactob_PLE2_NC_031036 and PHAGE_Coryne_Adelaide_NC_048791.

**Fig. 4B:**
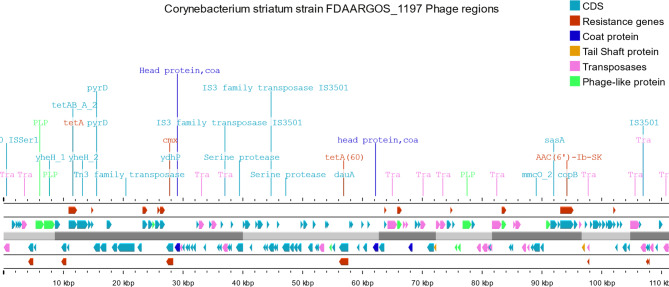
Structural features of prophage regions identified from CP069514.1 *Corynebacterium* striatum strain FDAARGOS_1197 carrying PHAGE_Escher_RCS47_NC_042128, PHAGE_Entero_fiAA91_ss_NC_022750, PHAGE_Staphy_sPbeta_like_NC_029119, PHAGE_Entero_BP_4795_NC_004813, and PHAGE_Entero_BP_4795_NC_004813.

**Fig. 4C:**
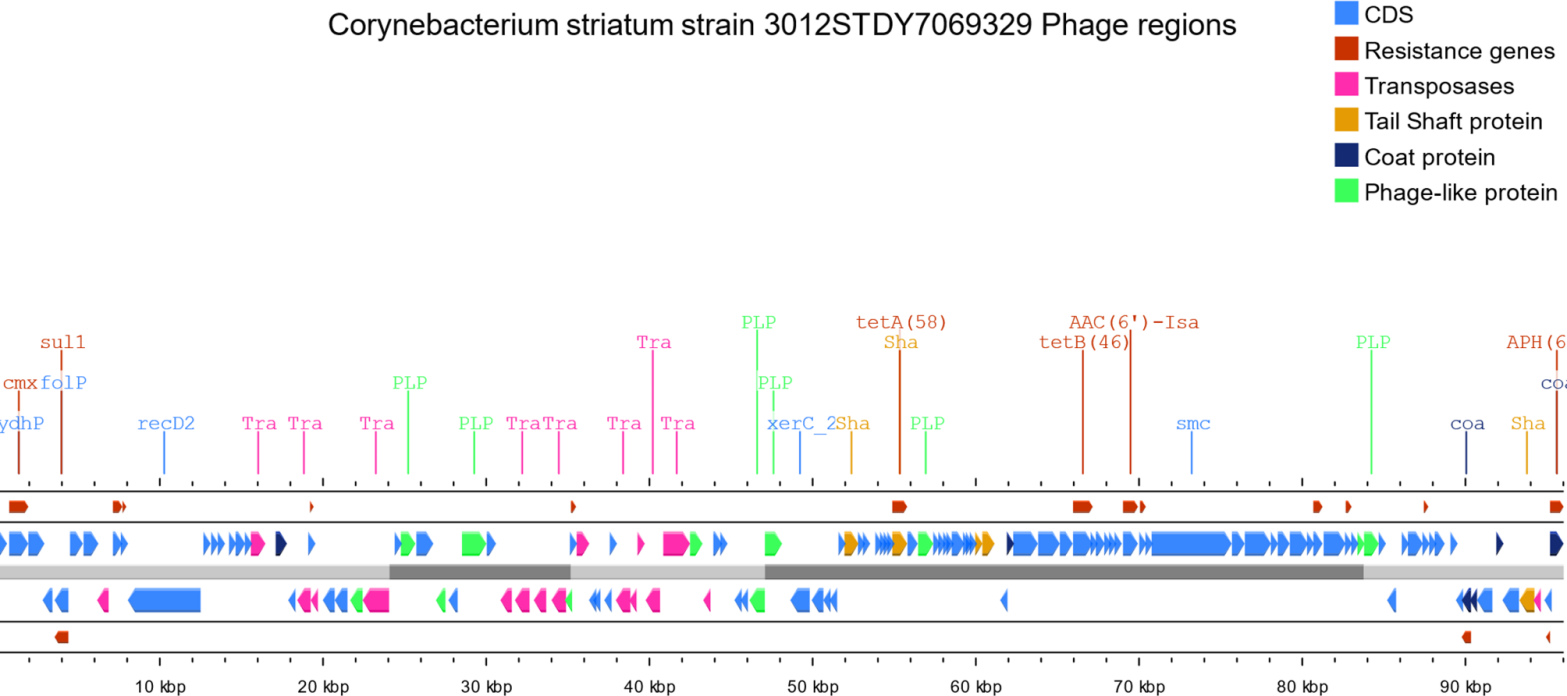
Structural features of prophage regions identified from NZ_CAACYF010000001.1 *Corynebacterium* striatum strain 3012STDY7069329 carrying PHAGE_Escher_RCS47_NC_042128, PHAGE_Rhodoc_Sleepyhead_NC_048782, PHAGE_Entero_fiAA91_ss_NC_022750, PHAGE_Coryne_Stiles_NC_048789, and PHAGE_Staphy_sPbeta_like_NC_029119

Class 1 integrons have been identified in 164 sequences with 9 sequences showing a predicted integron-integrases element lacking cassettes (In0) and 155 sequences showing complete integrons as predicted using IntegronFinder 2. A complete list of the identified integron elements is shown in Supplementary Table 2.

## Discussion

Results showed that the core genome of the species forms 35.05%–39.4% of the pangenome size using PEPPAN and PIRATE respectively. This is similar to the core genome ratio of *C. diphtheriae* that showed a core genome/pangenome ratio of 1632/4786 = 34.1% [13]. On the other hand, this percentage was higher in *C. pseudotuberculosis* which core genome contained 1504 genes representing 54% of the entire pan-genome of the species (2782 genes). [14] This might be expected due to different lifestyles of different species of the genus *Corynebacterium*. The pangenome of *C. striatum* appears to be very slowly growing showing even a slower rate of pan-genome expansion than *C. diphtheria* and *C. pseudotuberculosis* with higher alpha level than both. [14] Given the available sequence data and the current results, the pangenome of *C. striatum* appears to be of an open nature. The gene pool appears to be moderately expanding when more sequenced genomes are added. This may be attributed to the less specialized nature of the organism. It appears that the accessory genome set is moderately expanding as a result of the introduction of genes from other species offering a successful strategy for survival of bacterial species population to remain competitive in its environments [15].

The pangenome loci identified using PIRATE clustered into 5253 gene families while pangenome analysis using Roary revealed a total of 5857 genes composing the pangenome of the species at the 90% threshold. This difference may be expected due to post-processing paralog classification step applied by PIRATE which may result in the exclusion of some gene families [16]. PEPPAN [17] output showed a total of 5614 genes composing the pangenome. A possible reason for these differences in clustering between tools is most likely due to the different paralog-splitting methodologies used. PEPPAN provides consistent gene and pseudogene annotations and similarity-based gene predictions. It also identifies and excludes paralogs by combining tree- and synteny-based approaches.

Spa-type pili have been recognized as an important factor in host tissues colonization and virulence including biofilm formation. This has been observed in *C. striatum* as well as in other coryneform bacteria [18]. In *C. diphtheria*, three sortase-associated pilus gene clusters have been recognized and identified including *spa*HIG in addition to *spa*ABC and *spa*DEF [19]. 

In a recent study involving 22 *C. striatum* clinical isolates, *spa*D, *spa*E, and *spa*F genes have been detected in all isolates [20]. In the current analysis, sortase genes; *srt*A, *srt*B, *srt*C, and *srt*E have been detected in the accessory genome of the studied sequences. Megablastn has identified *srt*A at 81.3%–100% identity to *srt*A fimbrial associated sortase from *C. diphtheriae* CDCE 8392 (NCBI Gene ID: 11,706,508) in 69 genome sequences. The *srt*B has been identified at average 95% identity to DIP_RS12555 sortase from *C. diphtheriae* NCTC 13,129 (NCBI Gene ID: 2,650,495) in 159 genome sequences. Similarly, *srt*C has been identified at average 94% identity to *srt*C fimbrial associated sortase from *C. diphtheriae* CDCE 8392 (NCBI Gene ID: 11,707,567) in the same 159 sequences. The *srt*E has been identified in only 5 sequences at 77.5% identity similar to DIP_RS22240 sortase from *C. diphtheriae* NCTC 13,129 (NCBI Gene ID: 2,650,023).

Similarly, *spa*E has been identified in 159 sequences with average 90%–95% identity to surface-anchored protein fimbrial subunit from *C. diphtheriae* CDCE 8392 (NCBI Gene ID: 11,707,568). The *spa*H EP142_RS05550 from the SpaH/EbpB family LPXTG-anchored major pilin of *C. striatum* (NCBI Gene ID: 63,976,234) has been identified in 69 sequences at 81.3% to 100% identity. The *spa* F has been identified in 150 sequences with average 89% identity to *spa*F surface-anchored protein fimbrial subunit from *C. diphtheriae* CDCE 8392 (NCBI Gene ID: 11,707,569). On the other hand, neither of *spa*A, *spa*B, *spa*C, *spa*D, or *spa*G has been identified in any of the studied sequences. From the clinical and applied perspective, studying virulence genes within the pangenome context offers a valuable tool to select for therapeutic targets and based on the findings from the current analysis, each of *spa*F, *spa*H, *spa*E, and the sortase-pilin machinery appears to be frequently occurring in *C. striatum* making them good targets for anti-virulence agents.

In contrast to pili which seem to offer specific adhesion factors, the genus *Corynebacterium* shows a highly diverse composition and were isolated from different environments, including human and non-human hosts and it has been suggested that its pathogenicity can result from the so-called “niche factors” and a more general corynebacterial adhesion mechanism rather than a specific host–pathogen interaction [21]. Some of these niche factors did not show a proven role as virulence factors and may have a primary metabolic role that may function as a virulence factor or in virulence regulation under certain conditions. Because most of those genes may lack experimental validation of their specific role in virulence, those were not explored here. An example of that is the role of phospholipase D identified in *Corynebacterium ulcerans* and *C. pseudotuberculosis* which should be interpreted with caution as a virulence factor as it did not show to have a role in the interaction of the bacterium with epithelial cell lines [21].

Several nutritional and metabolic factors related to iron uptake and metabolism have been described in several *Corynebacterium* species including *C. diphtheriae, C. glutamicum, C. jeikeium, C. pseudotuberculosis,* and *C. efficiens* as virulence factors. Due to their importance and being listed in the VFDB, these were analyzed in the current study. These include the iron chelate uptake ABC transporter family; *fag*C, *fag*A, *fag*B, and *fag*D. Also, the ABC-type heme transporters; *hmu*T, *hmu*U, and *hmu*V, the Ciu iron uptake and siderophore biosynthesis system; *ciu*A, *ciu*B, *ciu*C, *ciu*D, *ciu*E, and the Siderophore-dependent iron uptake system, including *irp*6A, *irp*6B, and *irp*6C were investigated.

Hemin import ATP-binding protein HmuV has been identified in 308 sequences. Hemin transport system permease protein HmuU has also been identified in 308 isolates while HmuT has been identified in 292 isolates. 147 hits have been positive for *fag*A gene with percentage identity ranging between 70.4% and 71.429% to *fag*A from *Corynebacterium pseudotuberculosis* FRC41. The *fag*C has been universally identified in all studied strains with percentage identity for positive hits ranging between 68.919% and 82.979% to *fag*C from *Corynebacterium ulcerans* BR-AD22. For *fag*D, 284 hits have been positive with percentage identity ranging between 65.413% and 66.5% compared to *fag*D from *Corynebacterium pseudotuberculosis* FRC41. The *ciu*D gene from the siderophore biosynthesis system has been identified at the frequency of 291/310 with hits showing percentage identity between 73% and 74.157% to *ciu*D from *Corynebacterium pseudotuberculosis* CIP 52.97. Regarding the Siderophore-dependent iron uptake system, *irp*6A has been universally identified in all studied sequences with hits showing percentage identity ranging between 71% and 76.5% to *irp*6A from *Corynebacterium diphtheriae* C7. Similarly, *irp*6B has been universally identified with percentage identity between 65% and 74.655% similar to *Corynebacterium diphtheriae* C7. The *irp*6C has also been identified in all studied sequences with percentage identity ranging between 69.538% and 70.336% similar to *Corynebacterium aurimucosum* ATCC 700975. These results agree with the results shown by Jesus, H.N.R. et al., [12] and this may be expected for such important metabolic proteins.

On investigating the genomic islands, type II secretion protein F, GNAT family N-acetyltransferase, iron ABC transporter permease, and iron chelate uptake ABC transporter family permease subunit were the most frequently identified proteins related to virulence based on the annotation of IslandViewer results which were identified at 276, 212, 227, and 197 sequences respectively. Other virulence-related factors identified on the predicted genomic islands included type II toxin-antitoxin system RelE/ParE family toxin, iron-siderophore ABC transporter substrate-binding protein, class C sortase, type II toxin-antitoxin system Phd/YefM family antitoxin which were identified in 65, 50, 42, and 31 sequences respectively. In addition, SpaH/EbpB family LPXTG-anchored major pilin, SpaA isopeptide-forming pilin-related protein, type VII secretion integral membrane protein Ecc, antitoxin VbhA family protein, and type II toxin-antitoxin system VapC family toxin were identified less frequently in 22, 17, 17, 7, and 3 sequences respectively.

Both horizontal and vertical adaptation to antibiotic exposure have a role in shaping the pangenome of a bacterial species [22, 23]. In order to explore that, previously reported resistance elements have been studied in relation to the species pangenome composition. A diverse repertoire of mobilizable forms of resistance has been previously reported including the macrolide and lincosamide resistance gene, *erm*(X) and *erm*(B), the *tet*(W) tetracycline resistance gene, the efflux-pump encoding chloramphenicol-resistance gene *cmx*, *amp*C, *bla* gene encoding beta-lactamases and the aminoglycoside resistance genes, *aac*(3)-XI, *aph*(3”)-Ib, and *aph*(6)-Id [24, 25]. These elements have been linked to clinical resistance in other studies where the organism has exhibited susceptibility only to tetracycline, vancomycin and linezolid [10]. In addition, other studies have reported the occurrence of *aac*(6’)-Ia, *aad*A1, and *aph*(3’)-Ic conferring aminoglycoside resistance and also *cmx*, *str*A, *str*B, and *sul*1 that confer resistance against chloramphenicol, streptomycin and sulfamethoxazole [24, 26]. The * tet* AB gene encoding an ABC transporter has also been linked to beta-lactam and tetracycline resistance in the organism. [9].

In the current study set, aminoglycoside N-acetyltransferase *aac*(3)-XI was the most frequently identified acquired aminoglycoside resistance gene being identified in 231 sequences. All of these were identified on the predicted genomic islands. Erythromycin resistance leader peptide was identified in 216 sequences, all of which were also carried on the predicted GIs. This was followed by *aph*(3’)-Ib, *aph*(6)-Id, *ant*1, and *aph*(3’’)-Ib being identified in 112, 111, 100, and 104 sequences respectively. Also, *aad*A1, *aac*(6’)-Ia, and *aac*A4 have been identified in 102, 98, and 58 sequences respectively. All of the ANT(3’’)-Ia family aminoglycoside nucleotidyltransferase AadA1 genes and the aminoglycoside 6’-N-acetyltransferase AAC(6’)-Ia genes were identified on the predicted GIs being identified in 102 and 98 sequences respectively. Of the aminoglycoside O-phosphotransferase APH(3’’)-Ib, 93 were identified on the predicted GIs while for the aminoglycoside O-phosphotransferase APH(6)-Id, only 11 were located on the predicted GIs. The aminoglycoside O-phosphotransferase APH(3’)-Ia was identified in 104 sequences located on predicted GIs, while the APH(6)-I family aminoglycoside O-phosphotransferase was located on GIs in 74 sequences. Aminoglycoside N-acetyltransferase AAC(6’)-Ib and the APH(3’’) family aminoglycoside O-phosphotransferase were carried on genomic islands in 59 and 13 sequences respectively.

The macrolide and lincosamide resistance gene, *erm*(X), has been identified at a high frequency of 267 while *emr*E has been identified in 156 sequences. The 23 S rRNA (adenine(2058)-N(6))-methyltransferase Erm(X) gene was located on genomic islands in 220 sequences while the 23 S ribosomal RNA methyltransferase Erm was identified on the predicted GIs in 56 sequences. The *sul*1 conferring sulphonamide resistance has been identified in 165 sequences. Of those, 161 were identified on GIs. The tetracycline resistance genes *tet*(W), *tet*AB, and *tet*A have been identified in 177, 112, and 30 sequences respectively and almost all of them were located on GIs. The *bla* B gene encoding beta-lactamases has been identified in 192 sequences which were not identified on GIs. The multidrug resistance proteins; *mdt*K, *mdt*L, and *mdt*KJ have been identified in 122, 111, and 6 sequences respectively. On the predicted GIs, the multidrug efflux SMR transporter has been identified in 3 sequences. Chloramphenicol resistance genes have been identified on GIs including Cmx chloramphenicol efflux MFS transporter, chloramphenicol resistance leader peptide, and type B chloramphenicol O-acetyltransferase at the frequency of 102, 77, and 55 respectively. Quaternary ammonium compound efflux SMR transporter QacE delta 1 was carried on GIs in 157 of the studied sequences.

Although it has been reported that Class 1 integrons occur in several *Corynebacterium* clinical isolates, no data about the prevalence of integrons in *C. striatum* is available. A recent study has identified Class 1 integrons in 5/27 studied *C. striatum* sequences with 1 strain carrying the genes *sul1*, *qacE*, *aadA*, and *aac(6’)-lb7* and 4 other strains carrying only *sul1* [12]. In the current set of analyzed sequences, 155 complete integron elements have been predicted using IntegronFinder 2 (Supplementary Table 2).  The ANT(3’’)- *aad*A1-*aac*(6’)-Ia gene cassette has been identified in integrons from 97 studied sequences while the SMR *qac*E- *aac*(6’)-Ib gene cassette has been identified in 56 sequences. Integrons from 2 studied sequences carried the *aac*(6’)-Ib - ANT(3’’)- *aad*A1- SMR *qac*E gene cassette.

Based on the pangenome analysis, these results indicate that horizontally transferred acquired resistance elements specifically genomic islands carrying antimicrobial resistance genes appear to be relatively widespread in species supporting findings from previous studies and stressing on the need to identify new drug targets for such an emerging pathogen.

In Fig. 2, functional annotations of gene clusters belonging to both core and accessory genomes are visualized using the COG classification scheme which comprises 22 COG categories that are broadly divided into functions relating to cellular processes, signaling, information storage and processing, metabolism, and also genes which are poorly categorized [27]. The most prevalent gene families in the pan genome of *C. striatum* included those from replication, recombination and repair, amino acid and inorganic acid transport and metabolism, transcription, in addition to those with poorly characterized functions. Functional clusters showing higher frequency of core genes included C (energy production and conversion), E (amino acid transport and metabolism), F (nucleotide transport and metabolism), H (coenzyme transport and metabolism), J (translation, ribosomal structure and biogenesis), and O (post-translational modification, protein turnover, and chaperones) (Fig. 2). This result shows a similar expected finding as all of these COG functional categories showed significant enrichment in the core genome in at least 11/12 species in previous studies [28]. On the other hand, functional analysis of *C. striatum* COG categories showed more frequent occurrence of G (Carbohydrate transport and metabolism) in the core genome which was not observed in other species [28]. In addition, U (intracellular trafficking, secretion, and vesicular transport) was more frequent in *C. striatum* core genome which is contrary to findings from other studies showing enrichment of this category in 9/12 species accessory genomes [28]. Regarding accessory genome, it showed more frequent occurrence of V (defense mechanisms) which is consistent with similar finding showing enrichment of this category in 7/12 studied species [28]. In addition, *C. striatum* showed higher occurrence of P (Inorganic ion transport and metabolism), K (transcription), and L (Replication, recombination, and repair) among accessory gene clusters.

**Fig. 5:**
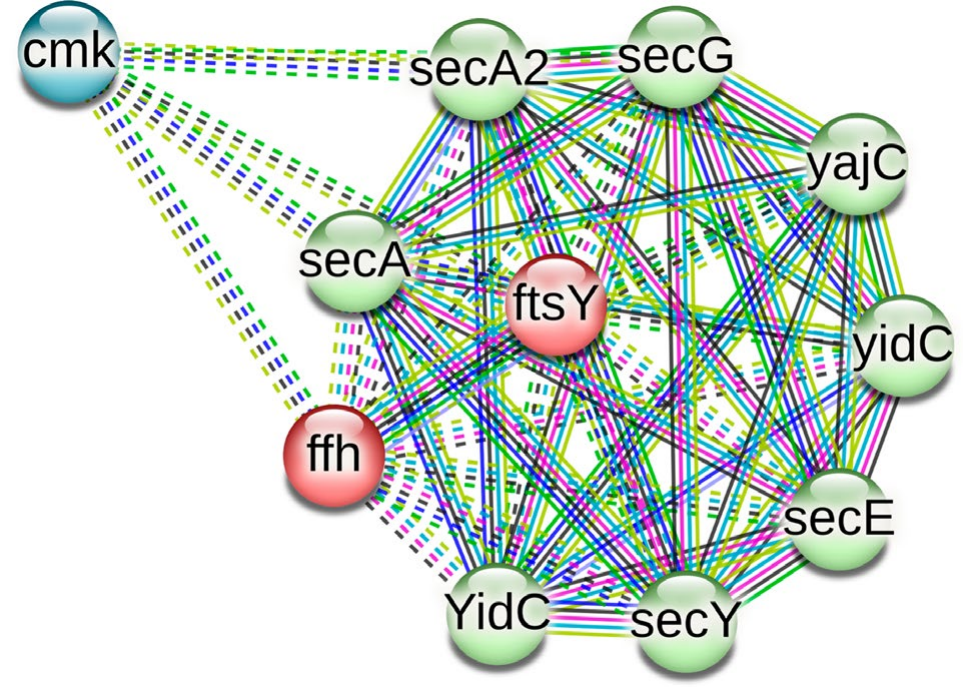
STRING network of some core genes belonging to U COG gene clusters in *C. striatum*

From these results, it can be observed that both G (Carbohydrate transport and metabolism) and U (intracellular trafficking, secretion, and vesicular transport) gene clusters occur more frequently in the core than the accessory genome of *C. striatum* which was not common in other species. Among the G COG class genes, several genes belonging to mannose and fructose metabolism pathway were occurring among the core genome of the organism including those with functions involved in the final step of synthesis of D-Mannose-6P and D-Fructose 6P as well as the isomerase involved in their interconversion. The resulting GDP-mannose is used in the synthesis of mannose-containing glycoconjugates that are important for mediating entry into host cells. Several enzymes essential for the degradation of carbohydrates via glycolysis were also occurring in the core genome as well as component of the phosphoenolpyruvate-dependent sugar phosphotransferase system (sugar PTS). Regarding the U COG class genes identified in the core genome including *ffh*, *fts*Y, *sec*A2, *sec*E, *sec*G, *sec*Y, *yaj*C, and *yid*C, STRING network analysis showed significant functional enrichment in bacterial secretion system, intracellular protein transmembrane transport, protein targeting and import, membrane insertase activity biologic process in the network, molecular functions, and local STRING network cluster including these genes (Fig. 5). Those genes in addition to *sec*F, *sec*D, *lep*B, *tat*A, *tat*B, and *tat*C form the annotated KEGG pathways bacterial secretion system and protein export.

Similar to the results shown by Jesus, H.N.R. et al., [12], analysis of the biosynthetic pathways of secondary metabolites revealed the capabilities of producing polyketides and several non-ribosomal peptides (NRPs). In addition, other secondary metabolite compounds have also been identified in the pangenome including terpene, aminopolycarboxylic-acid metallophores, Linear azoline-containing peptides, lassopeptide, and Linear arid peptide such as cypemycin (HQ148718) and salinipeptin (MG788286). Similar known clusters to the identified secondary metabolites regions included ε-Poly-L-lysine biosynthetic gene cluster from *Epichloe festucae*, cyclofaulknamycin biosynthetic gene cluster from *Streptomyces albidoflavus*, coelichelin biosynthetic gene cluster from *Streptomyces coelicolor A3(2)*, dehydroxynocardamine biosynthetic gene cluster from *Corynebacterium propinquum*, amychelin A biosynthetic gene cluster from *Amycolatopsis methanolica*, scabichelin biosynthetic gene cluster from *Streptomyces scabiei 87.22*, carotenoid biosynthetic gene cluster from *Corynebacterium glutamicum*, [S,S]-EDDS biosynthetic gene cluster from *Amycolatopsis japonica*, retimycin A biosynthetic gene cluster from *Salinispora arenicola CNT005*, HTTPCA biosynthetic gene cluster from *Photorhabdus laumondii subsp. Laumondii*, CDA1b biosynthetic gene cluster from *Streptomyces coelicolor A3(2)*, ulbactin F biosynthetic gene cluster from *Brevibacillus brevis*, and loseolamycin A1 biosynthetic gene cluster from *Micromonospora endolithica.* The NRP secondary metabolite belonging to Non-alpha poly-amino acids like e-Polylysin cluster was the most commonly identified in the pangenome and showed 100% similarity to ε-Poly-L-lysine biosynthetic gene cluster from *Epichloe festucae*. The compound has been identified to exhibit anti-fungal activity [29]. The ε-PL has been known as an antibacterial polyamine secreted mainly by some bacteria in the Streptomycetaceae family [30] and a few members of the Bacillaceae family [31] and is known as an antimicrobial agent with a wide inhibitory spectrum against Gram-positive bacteria, Gram- negative bacteria, fungi, yeasts, and phages [29]. Another commonly occurring NRP was the non-ribosomal peptide metallophores cluster similar to the coelichelin biosynthetic gene cluster from *Streptomyces coelicolor A3(2). Streptomyces coelicolor* is a soil dwelling filamentous bacteria producing many natural antibiotics and showing an ancient synteny with *Mycobacterium tuberculosis* and *Corynebacterium diphtheriae* [32]. Coelichelin is a NRP siderophore previously characterized from the genome of *Streptomyces coelicolor* [33]. Also, the zinc-dependent aminopolycarboxylic acid metallophore showing high similarity to ethylenediamine-disuccinate ([S,S]-EDDS) biosynthetic gene cluster from *Amycolatopsis japonica* has been identified in *C. striatum* pangenome. It appears that the possession of multiple NRPs and secondary metabolites with antimicrobial properties in addition to several metallophores offer the species a competitive advantage in its environment.

Epidemiologic and geographic diversity have been achieved in the current analysis by including sequences of isolates from different regions including different regions in China, USA, Brazil, South Korea, Germany, UK, Japan, and Denmark. The isolates spanned the time period between 1990–2021. Core genome phylogram showed variable degrees of regional clustering. Isolates from China showed a large degree of variation spanning the whole phylogenetic tree. Similarly, isolates from Germany appears to be diverse considering phylogenetic background. On the other hand, isolates from different USA regions were more closely related and occupied closely related lineages showing variable distribution of virulence and resistance elements. These isolates showed close relation to some isolates originating from China occupying the same phylogenetic subgroup (Fig. 1, **cluster 1**). These isolates were isolated in the time period between 1992 to 2021 and most of them were respiratory samples. Cluster 1 showed the co-occurrence of a group of virulence genes including *srt*B, *srt*C, *spa*E, and *spa*F and absence of *srt*E, *srt*A, and *spa*H. Most isolates observed in the cluster showed the carriage of *tet*W and lack of *sul*1. Similarly, Brazil isolates were highly clustered forming a separate phylogenetic group yet showed variable patterns of carriage of resistance and virulence genes (Fig. 1, **cluster 2**). Most of isolates in this cluster were isolated from blood samples and spanned the time period between 2009 to 2020. The co-occurrence of *srt*C, *spa*H, APH(3’’)-Ib, and *erm*(X) in this lineage may indicate the prevalence of genomic islands carrying these genes in this area. The *srt*E has only been identified in 5 isolates belonging to a phylogenetic cluster originating from 2 regions in China. These were isolated from sputum culture and one pleural effusion specimen. These were clustered together with the virulence genes; *srt*A, *srt*B, *srt*C, *spa*E, *spa*F, and *spa*H. These also clustered together with the resistance genes; *aph*(3’)-Ib, *aph*(6)-Id, *aph*(3’’)-Ib, *erm*(X), *aac*(3)-XI, *tet*(W), *tet*AB, and neo_2 indicating the co-occurrence and mobilization of some of these genetic elements on the same genomic islands as previously shown. The studied virulence genes occur over a large phylogenetic cluster (cluster 3 shown in blue color) which appears to be the most recent across phylogeny (Fig. 1). This may give an indication of the likely recent acquisition of these virulence factors from other species as a result of sympatric lifestyle. On the other hand, the aminoglycoside resistance genes including *ant*1, *aad*A1, *aac*(6’)-Ia and the multidrug resistance protein *mdt*K appear to be sparingly carried across earlier branches of phylogeny.

Plasmids are reported to be rare and phages have not been adequately studied in the species [9, 10, 34]. On the other hands, resistance in *C. striatum* has been linked to insertion sequences (IS) and transposons (Tn). [9] In this study, prophage regions showed the carriage of at least one virulence factor in sequences from 19 genomes while only 6 genomes have shown carriage of AMR genes on prophage elements. Exploring prophage regions within attachment sites has shown than AMR genes exist either between or near integrase and/or transposase genes (Fig. 4A and 4B-4C). These recombination elements may probably accommodate AMR genes which does not directly prove the hypothesis that AMR genes carried by prophage regions are acquired or transferred by phage transduction. These observations conclude that phage acquisition does not appear to be an important mechanism to acquire resistance and virulence determinants in the species. This may be supported by observations from previous studies. [35, 36]. As plasmids were previously reported to be rare and the current results also show that phages do not appear to be widely distributed in the species, it is concluded that insertion sequences (IS) and transposons (Tn) may play the main role in acquiring new genetic elements. Therefore, there is a concern about the species acting as nosocomial reservoir of mobilizable resistance to more pathogenic species by acquiring AMR and pathogenicity determinants from co-living species which consequently hinder the efforts of treating *C. striatum* related infection.

## Conclusion

The pangenome of *C. striatum* appears to be of an open nature and is slowly growing allowing the species to remain competitive in its environments. The tetracycline resistance genes *tet*(W), *tet*AB, *tet*A and the macrolide and lincosamide resistance genes, *erm*(X) and *erm*E, have been identified at the highest frequency followed by *aac*(3)-XI which has been the most frequently identified acquired aminoglycoside resistance gene. Genomic islands appear to offer a prominent role in mobilizing antibiotic resistance genes in the species while their role in virulence acquisition may be less important. Integrons occur in 50% of *C. striatum* isolates and they carry aminoglycoside resistance genes in addition to the Quaternary ammonium compound efflux SMR transporter QacE. The current results show that phages do not appear to be widely distributed in the species, and it is concluded that insertion sequences (IS) and transposons (Tn) may play the main role in acquiring new genetic elements. Therefore, there is a concern about the species acting as nosocomial reservoir of mobilizable resistance to more pathogenic species by acquiring AMR and pathogenicity determinants from co-living species. Interestingly, some virulence determinants carried on identified prophage regions appear to be acquired from *Mycobacterium*. Phylogenetic analysis points to the possibility of recent acquisition of different virulence factors from other species as a result of sympatric lifestyle. Based on the findings from the current pangenome analysis, each of *spa*F, *spa*H, *spa*E, and the sortase-pilin machinery appears to be frequently occurring in *C. striatum* making them good targets for anti-virulence agents. G (Carbohydrate transport and metabolism) COG category and U (intracellular trafficking, secretion, and vesicular transport) were more frequent in *C. striatum* core genome. We report that e-Polylysin NRP appears to be an important antimicrobial polypeptide that occurs at very high frequency in *C. striatum* pangenome. We also report the identification of coelichelin and aminopolycarboxylic acid metallophores in the pangenome of *C. striatum*. These elements appear to equip the organism with competitive capabilities.

## Methods

A panel of 310 *C. striatum* sequences representing different geotypes of the species and available at NCBI Genbank database has been studied. Information about sequences is provided in Supplementary Table 1. Genome sequences investigated originated from different geographic locations and spanned a wide timeline. The panel included assemblies submitted between years 1990–2021. Downloaded FASTA files for assembled contigs were first annotated using PROKKA [37]. To perform pangenome analysis, three pipelines have been implemented and different percentage identity matches were tested and compared. These included Roary [38], PIRATE [16], and PEPPAN [17]. PEPPAN was implemented using the default parameters of 0.9 identities and 0.8 align ratio and using PROKKA annotated GFF3 files as an input to the pipeline. PEPPAN_parser script was then applied to get the final output.

Using PIRATE, the pangenome was reconstructed using 8 threads with default MCL inflation value of 1.5 and a range of amino acids sequence similarity thresholds (50,70, 80, 90, and 95%) and applying the (–pan-off) option. Roary was then implemented using a command line tool in Linux environment to construct the pangenome. Default settings were used with blastp percentage identity run at 95%, 90%, 80%, and at 70% and using the (-s) option not to split paralogs.

ResFinder database of acquired antimicrobial resistance genes [39] has been used to identify the distribution of acquired resistance genes against different antibiotics including aminoglycosides, beta-lactams, glycopeptides, sulphonamides, tetracyclines, quinolones and trimethoprim in the pangenome of *C. striatum*. In addition, the pangenome annotation generated using Roary has been screened for other probable resistance-related proteins.

Virulence factors available at the virulence factors database of pathogenic bacteria [40] at http://www.mgc.ac.cn/cgi-bin/VFs/genus.cgi?Genus=Corynebacterium have been used to identify the distribution of virulence factors in the pangenome of *C. striatum*. In addition, a curated version of the PATRIC VFDB [41] with unique orthologs and proteins directly involved with virulence available at https://figshare.com/articles/dataset/Curated_Virulence_Factor_Database/8232935/1 has also been used to search the pangenome for other probable virulence factors.

Core gene alignment generated using Roary has been used to build a core genome-based phylogenetic tree using FastTree [42]. Distribution of resistance and virulence genes and metadata have been plotted against core-genome tree using Phandango [43]. The Class of Genes (COG) annotation of every protein belonging to each of core and accessory genome fractions was performed using eggNOG 4.5 server. [44]. In order to study the functional significance of the identified gene families, antiSMASH 7.0 webserver [45] has been used to predict Biosynthetic pathways of secondary metabolites. In addition, pathway analysis of enriched COG classes was explored using the KEGG pathway database [46] and the STRING version 11.5 database [47].

In order to study prophage regions, the custom web Application Programming Interface for PHASTER (**PHA**ge **S**earch **T**ool **E**nhanced **R**elease) [48] was used. Prophage names were identified using the most common phage species indicated by PHASTER. Detected regions were also classified into intact, questionable, or incomplete as identified by PHASTER score based on the proportion of phage genes in the identified region. Identified prophage sequences were further studied for carriage of antimicrobial resistance and virulence factors genes using the ResFinder database [39] and the Virulence Factors Database (VFDB) [49] respectively. Prophage regions carrying AMR genes were annotated using Prokka [37] and visualized using the CGView Server [50]. Prediction of genomic islands has been performed using IslandViewer 4 [51]. IntegronFinder 2.0 [52] has been used to identify integrons in *C. striatum* studied sequences.

### Electronic supplementary material

Below is the link to the electronic supplementary material.

Supplementary Material 1

Supplementary Material 2

## Data Availability

The datasets and analyses supporting the conclusions of this article are included within the article and its additional files. Information about genome sequences dataset analyzed for this study including assembly names and accessions are provided in Supplementary Table 1. The datasets analyzed in this study can be found free available in the NCBI database (https://www.ncbi.nlm.nih.gov/nucleotide/). Additional information and figures are provided in Supplementary Tables and in Appendix A.
