# Supplementary material for: Pangenome analysis of Corynebacterium striatum: insights into a neglected multidrug-resistant pathogen
Source: BMC Microbiol. 2023 Sep 8;23:252. doi: 10.1186/s12866-023-02996-6 (PMC10486106; doi:10.1186/s12866-023-02996-6)
Supplement: Supplementary file 1 — Supplementary Material 1 [file 12866_2023_2996_MOESM1_ESM.pdf]

Pangenome analysis of *Corynebacterium striatum*: Insights into a neglected multidrug-resistant pathogen

Appendix A

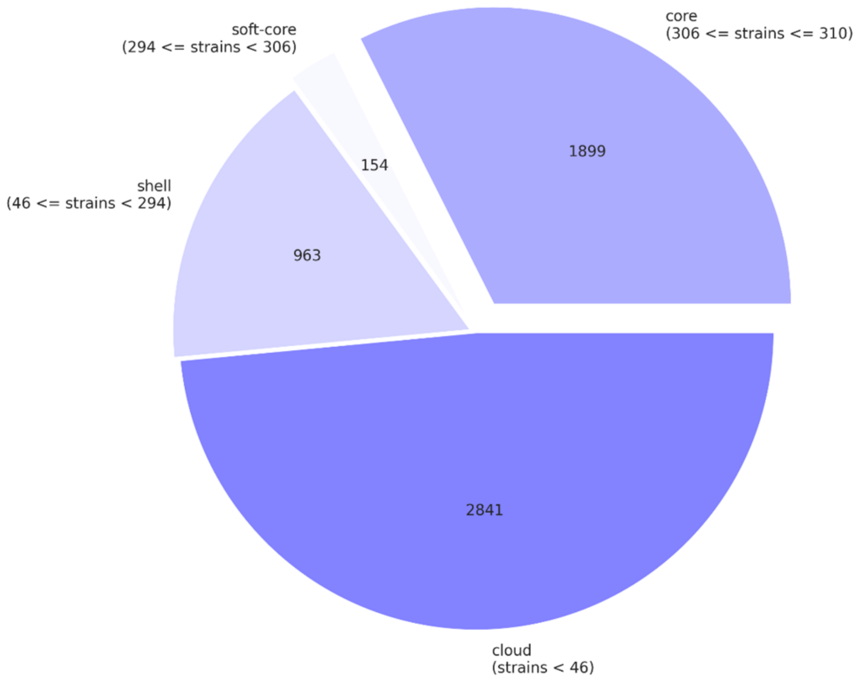

Supplementary Figure 1. Distribution of genes composing the pangenome of *C.striatum* using Roary at 90% threshold

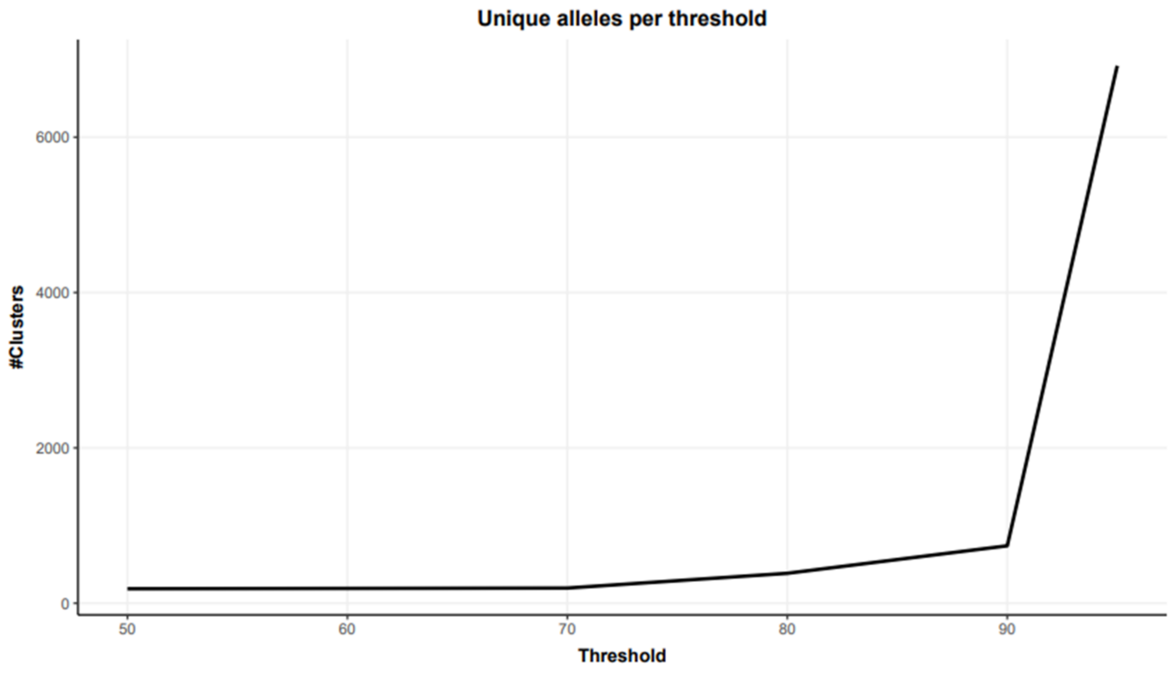

Supplementary Figure 2. Number of unique alleles per threshold

The number of unique alleles per genome sharply increased at percentage identity thresholds of >90% .

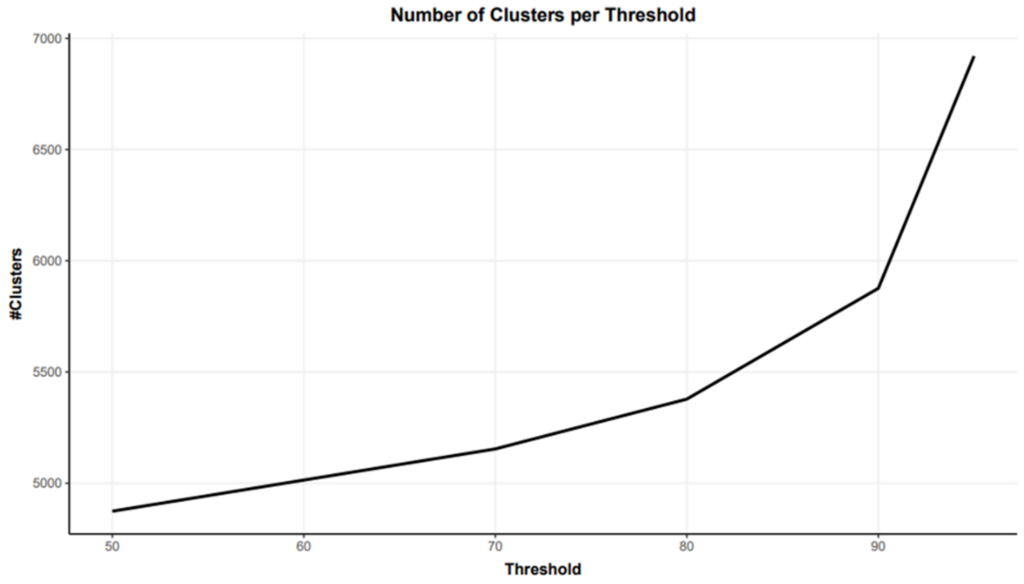

Supplementary Figure 3. Number of gene clusters per threshold

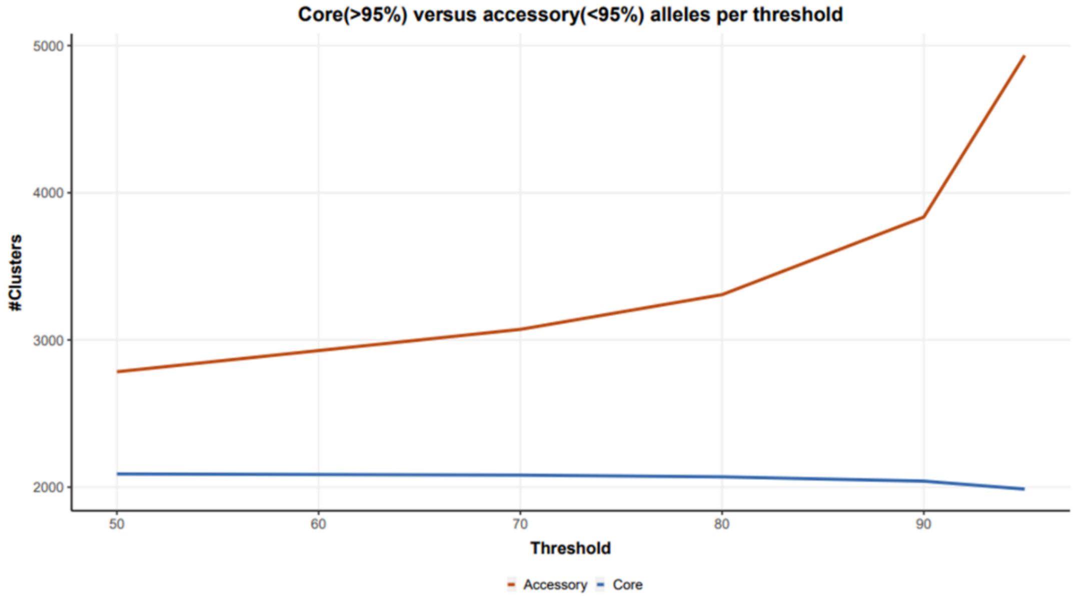

Supplementary Figure 4. Number of core and accessory alleles per threshold

Similarly, a marked increase in the number of gene clusters per threshold and in the number of accessory alleles per threshold was observed at the same percentage identity thresholds of >90% (Supplementary Figure 3 and Supplementary Figure 4). This may indicate that using higher threshold may lead to allelic variation beginning to influence identification of gene families.

Considering Supplementary Figure 3 and Supplementary Figure 4, it is observed that there is a noticeable increase in the number of clusters per threshold and in the number of accessory alleles per threshold which has been similarly observed in determining the pangenome size using Roary at the 80% threshold (-s) option. Pangenome analysis using Roary revealed that a total of 5322 genes composing the pangenome of the species at the 80% threshold with 1959 gene clusters forming the core genes observed in 99-100% of analyzed sequences. Soft core or near core cluster of accessory genes that are shared by 95 % to < 99 % of strains were 117 as observed at the same threshold. The accessory set of gene clusters that are widely distributed in the population forming the shell genes shared by 15 to < 95% of the strains were 900 gene clusters where the cloud genes observed in 0 % to < 15% of the strains were 2346 gene clusters in total.

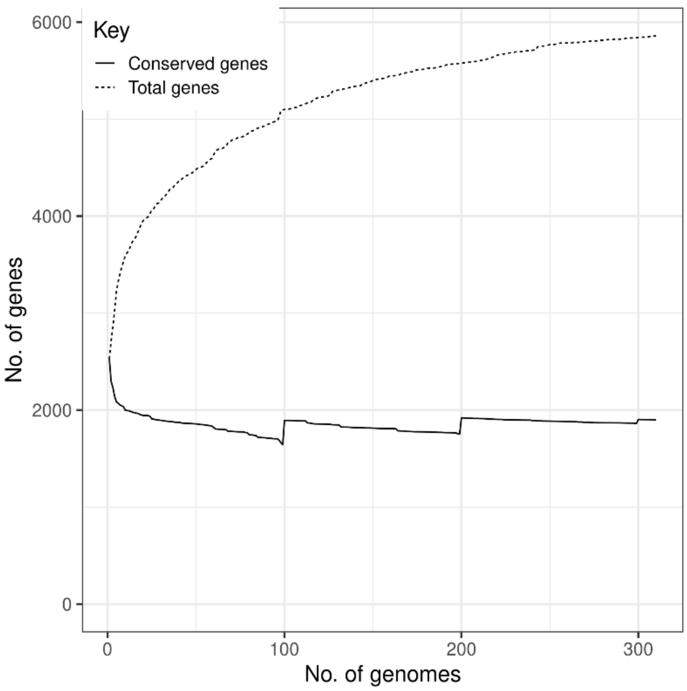

Supplementary Figure 5. Change in the number of total genes in the pangenome and in the number of conserved (core) genes as more genomes are added.

Supplementary Figure 5 shows that by the addition of new group members reaching about 300 genomes, the number of genes in the pangenome appears to show gradual increase approaching a plateau while the number of core genes appear to be relatively stable.

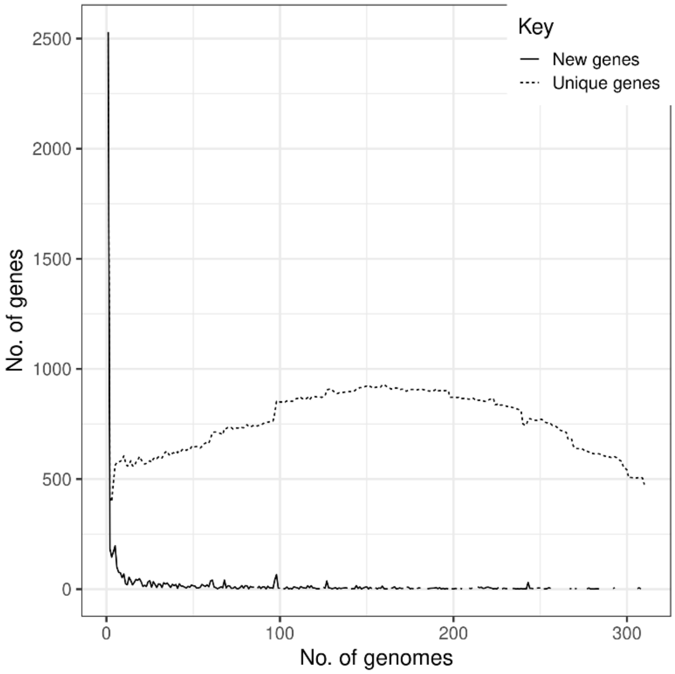

Supplementary Figure 6. Change in the number of unique genes as more genomes are added.

Supplementary Figure 6 also shows that the number of unique genes gradually increase till it levels off.
