# Supplementary material for: Pangenome analysis of Corynebacterium striatum: insights into a neglected multidrug-resistant pathogen
Source: BMC Microbiol. 2023 Sep 8;23:252. doi: 10.1186/s12866-023-02996-6 (PMC10486106; doi:10.1186/s12866-023-02996-6)
Supplement: Supplementary file 2 — Supplementary Material 2 [file 12866_2023_2996_MOESM2_ESM.pdf]

Pangenome analysis of *Corynebacterium striatum*: Insights into a neglected multidrug-resistant pathogen

Supplementary Material

Supplementary Table 1: Information about analyzed *C. striatum* genomes dataset

ID (Assembly accession_Assembly name)	Assembly Stats Total Sequence Length	Location	Collection Year
GCA_000988225.1_ASM98822v1_genomic	2611976	Brazil	2015
GCA_001053405.1_ASM105340v1_genomic	2914153	USA:WA	2015
GCA_001053435.1_ASM105343v1_genomic	2905202	USA:WA	2015
GCA_001055405.1_ASM105540v1_genomic	2784991	USA:WA	2015
GCA_001058455.1_ASM105845v1_genomic	2743259	USA:WA	2015
GCA_001059665.1_ASM105966v1_genomic	2834694	USA:WA	2015
GCA_001059955.1_ASM105995v1_genomic	2830184	USA:WA	2015
GCA_001076975.1_ASM107697v1_genomic	2829078	USA:WA	2015
GCA_002156805.1_ASM215680v1_genomic	2797692	South Korea: South sea	2017
GCA_002775055.1_ASM277505v1_genomic	2898882	Brazil: Rio de Janeiro	2017
GCA_002775095.1_ASM277509v1_genomic	2375104	Brazil: Rio de Janeiro	2017
GCA_002775105.1_ASM277510v1_genomic	2875402	Brazil: Rio de Janeiro	2017
GCA_002803965.1_ASM280396v1_genomic	2993983	USA:Boston	2017
GCA_002804085.1_ASM280408v1_genomic	3031488	USA:Boston	2017
GCA_002865925.1_ASM286592v1_genomic	2820414	Brazil: Rio de Janeiro	2018
GCA_003202245.1_ASM320224v1_genomic	3052429	Brazil: Rio de Janeiro	2018
GCA_003202295.1_ASM320229v1_genomic	2861961	Brazil: Rio de Janeiro	2018
GCA_003202335.1_ASM320233v1_genomic	3003571	Brazil: Rio de Janeiro	2018
GCA_003202355.1_ASM320235v1_genomic	2847990	Brazil: Rio de Janeiro	2018
GCA_003202375.1_ASM320237v1_genomic	3128097	Brazil: Rio de Janeiro	2018
GCA_004138065.1_ASM413806v1_genomic	2824576	USA: Missouri	2019
GCA_006538485.1_ASM653848v1_genomic	3113921	Japan	2019
GCA_008373895.1_ASM837389v1_genomic	2824107	USA: Philadelphia	2019
GCA_011682935.1_ASM1168293v1_genomic	2981342	China: Beijing	2020
GCA_011682945.1_ASM1168294v1_genomic	2937252	China: Beijing	2020
GCA_011682965.1_ASM1168296v1_genomic	2796099	China: Beijing	2018
GCA_011682985.1_ASM1168298v1_genomic	2788964	China: Beijing	2018

GCA_014610855.1_ASM1461085v1_genomic	2840792	Brazil: Rio de Janeiro	2009
GCA_014610885.1_ASM1461088v1_genomic	2841405	Brazil: Rio de Janeiro	2020
GCA_015889045.1_PDT000902873.1_genomic	2947021	China: Beijing	2016
GCA_015889065.1_PDT000902871.1_genomic	2941690	China: Tangshan	2017
GCA_015889085.1_PDT000902868.1_genomic	2762000	China: Beijing	2016
GCA_015889105.1_PDT000902870.1_genomic	2931351	China: Beijing	2016
_GCA_015889125.1_PDT000902869.1_genomic	2792149	China: Beijing	2016
GCA_015889145.1_PDT000902867.1_genomic	2754193	China: Beijing	2016
GCA_015889225.1_PDT000902826.1_genomic	2742456	China: Guangzhou	2018
GCA_015889245.1_PDT000902827.1_genomic	2740890	China: Guangzhou	2018
GCA_015889265.1_PDT000902829.1_genomic	2755508	China: Guangzhou	2018
GCA_015889285.1_PDT000902821.1_genomic	2946700	China: Beijing	2016
GCA_015889305.1_PDT000902832.1_genomic	2847446	China: Guangzhou	2017
GCA_015889325.1_PDT000902863.1_genomic	2795769	China: Beijing	2016
GCA_015889345.1_PDT000902865.1_genomic	2791693	China: Beijing	2016
GCA_015889355.1_PDT000902823.1_genomic	2723028	China: Guangzhou	2018
GCA_015889385.1_PDT000902830.1_genomic	2757507	China: Guangzhou	2018
GCA_015889405.1_PDT000902833.1_genomic	2941165	China: Tangshan	2017
GCA_015889425.1_PDT000902860.1_genomic	2773944	China: Tangshan	2018
GCA_015889445.1_PDT000902864.1_genomic	2770656	China: Beijing	2016
GCA_015889455.1_PDT000902825.1_genomic	2792740	China: Guangzhou	2017
GCA_015889485.1_PDT000902828.1_genomic	2747114	China: Guangzhou	2018
GCA_015889505.1_PDT000902862.1_genomic	3030962	China: Tangshan	2018
GCA_015889525.1_PDT000902859.1_genomic	3036287	China: Tangshan	2018
GCA_015889535.1_PDT000902831.1_genomic	2757367	China: Guangzhou	2017
GCA_015889565.1_PDT000902850.1_genomic	2799095	China: Guangzhou	2017
GCA_015889575.1_PDT000902861.1_genomic	3029392	China: Tangshan	2018
GCA_015889605.1_PDT000902858.1_genomic	2988008	China: Tangshan	2018
GCA_015889625.1_PDT000902851.1_genomic	2666454	China: Beijing	2016
GCA_015889645.1_PDT000902852.1_genomic	2790811	China: Guangzhou	2017
GCA_015889655.1_PDT000902857.1_genomic	2979043	China: Tangshan	2018
GCA_015889685.1_PDT000902853.1_genomic	2752741	China: Tangshan	2018
GCA_015889705.1_PDT000902855.1_genomic	3023407	China: Tangshan	2018
GCA_015889725.1_PDT000902854.1_genomic	2749582	China: Tangshan	2018
GCA_015889745.1_PDT000902849.1_genomic	2776618	China: Beijing	2017
GCA_015889765.1_PDT000902848.1_genomic	2783349	China: Beijing	2016
GCA_015889785.1_PDT000902847.1_genomic	2793363	China: Beijing	2016
GCA_015889805.1_PDT000902846.1_genomic	2792502	China: Beijing	2016
GCA_015889825.1_PDT000902844.1_genomic	3018867	China: Tangshan	2017
GCA_015889845.1_PDT000902845.1_genomic	2951101	China: Beijing	2016
GCA_015889865.1_PDT000902836.1_genomic	3162616	China: Tangshan	2017

GCA_015889885.1_PDT000902841.1_genomic	3001564	China: Tangshan	2017
GCA_015889905.1_PDT000902837.1_genomic	2968551	China: Tangshan	2017
GCA_015889925.1_PDT000902835.1_genomic	2937154	China: Tangshan	2017
GCA_015889945.1_PDT000902843.1_genomic	2995058	China: Tangshan	2017
GCA_015889965.1_PDT000902820.1_genomic	2850292	China: Beijing	2016
GCA_015889985.1_PDT000902818.1_genomic	2934517	China: Beijing	2016
GCA_015890005.1_PDT000902819.1_genomic	2947532	China: Beijing	2016
GCA_015890025.1_PDT000902815.1_genomic	2802502	China: Beijing	2016
GCA_015890045.1_PDT000902816.1_genomic	2794389	China: Beijing	2016
GCA_015890065.1_PDT000902814.1_genomic	2947835	China: Beijing	2016
GCA_015890085.1_PDT000902800.1_genomic	2967202	China: Tangshan	2017
GCA_015890105.1_PDT000902813.1_genomic	2793601	China: Beijing	2016
GCA_015890125.1_PDT000902811.1_genomic	2791181	China: Beijing	2016
GCA_015890145.1_PDT000902810.1_genomic	2777436	China: Beijing	2017
GCA_015890165.1_PDT000902809.1_genomic	2787368	China: Beijing	2017
GCA_015890185.1_PDT000902807.1_genomic	2793111	China: Tangshan	2017
GCA_015890205.1_PDT000902812.1_genomic	2801065	China: Beijing	2016
GCA_015890225.1_PDT000902806.1_genomic	2921057	China: Tangshan	2017
GCA_015890245.1_PDT000902808.1_genomic	2795693	China: Tangshan	2017
GCA_015890265.1_PDT000902804.1_genomic	3027292	China: Tangshan	2017
GCA_015890285.1_PDT000902803.1_genomic	3011799	China: Tangshan	2017
GCA_015890305.1_PDT000902801.1_genomic	3139038	China: Tangshan	2017
GCA_015890325.1_PDT000902802.1_genomic	2973755	China: Tangshan	2017
GCA_015890345.1_PDT000902799.1_genomic	2941786	China: Tangshan	2017
GCA_015890365.1_PDT000902798.1_genomic	2939489	China: Beijing	2016
GCA_015890375.1_PDT000902797.1_genomic	2787947	China: Beijing	2016
GCA_015890405.1_PDT000902796.1_genomic	2877725	China: Beijing	2016
GCA_015890425.1_PDT000902795.1_genomic	2743660	China: Beijing	2016
GCA_015890445.1_PDT000902794.1_genomic	2707965	China: Beijing	2016
GCA_015890465.1_PDT000902792.1_genomic	2887728	China: Beijing	2016
GCA_015890485.1_PDT000902793.1_genomic	2861238	China: Beijing	2016
GCA_015890505.1_PDT000902790.1_genomic	2939451	China: Beijing	2017
GCA_015890525.1_PDT000902791.1_genomic	2959412	China: Beijing	2016
GCA_015890545.1_PDT000902789.1_genomic	2902587	China: Tangshan	2017
GCA_015890565.1_PDT000902785.1_genomic	2786343	China: Tangshan	2018
GCA_015890585.1_PDT000902786.1_genomic	2758473	China: Tangshan	2018
GCA_015890605.1_PDT000902788.1_genomic	2721875	China: Tangshan	2017
GCA_015890625.1_PDT000902784.1_genomic	2761001	China: Tangshan	2018
GCA_015890645.1_PDT000902783.1_genomic	2799113	China: Guangzhou	2018
GCA_015890655.1_PDT000902782.1_genomic	2756662	China: Guangzhou	2017
GCA_015890685.1_PDT000902773.1_genomic	2730425	China: Tangshan	2017

GCA_015890705.1_PDT000902753.1_genomic	2934897	China: Beijing	2017
GCA_015890725.1_PDT000902756.1_genomic	2948373	China: Beijing	2016
GCA_015890745.1_PDT000902747.1_genomic	2741867	China: Guangzhou	2017
GCA_015890765.1_PDT000902745.1_genomic	2932089	China: Guangzhou	2017
GCA_015890785.1_PDT000902779.1_genomic	2920778	China: Tangshan	2017
GCA_015890805.1_PDT000902751.1_genomic	2758776	China: Guangzhou	2017
GCA_015890825.1_PDT000902746.1_genomic	2798207	China: Guangzhou	2017
GCA_015890845.1_PDT000902752.1_genomic	2914002	China: Tangshan	2017
GCA_015890865.1_PDT000902754.1_genomic	2859716	China: Beijing	2016
GCA_015890885.1_PDT000902778.1_genomic	2758855	China: Beijing	2017
GCA_015890905.1_PDT000902755.1_genomic	2792036	China: Beijing	2016
GCA_015890925.1_PDT000902767.1_genomic	2754597	China: Guangzhou	2018
GCA_015890945.1_PDT000902768.1_genomic	2757683	China: Guangzhou	2018
GCA_015890965.1_PDT000902766.1_genomic	2845566	China: Guangzhou	2018
GCA_015890985.1_PDT000902775.1_genomic	2776254	China: Tangshan	2017
GCA_015891005.1_PDT000902765.1_genomic	2758702	China: Guangzhou	2018
GCA_015891025.1_PDT000902769.1_genomic	2759581	China: Guangzhou	2018
GCA_015891045.1_PDT000902764.1_genomic	2794798	China: Guangzhou	2018
GCA_015891065.1_PDT000902777.1_genomic	3006385	China: Tangshan	2017
GCA_015891085.1_PDT000902776.1_genomic	2915986	China: Tangshan	2017
GCA_015891105.1_PDT000902763.1_genomic	2763199	China: Guangzhou	2018
GCA_015891125.1_PDT000902762.1_genomic	2759738	China: Guangzhou	2018
GCA_015891145.1_PDT000902770.1_genomic	2787973	China: Guangzhou	2018
GCA_015891165.1_PDT000902748.1_genomic	2824173	China: Guangzhou	2017
GCA_015891185.1_PDT000902759.1_genomic	2794608	China: Beijing	2016
GCA_015891205.1_PDT000902744.1_genomic	2758673	China: Guangzhou	2017
GCA_015891225.1_PDT000902760.1_genomic	2934036	China: Beijing	2017
GCA_015891245.1_PDT000902758.1_genomic	2793099	China: Beijing	2016
GCA_015891265.1_PDT000902757.1_genomic	2745844	China: Beijing	2016
GCA_015891285.1_PDT000902742.1_genomic	2789325	China: Guangzhou	2017
GCA_015891305.1_PDT000902739.1_genomic	2976430	China: Tangshan	2017
GCA_015891325.1_PDT000902741.1_genomic	2746756	China: Guangzhou	2017
GCA_015891345.1_PDT000902740.1_genomic	2946251	China: Tangshan	2017
GCA_015891365.1_PDT000902743.1_genomic	2846330	China: Beijing	2017
GCA_015891385.1_PDT000902736.1_genomic	2940037	China: Tangshan	2018
GCA_015891405.1_PDT000902737.1_genomic	2832483	China: Beijing	2017
GCA_015891425.1_PDT000902733.1_genomic	2832500	China: Tangshan	2018
GCA_015891445.1_PDT000902734.1_genomic	2837496	China: Tangshan	2018
GCA_015891465.1_PDT000902735.1_genomic	2880897	China: Tangshan	2018
GCA_015891485.1_PDT000902728.1_genomic	2838227	China: Beijing	2017
GCA_015891505.1_PDT000902731.1_genomic	3020699	China: Tangshan	2017

GCA_015891525.1_PDT000902730.1_genomic	2931847	China: Beijing	2017
GCA_015891545.1_PDT000902732.1_genomic	2789520	China: Tangshan	2018
GCA_015891565.1_PDT000902726.1_genomic	2748104	China: Beijing	2017
GCA_015891585.1_PDT000902709.1_genomic	2977102	China: Tangshan	2017
GCA_015891605.1_PDT000902727.1_genomic	2774565	China: Beijing	2016
GCA_015891665.1_PDT000902707.1_genomic	2800415	China: Beijing	2017
GCA_015891685.1_PDT000902725.1_genomic	2794574	China: Beijing	2016
GCA_015891745.1_PDT000902721.1_genomic	2771346	China: Beijing	2016
GCA_015891765.1_PDT000902723.1_genomic	2789344	China: Beijing	2016
GCA_015891785.1_PDT000902722.1_genomic	2912958	China: Beijing	2016
GCA_015891805.1_PDT000902718.1_genomic	2946139	China: Beijing	2016
GCA_015891815.1_PDT000902720.1_genomic	2776751	China: Beijing	2016
GCA_015891845.1_PDT000902719.1_genomic	2792148	China: Beijing	2016
GCA_015891865.1_PDT000902716.1_genomic	2796009	China: Beijing	2017
GCA_015891885.1_PDT000902715.1_genomic	3091011	China: Tangshan	2018
GCA_015891905.1_PDT000902717.1_genomic	2805536	China: Beijing	2017
GCA_015891925.1_PDT000902714.1_genomic	3020217	China: Tangshan	2018
GCA_015891945.1_PDT000902713.1_genomic	3025144	China: Tangshan	2018
GCA_015891965.1_PDT000902710.1_genomic	2970014	China: Tangshan	2018
GCA_015891985.1_PDT000902704.1_genomic	3017172	China: Tangshan	2018
GCA_015892005.1_PDT000902711.1_genomic	3001879	China: Tangshan	2017
GCA_015892025.1_PDT000902702.1_genomic	2895712	USA:WA	2020
GCA_015892045.1_PDT000902706.1_genomic	3030047	China: Tangshan	2018
GCA_015892065.1_PDT000902705.1_genomic	2890617	China: Tangshan	2017
GCA_015892105.1_PDT000902701.1_genomic	2893277	USA:WA	2020
GCA_015892295.1_PDT000902330.1_genomic	2616416	USA: Milwaukee, WI	2019
GCA_015913055.1_PDT000902729.1_genomic	2797691	China: Beijing	2017
GCA_015944615.1_PDT000670976.1_genomic	2876868	China: Beijing	2017
GCA_015944675.1_PDT000670986.1_genomic	2896244	China: Beijing	2018
GCA_015944695.1_PDT000670983.1_genomic	2908598	China: Beijing	2018
GCA_015944715.1_PDT000670982.1_genomic	2772214	China: Beijing	2018
GCA_015944735.1_PDT000670981.1_genomic	2914383	China: Beijing	2018
GCA_015944755.1_PDT000670972.1_genomic	2774572	China: Beijing	2017
GCA_015944775.1_PDT000670987.1_genomic	2913460	China: Beijing	2018
GCA_015944795.1_PDT000670988.1_genomic	2908489	China: Beijing	2018
GCA_015944815.1_PDT000670974.1_genomic	2937700	China: Beijing	2017
GCA_015945195.1_PDT000670990.1_genomic	2914536	China: Beijing	2018
GCA_015945255.1_PDT000670980.1_genomic	2810274	China: Beijing	2017
GCA_015945275.1_PDT000670985.1_genomic	2789383	China: Beijing	2018
GCA_015945295.1_PDT000670984.1_genomic	2913021	China: Beijing	2018
GCA_015945355.1_PDT000670939.1_genomic	2778880	China: Beijing	2017

GCA_015945475.1_PDT000670955.1_genomic	2831079	China: Beijing	2017
GCA_015945535.1_PDT000670953.1_genomic	2728621	China: Beijing	2017
GCA_015945575.1_PDT000670951.1_genomic	2737148	China: Beijing	2017
GCA_015945595.1_PDT000670952.1_genomic	2917209	China: Beijing	2017
GCA_015945635.1_PDT000670971.1_genomic	2790756	China: Beijing	2017
GCA_015945675.1_PDT000670954.1_genomic	2772417	China: Beijing	2017
GCA_015945695.1_PDT000670970.1_genomic	2915821	China: Beijing	2018
GCA_015945715.1_PDT000670966.1_genomic	2890247	China: Beijing	2017
GCA_015945735.1_PDT000670968.1_genomic	2768792	China: Beijing	2017
GCA_015945755.1_PDT000670950.1_genomic	2780497	China: Beijing	2017
GCA_015945775.1_PDT000670964.1_genomic	2785208	China: Beijing	2017
GCA_015945785.1_PDT000670965.1_genomic	2812206	China: Beijing	2017
GCA_015945795.1_PDT000670963.1_genomic	2751574	China: Beijing	2017
GCA_015945805.1_PDT000670967.1_genomic	2791270	China: Beijing	2017
GCA_015945815.1_PDT000670962.1_genomic	3128494	China: Beijing	2017
GCA_015945875.1_PDT000670961.1_genomic	2789842	China: Beijing	2017
GCA_015945895.1_PDT000670959.1_genomic	2742959	China: Beijing	2017
GCA_015945915.1_PDT000670960.1_genomic	2757771	China: Beijing	2017
GCA_015945935.1_PDT000670958.1_genomic	2750919	China: Beijing	2017
GCA_015945955.1_PDT000670948.1_genomic	2776264	China: Beijing	2017
GCA_015945985.1_PDT000670949.1_genomic	2916665	China: Beijing	2017
GCA_015946005.1_PDT000670947.1_genomic	2940564	China: Beijing	2017
GCA_015946025.1_PDT000670942.1_genomic	2845043	China: Beijing	2017
GCA_015946045.1_PDT000670945.1_genomic	3116533	China: Beijing	2017
GCA_015946065.1_PDT000670946.1_genomic	2746524	China: Beijing	2017
GCA_015946085.1_PDT000670943.1_genomic	2820548	China: Beijing	2017
GCA_015946105.1_PDT000670940.1_genomic	2773261	China: Beijing	2017
GCA_015946145.1_PDT000670941.1_genomic	2821234	China: Beijing	2017
GCA_015946165.1_PDT000670944.1_genomic	3097981	China: Beijing	2017
GCA_015946265.1_PDT000670956.1_genomic	2704918	China: Beijing	2017
GCA_015946325.1_PDT000670957.1_genomic	2707159	China: Beijing	2017
GCA_015946445.1_PDT000670938.1_genomic	2821051	China: Beijing	2017
GCA_015946465.1_PDT000670934.1_genomic	2898144	China: Beijing	2017
GCA_015946485.1_PDT000670933.1_genomic	2772926	China: Beijing	2017
GCA_015946505.1_PDT000670916.1_genomic	2914470	China: Beijing	2018
GCA_015946525.1_PDT000670929.1_genomic	2715544	China: Beijing	2017
GCA_015946545.1_PDT000670919.1_genomic	2753984	China: Beijing	2018
GCA_015946565.1_PDT000670926.1_genomic	2912757	China: Beijing	2018
GCA_015946585.1_PDT000670927.1_genomic	2776081	China: Beijing	2017
GCA_015946595.1_PDT000670925.1_genomic	2799177	China: Beijing	2017
GCA_015946625.1_PDT000670913.1_genomic	2819745	China: Beijing	2018

GCA_015946645.1_PDT000670932.1_genomic	2758493	China: Beijing	2017
GCA_015946665.1_PDT000670928.1_genomic	2909180	China: Beijing	2018
GCA_015946685.1_PDT000670915.1_genomic	2649472	China: Beijing	2018
GCA_015946705.1_PDT000670909.1_genomic	2914067	China: Beijing	2018
GCA_015946725.1_PDT000670914.1_genomic	2913548	China: Beijing	2018
GCA_015946745.1_PDT000670924.1_genomic	2776552	China: Beijing	2017
GCA_015946765.1_PDT000670911.1_genomic	2882915	China: Beijing	2018
GCA_015946775.1_PDT000670930.1_genomic	2814845	China: Beijing	2017
GCA_015946805.1_PDT000670902.1_genomic	2840938	China: Beijing	2018
GCA_015946825.1_PDT000670908.1_genomic	2755615	China: Beijing	2018
GCA_015946845.1_PDT000670936.1_genomic	2756966	China: Beijing	2017
GCA_015946865.1_PDT000670907.1_genomic	2741396	China: Beijing	2018
GCA_015946885.1_PDT000670903.1_genomic	2791263	China: Beijing	2018
GCA_015946905.1_PDT000670937.1_genomic	2787011	China: Beijing	2017
GCA_015946925.1_PDT000670904.1_genomic	2757835	China: Beijing	2018
GCA_015946945.1_PDT000670935.1_genomic	2948530	China: Beijing	2017
GCA_015946965.1_PDT000670922.1_genomic	2766861	China: Beijing	2018
GCA_015946985.1_PDT000670921.1_genomic	2789838	China: Beijing	2018
GCA_015947005.1_PDT000670918.1_genomic	2833407	China: Beijing	2018
GCA_015947025.1_PDT000670923.1_genomic	2871755	China: Beijing	2018
GCA_015947045.1_PDT000670920.1_genomic	2789246	China: Beijing	2018
015947065.1_PDT000670912.1_genomic	2791529	China: Beijing	2018
GCA_015947085.1_PDT000670901.1_genomic	2913436	China: Beijing	2018
GCA_015947105.1_PDT000670905.1_genomic	2912844	China: Beijing	2018
GCA_015947125.1_PDT000670900.1_genomic	2751507	China: Beijing	2018
GCA_015947945.1_PDT000670906.1_genomic	2765968	China: Beijing	2018
GCA_016084495.1_PDT000200506.2_genomic	2879396	USA:Boston	2017
GCA_016084535.1_PDT000173735.3_genomic	2857574	USA:Boston	2016
GCA_016084555.1_PDT000172048.3_genomic	2830048	USA:Boston	2016
GCA_016085355.1_PDT000173738.2_genomic	2893255	USA:Boston	2016
GCA_016088575.1_PDT000133403.2_genomic	2906301	USA:Boston	2016
GCA_016109815.1_PDT000902817.1_genomic	2792986	China: Beijing	2016
GCA_016403285.1_ASM1640328v1_genomic	2952500	Germany: Braunschweig	1990
GCA_016728105.1_ASM1672810v1_genomic	2904831	Germany: Braunschweig	1992
GCA_016728205.1_ASM1672820v1_genomic	2665682	Germany: Braunschweig	1992
GCA_016889445.1_ASM1688944v1_genomic	3004754	Germany: Braunschweig	2011
GCA_018144015.1_PDT001014179.1_genomic	2746065	Denmark	2018
GCA_018164715.1_PDT001013020.1_genomic	2773146	Denmark	2018
GCA_018178195.1_PDT001013023.1_genomic	2718786	Denmark	2018
GCA_900447625.1_55064_G01_genomic	2784006	UK	2018
GCA_900447675.1_51765_E02_genomic	2924414	UK	2018

GCA_900683645.1_57792_F02_genomic	2916518	UK	2019
GCF_000988225.1_ASM98822v1_genomic	2611976	Brazil	2009
GCF_001053405.1_ASM105340v1_genomic	2914153	USA: WA	2012
GCF_001053435.1_ASM105343v1_genomic	2905202	USA: WA	2012
GCF_001055405.1_ASM105540v1_genomic	2784991	USA: WA	2012
GCF_001058455.1_ASM105845v1_genomic	2743259	USA: WA	2012
GCF_001059665.1_ASM105966v1_genomic	2834694	USA: WA	2012
GCF_001059955.1_ASM105995v1_genomic	2830184	USA: WA	2012
GCF_001076975.1_ASM107697v1_genomic	2829078	USA: WA	2012
GCF_002156805.1_ASM215680v1_genomic	2797692	South Korea: South sea	2017
GCF_002775055.1_ASM277505v1_genomic	2898882	Brazil: Rio de Janeiro	2011
GCF_002775095.1_ASM277509v1_genomic	2375104	Brazil: Rio de Janeiro	2009
GCF_002775105.1_ASM277510v1_genomic	2875402	Brazil: Rio de Janeiro	2011
GCF_002803965.1_ASM280396v1_genomic	2993983	USA: Boston	2016
GCF_002804085.1_ASM280408v1_genomic	3031488	USA: Boston	2016
GCF_002865925.1_ASM286592v1_genomic	2820414	Brazil: teaching hospital in Rio de Janeiro	2009
GCF_003202245.1_ASM320224v1_genomic	3052429	Brazil: Rio de Janeiro	2010
GCF_003202295.1_ASM320229v1_genomic	2861961	Brazil: Rio de Janeiro	2011
GCF_003202335.1_ASM320233v1_genomic	3003571	Brazil: Rio de Janeiro	2011
GCF_003202355.1_ASM320235v1_genomic	2847990	Brazil: Rio de Janeiro	2012
GCF_003202375.1_ASM320237v1_genomic	3128097	Brazil: Rio de Janeiro	2011
GCF_004138065.1_ASM413806v1_genomic	2824576	USA: Missouri	2013
GCF_006538485.1_ASM653848v1_genomic	3113921	Japan	2019
GCF_008373895.1_ASM837389v1_genomic	2824107	USA	2011
GCF_011682935.1_ASM1168293v1_genomic	2981342	China: Beijing	2018
GCF_011682945.1_ASM1168294v1_genomic	2937252	China: Beijing	2018
GCF_011682965.1_ASM1168296v1_genomic	2796099	China: Beijing	2020
GCF_011682985.1_ASM1168298v1_genomic	2788964	China: Beijing	2020
GCF_014610855.1_ASM1461085v1_genomic	2840792	Surgical wound secretion	2020
GCF_014610885.1_ASM1461088v1_genomic	2841405	Brazil: Rio de Janeiro	2009
GCF_016403285.1_ASM1640328v1_genomic	2952500	Germany: Braunschweig	2020
GCF_016728105.1_ASM1672810v1_genomic	2904831	Germany: Braunschweig	2021
GCF_016728205.1_ASM1672820v1_genomic	2665682	Germany: Braunschweig	2021
GCF_016889445.1_ASM1688944v1_genomic	3004754	Germany: Braunschweig	2021
GCF_900447625.1_55064_G01_genomic	2784006	UK	2018
GCF_900447675.1_51765_E02_genomic	2924414	UK	2018
GCF_900683645.1_57792_F02_genomic	2916518	UK	2019

Supplementary Table 2: Integrons predictions in analyzed *C. striatum* genomes

Strain ID	element	Start	End	Description	Annotation	Type
137	RAQW01000005.1_144	142685	143698	protein	intI	complete
	P_intI1	143718	143752	Promoter	Pint_1	complete
	RAQW01000005.1_145	143799	144428	protein	AAC_6p_Ib-NCBIFAM	complete
	attc_001	144423	144494	attC	attC	complete
	RAQW01000005.1_146	144510	145286	protein	ANT_3pp_AadA1-NCBIFAM	complete
	attc_002	145288	145347	attC	attC	complete
	RAQW01000005.1_147	145450	145797	protein	SMR_qac_E-NCBIFAM	complete
140	VCOZ01000051.1_2	912	1703	protein	intI	complete
	P_intI1	1723	1757	Promoter	Pint_1	complete
	attI1	1782	1840	attI	attI_1	complete
	VCOZ01000051.1_3	1883	2440	protein	AAC_6p_Ia_fam-NCBIFAM	complete
	VCOZ01000051.1_4	2445	2870	protein	protein	complete
	VCOZ01000051.1_5	2922	3797	protein	ANT_3pp_AadA1-NCBIFAM	complete
	attc_001	3799	3858	attC	attC	complete
	VCOZ01000051.1_6	3974	4090	protein	protein	complete
141	VCOY01000046.1_1	3	230	protein	protein	complete
	attc_001	346	405	attC	attC	complete
	VCOY01000046.1_2	407	1282	protein	ANT_3pp_AadA1-NCBIFAM	complete
	VCOY01000046.1_3	1334	1759	protein	protein	complete
	VCOY01000046.1_4	1764	2321	protein	AAC_6p_Ia_fam-NCBIFAM	complete
	P_intI1	2446	2480	Promoter	Pint_1	complete
	VCOY01000046.1_5	2501	3292	protein	intI	complete
145	DACRXP010000058.1_3	1542	1889	protein	SMR_qac_E-NCBIFAM	complete
	attc_001	1992	2063	attC	attC	complete
	DACRXP010000058.1_4	2058	2687	protein	AAC_6p_Ib-NCBIFAM	complete
	P_intI1	2800	2834	Promoter	Pint_1	complete
	DACRXP010000058.1_5	2855	3607	protein	intI	complete
	DACRXP010000124.1_1	5	796	protein	intI	complete
	DACRXP010000124.1_2	964	1593	protein	AAC_6p_Ib-NCBIFAM	complete
	attc_001	1588	1659	attC	attC	complete
	DACRXP010000124.1_3	1762	2109	protein	SMR_qac_E-NCBIFAM	complete
146	DACRXQ010000044.1_1	3	122	protein	protein	complete
	attc_001	238	297	attC	attC	complete
	DACRXQ010000044.1_2	299	1174	protein	ANT_3pp_AadA1-NCBIFAM	complete
	DACRXQ010000044.1_3	1226	1651	protein	protein	complete
	DACRXQ010000044.1_4	1656	2213	protein	AAC_6p_Ia_fam-NCBIFAM	complete
	P_intI1	2338	2372	Promoter	Pint_1	complete

	DACRXQ010000044.1_5	2393	3184	protein	intI	complete
	DACRXQ010000078.1_2	825	1616	protein	intI	complete
	P_intI1	1636	1670	Promoter	Pint_1	complete
	attI1	1695	1753	attI	attI_1	complete
	DACRXQ010000078.1_3	1796	2353	protein	AAC_6p_Ia_fam-NCBIFAM	complete
	DACRXQ010000078.1_4	2358	2783	protein	protein	complete
	DACRXQ010000078.1_5	2835	3710	protein	ANT_3pp_AadA1-NCBIFAM	complete
	attc_001	3712	3771	attC	attC	complete
279	DACRYF010000061.1_1	3	122	protein	protein	complete
	attc_001	238	297	attC	attC	complete
	DACRYF010000061.1_2	299	1174	protein	ANT_3pp_AadA1-NCBIFAM	complete
	DACRYF010000061.1_3	1226	1651	protein	protein	complete
	DACRYF010000061.1_4	1656	2213	protein	AAC_6p_Ia_fam-NCBIFAM	complete
	P_intI1	2338	2372	Promoter	Pint_1	complete
	DACRYF010000061.1_5	2393	3184	protein	intI	complete
	DACRYF010000103.1_2	825	1616	protein	intI	complete
	P_intI1	1636	1670	Promoter	Pint_1	complete
	attI1	1695	1753	attI	attI_1	complete
	DACRYF010000103.1_3	1796	2353	protein	AAC_6p_Ia_fam-NCBIFAM	complete
	DACRYF010000103.1_4	2358	2783	protein	protein	complete
	DACRYF010000103.1_5	2835	3710	protein	ANT_3pp_AadA1-NCBIFAM	complete
	attc_001	3712	3771	attC	attC	complete
282	DACRYB010000056.1_3	1542	1889	protein	SMR_qac_E-NCBIFAM	complete
	attc_001	1992	2063	attC	attC	complete
	DACRYB010000056.1_4	2058	2687	protein	AAC_6p_Ib-NCBIFAM	complete
	P_intI1	2800	2834	Promoter	Pint_1	complete
	DACRYB010000056.1_5	2855	3607	protein	intI	complete
	DACRYB010000118.1_1	5	796	protein	intI	complete
	DACRYB010000118.1_2	964	1593	protein	AAC_6p_Ib-NCBIFAM	complete
	attc_001	1588	1659	attC	attC	complete
	DACRYB010000118.1_3	1762	2109	protein	SMR_qac_E-NCBIFAM	complete
286	DACRYC010000063.1_3	1542	1889	protein	SMR_qac_E-NCBIFAM	complete
	attc_001	1992	2063	attC	attC	complete
	DACRYC010000063.1_4	2058	2687	protein	AAC_6p_Ib-NCBIFAM	complete
	P_intI1	2800	2834	Promoter	Pint_1	complete
	DACRYC010000063.1_5	2855	3607	protein	intI	complete
	DACRYC010000127.1_1	5	796	protein	intI	complete
	DACRYC010000127.1_2	964	1593	protein	AAC_6p_Ib-NCBIFAM	complete
	attc_001	1588	1659	attC	attC	complete
	DACRYC010000127.1_3	1762	2109	protein	SMR_qac_E-NCBIFAM	complete
289	DACRXO010000053.1_3	1542	1889	protein	SMR_qac_E-NCBIFAM	complete

	attc_001	1992	2063	attC	attC	complete
	DACRXO010000053.1_4	2058	2687	protein	AAC_6p_Ib-NCBIFAM	complete
	attI1	2717	2775	attI	attI_1	complete
	P_intI1	2800	2834	Promoter	Pint_1	complete
	DACRXO010000053.1_5	2855	3646	protein	intI	complete
	DACRXO010000140.1_2	825	1616	protein	intI	complete
	P_intI1	1636	1670	Promoter	Pint_1	complete
	attI1	1695	1753	attI	attI_1	complete
	DACRXO010000140.1_3	1784	2413	protein	AAC_6p_Ib-NCBIFAM	complete
	attc_001	2408	2479	attC	attC	complete
	DACRXO010000140.1_4	2582	2929	protein	SMR_qac_E-NCBIFAM	complete
290	DACRXR010000045.1_1	3	122	protein	protein	complete
	attc_001	238	297	attC	attC	complete
	DACRXR010000045.1_2	299	1174	protein	ANT_3pp_AadA1-NCBIFAM	complete
	DACRXR010000045.1_3	1226	1651	protein	protein	complete
	DACRXR010000045.1_4	1656	2213	protein	AAC_6p_Ia_fam-NCBIFAM	complete
	P_intI1	2338	2372	Promoter	Pint_1	complete
	DACRXR010000045.1_5	2393	3184	protein	intI	complete
	DACRXR010000085.1_2	825	1616	protein	intI	complete
	P_intI1	1636	1670	Promoter	Pint_1	complete
	attI1	1695	1753	attI	attI_1	complete
	DACRXR010000085.1_3	1796	2353	protein	AAC_6p_Ia_fam-NCBIFAM	complete
	DACRXR010000085.1_4	2358	2783	protein	protein	complete
	DACRXR010000085.1_5	2835	3710	protein	ANT_3pp_AadA1-NCBIFAM	complete
	attc_001	3712	3771	attC	attC	complete
291	DACRXS010000045.1_1	3	122	protein	protein	complete
	attc_001	238	297	attC	attC	complete
	DACRXS010000045.1_2	299	1174	protein	ANT_3pp_AadA1-NCBIFAM	complete
	DACRXS010000045.1_3	1226	1651	protein	protein	complete
	DACRXS010000045.1_4	1656	2213	protein	AAC_6p_Ia_fam-NCBIFAM	complete
	P_intI1	2338	2372	Promoter	Pint_1	complete
	DACRXS010000045.1_5	2393	3184	protein	intI	complete
	DACRXS010000082.1_2	825	1616	protein	intI	complete
	P_intI1	1636	1670	Promoter	Pint_1	complete
	attI1	1695	1753	attI	attI_1	complete
	DACRXS010000082.1_3	1796	2353	protein	AAC_6p_Ia_fam-NCBIFAM	complete
	DACRXS010000082.1_4	2358	2783	protein	protein	complete
	DACRXS010000082.1_5	2835	3710	protein	ANT_3pp_AadA1-NCBIFAM	complete
	attc_001	3712	3771	attC	attC	complete
292	DACRYZ010000051.1_1	3	122	protein	protein	complete
	attc_001	238	297	attC	attC	complete

	DACRYZ010000051.1_2	299	1174	protein	ANT_3pp_AadA1-NCBIFAM	complete
	DACRYZ010000051.1_3	1226	1651	protein	protein	complete
	DACRYZ010000051.1_4	1656	2213	protein	AAC_6p_Ia_fam-NCBIFAM	complete
	P_intI1	2338	2372	Promoter	Pint_1	complete
	DACRYZ010000051.1_5	2393	3184	protein	intI	complete
	DACRYZ010000091.1_2	825	1616	protein	intI	complete
	P_intI1	1636	1670	Promoter	Pint_1	complete
	attI1	1695	1753	attI	attI_1	complete
	DACRYZ010000091.1_3	1796	2353	protein	AAC_6p_Ia_fam-NCBIFAM	complete
	DACRYZ010000091.1_4	2358	2783	protein	protein	complete
	attc_001	2798	2844	attC	attC	complete
293	attc_001	46	105	attC	attC	complete
	DACRYY010000052.1_1	107	982	protein	ANT_3pp_AadA1-NCBIFAM	complete
	DACRYY010000052.1_2	1034	1459	protein	protein	complete
	DACRYY010000052.1_3	1464	2021	protein	AAC_6p_Ia_fam-NCBIFAM	complete
	P_intI1	2146	2180	Promoter	Pint_1	complete
	DACRYY010000052.1_4	2201	2992	protein	intI	complete
	DACRYY010000091.1_2	825	1616	protein	intI	complete
	P_intI1	1636	1670	Promoter	Pint_1	complete
	attI1	1695	1753	attI	attI_1	complete
	DACRYY010000091.1_3	1796	2353	protein	AAC_6p_Ia_fam-NCBIFAM	complete
	DACRYY010000091.1_4	2358	2783	protein	protein	complete
	attc_001	2798	2844	attC	attC	complete
294	DACRYW010000053.1_1	3	122	protein	protein	complete
	attc_001	238	297	attC	attC	complete
	DACRYW010000053.1_2	299	1174	protein	ANT_3pp_AadA1-NCBIFAM	complete
	DACRYW010000053.1_3	1226	1651	protein	protein	complete
	DACRYW010000053.1_4	1656	2213	protein	AAC_6p_Ia_fam-NCBIFAM	complete
	P_intI1	2338	2372	Promoter	Pint_1	complete
	DACRYW010000053.1_5	2393	3184	protein	intI	complete
	DACRYW010000092.1_2	825	1616	protein	intI	complete
	P_intI1	1636	1670	Promoter	Pint_1	complete
	attI1	1695	1753	attI	attI_1	complete
	DACRYW010000092.1_3	1796	2353	protein	AAC_6p_Ia_fam-NCBIFAM	complete
	DACRYW010000092.1_4	2358	2783	protein	protein	complete
	DACRYW010000092.1_5	2835	3710	protein	ANT_3pp_AadA1-NCBIFAM	complete
	attc_001	3712	3771	attC	attC	complete
296	DACRYT010000052.1_3	1542	1889	protein	SMR_qac_E-NCBIFAM	complete
	attc_001	1992	2063	attC	attC	complete
	DACRYT010000052.1_4	2058	2687	protein	AAC_6p_Ib-NCBIFAM	complete
	attI1	2717	2775	attI	attI_1	complete

	P_intI1	2800	2834	Promoter	Pint_1	complete
	DACRYT010000052.1_5	2855	3646	protein	intI	complete
	DACRYT010000112.1_2	825	1616	protein	intI	complete
	P_intI1	1636	1670	Promoter	Pint_1	complete
	attI1	1695	1753	attI	attI_1	complete
	DACRYT010000112.1_3	1784	2413	protein	AAC_6p_Ib-NCBIFAM	complete
	attc_001	2408	2479	attC	attC	complete
	DACRYT010000112.1_4	2582	2929	protein	SMR_qac_E-NCBIFAM	complete
298	DACRXT010000058.1_1	3	122	protein	protein	complete
	attc_001	238	297	attC	attC	complete
	DACRXT010000058.1_2	299	1174	protein	ANT_3pp_AadA1-NCBIFAM	complete
	DACRXT010000058.1_3	1226	1651	protein	protein	complete
	DACRXT010000058.1_4	1656	2213	protein	AAC_6p_Ia_fam-NCBIFAM	complete
	P_intI1	2338	2372	Promoter	Pint_1	complete
	DACRXT010000058.1_5	2393	3184	protein	intI	complete
	DACRXT010000106.1_2	825	1616	protein	intI	complete
	P_intI1	1636	1670	Promoter	Pint_1	complete
	attI1	1695	1753	attI	attI_1	complete
	DACRXT010000106.1_3	1796	2353	protein	AAC_6p_Ia_fam-NCBIFAM	complete
	DACRXT010000106.1_4	2358	2783	protein	protein	complete
	DACRXT010000106.1_5	2835	3710	protein	ANT_3pp_AadA1-NCBIFAM	complete
	attc_001	3712	3771	attC	attC	complete
300	DACRYX010000047.1_1	3	122	protein	protein	complete
	attc_001	238	297	attC	attC	complete
	DACRYX010000047.1_2	299	1174	protein	ANT_3pp_AadA1-NCBIFAM	complete
	DACRYX010000047.1_3	1226	1651	protein	protein	complete
	DACRYX010000047.1_4	1656	2213	protein	AAC_6p_Ia_fam-NCBIFAM	complete
	P_intI1	2338	2372	Promoter	Pint_1	complete
	DACRYX010000047.1_5	2393	3184	protein	intI	complete
	DACRYX010000084.1_2	825	1616	protein	intI	complete
	P_intI1	1636	1670	Promoter	Pint_1	complete
	attI1	1695	1753	attI	attI_1	complete
	DACRYX010000084.1_3	1796	2353	protein	AAC_6p_Ia_fam-NCBIFAM	complete
	DACRYX010000084.1_4	2358	2783	protein	protein	complete
	DACRYX010000084.1_5	2835	3710	protein	ANT_3pp_AadA1-NCBIFAM	complete
	attc_001	3712	3771	attC	attC	complete
301	DACRYV010000059.1_3	1542	1889	protein	SMR_qac_E-NCBIFAM	complete
	attc_001	1992	2063	attC	attC	complete
	DACRYV010000059.1_4	2058	2687	protein	AAC_6p_Ib-NCBIFAM	complete
	P_intI1	2800	2834	Promoter	Pint_1	complete
	DACRYV010000059.1_5	2855	3607	protein	intI	complete

	DACRYV010000131.1_1	5	796	protein	intI	complete
	DACRYV010000131.1_2	964	1593	protein	AAC_6p_Ib-NCBIFAM	complete
	attc_001	1588	1659	attC	attC	complete
	DACRYV010000131.1_3	1762	2109	protein	SMR_qac_E-NCBIFAM	complete
302	DACRXW010000046.1_1	3	122	protein	protein	complete
	attc_001	238	297	attC	attC	complete
	DACRXW010000046.1_2	299	1174	protein	ANT_3pp_AadA1-NCBIFAM	complete
	DACRXW010000046.1_3	1226	1651	protein	protein	complete
	DACRXW010000046.1_4	1656	2213	protein	AAC_6p_Ia_fam-NCBIFAM	complete
	P_intI1	2338	2372	Promoter	Pint_1	complete
	DACRXW010000046.1_5	2393	3184	protein	intI	complete
	DACRXW010000084.1_2	825	1616	protein	intI	complete
	P_intI1	1636	1670	Promoter	Pint_1	complete
	attI1	1695	1753	attI	attI_1	complete
	DACRXW010000084.1_3	1796	2353	protein	AAC_6p_Ia_fam-NCBIFAM	complete
	DACRXW010000084.1_4	2358	2783	protein	protein	complete
	attc_001	2798	2844	attC	attC	complete
303	DACRXU010000050.1_1	3	122	protein	protein	complete
	attc_001	238	297	attC	attC	complete
	DACRXU010000050.1_2	299	1174	protein	ANT_3pp_AadA1-NCBIFAM	complete
	DACRXU010000050.1_3	1226	1651	protein	protein	complete
	DACRXU010000050.1_4	1656	2213	protein	AAC_6p_Ia_fam-NCBIFAM	complete
	P_intI1	2338	2372	Promoter	Pint_1	complete
	DACRXU010000050.1_5	2393	3184	protein	intI	complete
	DACRXU010000081.1_2	825	1616	protein	intI	complete
	P_intI1	1636	1670	Promoter	Pint_1	complete
	attI1	1695	1753	attI	attI_1	complete
	DACRXU010000081.1_3	1796	2353	protein	AAC_6p_Ia_fam-NCBIFAM	complete
	DACRXU010000081.1_4	2358	2783	protein	protein	complete
	DACRXU010000081.1_5	2835	3710	protein	ANT_3pp_AadA1-NCBIFAM	complete
	attc_001	3712	3771	attC	attC	complete
304	DACRZC010000052.1_1	3	83	protein	protein	complete
	attc_001	199	258	attC	attC	complete
	DACRZC010000052.1_2	260	1135	protein	ANT_3pp_AadA1-NCBIFAM	complete
	DACRZC010000052.1_3	1187	1612	protein	protein	complete
	DACRZC010000052.1_4	1617	2174	protein	AAC_6p_Ia_fam-NCBIFAM	complete
	P_intI1	2299	2333	Promoter	Pint_1	complete
	DACRZC010000052.1_5	2354	3145	protein	intI	complete
	DACRZC010000092.1_2	825	1616	protein	intI	complete
	P_intI1	1636	1670	Promoter	Pint_1	complete
	attI1	1695	1753	attI	attI_1	complete

	DACRZC010000092.1_3	1796	2353	protein	AAC_6p_Ia_fam-NCBIFAM	complete
	DACRZC010000092.1_4	2358	2783	protein	protein	complete
	DACRZC010000092.1_5	2835	3710	protein	ANT_3pp_AadA1-NCBIFAM	complete
	attc_001	3712	3771	attC	attC	complete
305	attc_001	46	105	attC	attC	complete
	DACRZA010000053.1_1	107	982	protein	ANT_3pp_AadA1-NCBIFAM	complete
	DACRZA010000053.1_2	1034	1459	protein	protein	complete
	DACRZA010000053.1_3	1464	2021	protein	AAC_6p_Ia_fam-NCBIFAM	complete
	P_intI1	2146	2180	Promoter	Pint_1	complete
	DACRZA010000053.1_4	2201	2992	protein	intI	complete
	DACRZA010000084.1_2	825	1616	protein	intI	complete
	P_intI1	1636	1670	Promoter	Pint_1	complete
	attI1	1695	1753	attI	attI_1	complete
	DACRZA010000084.1_3	1796	2353	protein	AAC_6p_Ia_fam-NCBIFAM	complete
	DACRZA010000084.1_4	2358	2783	protein	protein	complete
	DACRZA010000084.1_5	2835	3710	protein	ANT_3pp_AadA1-NCBIFAM	complete
	attc_001	3712	3771	attC	attC	complete
308	DACRYU010000051.1_1	3	122	protein	protein	complete
	attc_001	238	297	attC	attC	complete
	DACRYU010000051.1_2	299	1174	protein	ANT_3pp_AadA1-NCBIFAM	complete
	DACRYU010000051.1_3	1226	1651	protein	protein	complete
	DACRYU010000051.1_4	1656	2213	protein	AAC_6p_Ia_fam-NCBIFAM	complete
	P_intI1	2338	2372	Promoter	Pint_1	complete
	DACRYU010000051.1_5	2393	3184	protein	intI	complete
	DACRYU010000094.1_2	825	1616	protein	intI	complete
	P_intI1	1636	1670	Promoter	Pint_1	complete
	attI1	1695	1753	attI	attI_1	complete
	DACRYU010000094.1_3	1796	2353	protein	AAC_6p_Ia_fam-NCBIFAM	complete
	DACRYU010000094.1_4	2358	2783	protein	protein	complete
	DACRYU010000094.1_5	2835	3710	protein	ANT_3pp_AadA1-NCBIFAM	complete
	attc_001	3712	3771	attC	attC	complete
309	DACRYI010000047.1_1	3	122	protein	protein	complete
	attc_001	238	297	attC	attC	complete
	DACRYI010000047.1_2	299	1174	protein	ANT_3pp_AadA1-NCBIFAM	complete
	DACRYI010000047.1_3	1226	1651	protein	protein	complete
	DACRYI010000047.1_4	1656	2213	protein	AAC_6p_Ia_fam-NCBIFAM	complete
	P_intI1	2338	2372	Promoter	Pint_1	complete
	DACRYI010000047.1_5	2393	3184	protein	intI	complete
	DACRYI010000082.1_2	825	1616	protein	intI	complete
	P_intI1	1636	1670	Promoter	Pint_1	complete
	attI1	1695	1753	attI	attI_1	complete

	DACRYI010000082.1_3	1796	2353	protein	AAC_6p_Ia_fam-NCBIFAM	complete
	DACRYI010000082.1_4	2358	2783	protein	protein	complete
	attc_001	2798	2844	attC	attC	complete
469	DACSAW010000054.1_1	106	981	protein	ANT_3pp_AadA1-NCBIFAM	complete
	DACSAW010000054.1_2	1033	1458	protein	protein	complete
	DACSAW010000054.1_3	1463	2020	protein	AAC_6p_Ia_fam-NCBIFAM	complete
	P_intI1	2145	2179	Promoter	Pint_1	complete
	DACSAW010000054.1_4	2200	2991	protein	intI	complete
	DACSAW010000089.1_2	825	1616	protein	intI	complete
	P_intI1	1636	1670	Promoter	Pint_1	complete
	attI1	1695	1753	attI	attI_1	complete
	DACSAW010000089.1_3	1796	2353	protein	AAC_6p_Ia_fam-NCBIFAM	complete
	DACSAW010000089.1_4	2358	2783	protein	protein	complete
	DACSAW010000089.1_5	2835	3710	protein	ANT_3pp_AadA1-NCBIFAM	complete
	attc_001	3712	3771	attC	attC	complete
470	DACRYK010000049.1_1	3	122	protein	protein	complete
	attc_001	238	297	attC	attC	complete
	DACRYK010000049.1_2	299	1174	protein	ANT_3pp_AadA1-NCBIFAM	complete
	DACRYK010000049.1_3	1226	1651	protein	protein	complete
	DACRYK010000049.1_4	1656	2213	protein	AAC_6p_Ia_fam-NCBIFAM	complete
	P_intI1	2338	2372	Promoter	Pint_1	complete
	DACRYK010000049.1_5	2393	3184	protein	intI	complete
	DACRYK010000091.1_2	825	1616	protein	intI	complete
	P_intI1	1636	1670	Promoter	Pint_1	complete
	attI1	1695	1753	attI	attI_1	complete
	DACRYK010000091.1_3	1796	2353	protein	AAC_6p_Ia_fam-NCBIFAM	complete
	DACRYK010000091.1_4	2358	2783	protein	protein	complete
	DACRYK010000091.1_5	2835	3710	protein	ANT_3pp_AadA1-NCBIFAM	complete
	attc_001	3712	3771	attC	attC	complete
471	DACRYJ010000047.1_1	3	122	protein	protein	complete
	attc_001	238	297	attC	attC	complete
	DACRYJ010000047.1_2	299	1174	protein	ANT_3pp_AadA1-NCBIFAM	complete
	DACRYJ010000047.1_3	1226	1651	protein	protein	complete
	DACRYJ010000047.1_4	1656	2213	protein	AAC_6p_Ia_fam-NCBIFAM	complete
	P_intI1	2338	2372	Promoter	Pint_1	complete
	DACRYJ010000047.1_5	2393	3184	protein	intI	complete
	DACRYJ010000086.1_2	825	1616	protein	intI	complete
	P_intI1	1636	1670	Promoter	Pint_1	complete
	attI1	1695	1753	attI	attI_1	complete
	DACRYJ010000086.1_3	1796	2353	protein	AAC_6p_Ia_fam-NCBIFAM	complete
	DACRYJ010000086.1_4	2358	2783	protein	protein	complete

	DACRYJ010000086.1_5	2835	3710	protein	ANT_3pp_AadA1-NCBIFAM	complete
	attc_001	3712	3771	attC	attC	complete
472	DACRYL010000051.1_1	3	122	protein	protein	complete
	attc_001	238	297	attC	attC	complete
	DACRYL010000051.1_2	299	1174	protein	ANT_3pp_AadA1-NCBIFAM	complete
	DACRYL010000051.1_3	1226	1651	protein	protein	complete
	DACRYL010000051.1_4	1656	2213	protein	AAC_6p_Ia_fam-NCBIFAM	complete
	P_intI1	2338	2372	Promoter	Pint_1	complete
	DACRYL010000051.1_5	2393	3184	protein	intI	complete
	DACRYL010000086.1_2	825	1616	protein	intI	complete
	P_intI1	1636	1670	Promoter	Pint_1	complete
	attI1	1695	1753	attI	attI_1	complete
	DACRYL010000086.1_3	1796	2353	protein	AAC_6p_Ia_fam-NCBIFAM	complete
	DACRYL010000086.1_4	2358	2783	protein	protein	complete
	DACRYL010000086.1_5	2835	3710	protein	ANT_3pp_AadA1-NCBIFAM	complete
	attc_001	3712	3771	attC	attC	complete
473	DACRYN010000067.1_3	1542	1889	protein	SMR_qac_E-NCBIFAM	complete
	attc_001	1992	2063	attC	attC	complete
	DACRYN010000067.1_4	2058	2687	protein	AAC_6p_Ib-NCBIFAM	complete
	P_intI1	2800	2834	Promoter	Pint_1	complete
	DACRYN010000067.1_5	2855	3607	protein	intI	complete
	DACRYN010000136.1_1	5	796	protein	intI	complete
	DACRYN010000136.1_2	964	1593	protein	AAC_6p_Ib-NCBIFAM	complete
	attc_001	1588	1659	attC	attC	complete
	DACRYN010000136.1_3	1762	2109	protein	SMR_qac_E-NCBIFAM	complete
474	DACRYM010000058.1_3	1542	1889	protein	SMR_qac_E-NCBIFAM	complete
	attc_001	1992	2063	attC	attC	complete
	DACRYM010000058.1_4	2058	2687	protein	AAC_6p_Ib-NCBIFAM	complete
	attI1	2717	2775	attI	attI_1	complete
	P_intI1	2800	2834	Promoter	Pint_1	complete
	DACRYM010000058.1_5	2855	3646	protein	intI	complete
	DACRYM010000137.1_2	825	1616	protein	intI	complete
	P_intI1	1636	1670	Promoter	Pint_1	complete
	attI1	1695	1753	attI	attI_1	complete
	DACRYM010000137.1_3	1784	2413	protein	AAC_6p_Ib-NCBIFAM	complete
	attc_001	2408	2479	attC	attC	complete
	DACRYM010000137.1_4	2582	2929	protein	SMR_qac_E-NCBIFAM	complete
476	DACRYO010000065.1_3	1542	1889	protein	SMR_qac_E-NCBIFAM	complete
	attc_001	1992	2063	attC	attC	complete
	DACRYO010000065.1_4	2058	2687	protein	AAC_6p_Ib-NCBIFAM	complete
	P_intI1	2800	2834	Promoter	Pint_1	complete

	DACRYO010000065.1_5	2855	3607	protein	intI	complete
	DACRYO010000127.1_1	5	796	protein	intI	complete
	DACRYO010000127.1_2	964	1593	protein	AAC_6p_Ib-NCBIFAM	complete
	attc_001	1588	1659	attC	attC	complete
	DACRYO010000127.1_3	1762	2109	protein	SMR_qac_E-NCBIFAM	complete
478	DACRYR010000060.1_3	1542	1889	protein	SMR_qac_E-NCBIFAM	complete
	attc_001	1992	2063	attC	attC	complete
	DACRYR010000060.1_4	2058	2687	protein	AAC_6p_Ib-NCBIFAM	complete
	P_intI1	2800	2834	Promoter	Pint_1	complete
	DACRYR010000060.1_5	2855	3607	protein	intI	complete
	DACRYR010000120.1_1	5	796	protein	intI	complete
	DACRYR010000120.1_2	964	1593	protein	AAC_6p_Ib-NCBIFAM	complete
	attc_001	1588	1659	attC	attC	complete
	DACRYR010000120.1_3	1762	2109	protein	SMR_qac_E-NCBIFAM	complete
479	DACRYP010000065.1_3	1542	1889	protein	SMR_qac_E-NCBIFAM	complete
	attc_001	1992	2063	attC	attC	complete
	DACRYP010000065.1_4	2058	2687	protein	AAC_6p_Ib-NCBIFAM	complete
	P_intI1	2800	2834	Promoter	Pint_1	complete
	DACRYP010000065.1_5	2855	3607	protein	intI	complete
	DACRYP010000123.1_1	5	796	protein	intI	complete
	DACRYP010000123.1_2	964	1593	protein	AAC_6p_Ib-NCBIFAM	complete
	attc_001	1588	1659	attC	attC	complete
	DACRYP010000123.1_3	1762	2109	protein	SMR_qac_E-NCBIFAM	complete
481	DACRZG010000055.1_3	1542	1889	protein	SMR_qac_E-NCBIFAM	complete
	attc_001	1992	2063	attC	attC	complete
	DACRZG010000055.1_4	2058	2687	protein	AAC_6p_Ib-NCBIFAM	complete
	attI1	2717	2775	attI	attI_1	complete
	P_intI1	2800	2834	Promoter	Pint_1	complete
	DACRZG010000055.1_5	2855	3646	protein	intI	complete
	DACRZG010000134.1_2	825	1616	protein	intI	complete
	P_intI1	1636	1670	Promoter	Pint_1	complete
	attI1	1695	1753	attI	attI_1	complete
	DACRZG010000134.1_3	1784	2413	protein	AAC_6p_Ib-NCBIFAM	complete
	attc_001	2408	2479	attC	attC	complete
	DACRZG010000134.1_4	2582	2929	protein	SMR_qac_E-NCBIFAM	complete
484	DACRZH010000051.1_1	3	122	protein	protein	complete
	attc_001	238	297	attC	attC	complete
	DACRZH010000051.1_2	299	1174	protein	ANT_3pp_AadA1-NCBIFAM	complete
	DACRZH010000051.1_3	1226	1651	protein	protein	complete
	DACRZH010000051.1_4	1656	2213	protein	AAC_6p_Ia_fam-NCBIFAM	complete
	P_intI1	2338	2372	Promoter	Pint_1	complete

	DACRZH010000051.1_5	2393	3184	protein	intI	complete
	DACRZH010000085.1_2	825	1616	protein	intI	complete
	P_intI1	1636	1670	Promoter	Pint_1	complete
	attI1	1695	1753	attI	attI_1	complete
	DACRZH010000085.1_3	1796	2353	protein	AAC_6p_Ia_fam-NCBIFAM	complete
	DACRZH010000085.1_4	2358	2783	protein	protein	complete
	DACRZH010000085.1_5	2835	3710	protein	ANT_3pp_AadA1-NCBIFAM	complete
	attc_001	3712	3771	attC	attC	complete
486	DACRZW010000059.1_3	1542	1889	protein	SMR_qac_E-NCBIFAM	complete
	attc_001	1992	2063	attC	attC	complete
	DACRZW010000059.1_4	2058	2687	protein	AAC_6p_Ib-NCBIFAM	complete
	P_intI1	2800	2834	Promoter	Pint_1	complete
	DACRZW010000059.1_5	2855	3607	protein	intI	complete
	DACRZW010000119.1_1	5	796	protein	intI	complete
	DACRZW010000119.1_2	964	1593	protein	AAC_6p_Ib-NCBIFAM	complete
	attc_001	1588	1659	attC	attC	complete
	DACRZW010000119.1_3	1762	2109	protein	SMR_qac_E-NCBIFAM	complete
487	DACRZL010000045.1_1	3	122	protein	protein	complete
	attc_001	238	297	attC	attC	complete
	DACRZL010000045.1_2	299	1174	protein	ANT_3pp_AadA1-NCBIFAM	complete
	DACRZL010000045.1_3	1226	1651	protein	protein	complete
	DACRZL010000045.1_4	1656	2213	protein	AAC_6p_Ia_fam-NCBIFAM	complete
	P_intI1	2338	2372	Promoter	Pint_1	complete
	DACRZL010000045.1_5	2393	3184	protein	intI	complete
	DACRZL010000087.1_2	825	1616	protein	intI	complete
	P_intI1	1636	1670	Promoter	Pint_1	complete
	attI1	1695	1753	attI	attI_1	complete
	DACRZL010000087.1_3	1796	2353	protein	AAC_6p_Ia_fam-NCBIFAM	complete
	DACRZL010000087.1_4	2358	2783	protein	protein	complete
	DACRZL010000087.1_5	2835	3710	protein	ANT_3pp_AadA1-NCBIFAM	complete
	attc_001	3712	3771	attC	attC	complete
488	DACRZN010000048.1_1	3	122	protein	protein	complete
	attc_001	238	297	attC	attC	complete
	DACRZN010000048.1_2	299	1174	protein	ANT_3pp_AadA1-NCBIFAM	complete
	DACRZN010000048.1_3	1226	1651	protein	protein	complete
	DACRZN010000048.1_4	1656	2213	protein	AAC_6p_Ia_fam-NCBIFAM	complete
	P_intI1	2338	2372	Promoter	Pint_1	complete
	DACRZN010000048.1_5	2393	3184	protein	intI	complete
	DACRZN010000095.1_2	825	1616	protein	intI	complete
	P_intI1	1636	1670	Promoter	Pint_1	complete
	attI1	1695	1753	attI	attI_1	complete

	DACRZN010000095.1_3	1796	2353	protein	AAC_6p_Ia_fam-NCBIFAM	complete
	DACRZN010000095.1_4	2358	2783	protein	protein	complete
	DACRZN010000095.1_5	2835	3710	protein	ANT_3pp_AadA1-NCBIFAM	complete
	attc_001	3712	3771	attC	attC	complete
491	DACRZP010000051.1_1	3	122	protein	protein	complete
	attc_001	238	297	attC	attC	complete
	DACRZP010000051.1_2	299	1174	protein	ANT_3pp_AadA1-NCBIFAM	complete
	DACRZP010000051.1_3	1226	1651	protein	protein	complete
	DACRZP010000051.1_4	1656	2213	protein	AAC_6p_Ia_fam-NCBIFAM	complete
	P_intI1	2338	2372	Promoter	Pint_1	complete
	DACRZP010000051.1_5	2393	3184	protein	intI	complete
	DACRZP010000087.1_2	825	1616	protein	intI	complete
	P_intI1	1636	1670	Promoter	Pint_1	complete
	attI1	1695	1753	attI	attI_1	complete
	DACRZP010000087.1_3	1796	2353	protein	AAC_6p_Ia_fam-NCBIFAM	complete
	DACRZP010000087.1_4	2358	2783	protein	protein	complete
	DACRZP010000087.1_5	2835	3710	protein	ANT_3pp_AadA1-NCBIFAM	complete
	attc_001	3712	3771	attC	attC	complete
494	DACRZQ010000051.1_1	3	122	protein	protein	complete
	attc_001	238	297	attC	attC	complete
	DACRZQ010000051.1_2	299	1174	protein	ANT_3pp_AadA1-NCBIFAM	complete
	DACRZQ010000051.1_3	1226	1651	protein	protein	complete
	DACRZQ010000051.1_4	1656	2213	protein	AAC_6p_Ia_fam-NCBIFAM	complete
	P_intI1	2338	2372	Promoter	Pint_1	complete
	DACRZQ010000051.1_5	2393	3184	protein	intI	complete
	DACRZQ010000086.1_2	825	1616	protein	intI	complete
	P_intI1	1636	1670	Promoter	Pint_1	complete
	attI1	1695	1753	attI	attI_1	complete
	DACRZQ010000086.1_3	1796	2353	protein	AAC_6p_Ia_fam-NCBIFAM	complete
	DACRZQ010000086.1_4	2358	2783	protein	protein	complete
	DACRZQ010000086.1_5	2835	3710	protein	ANT_3pp_AadA1-NCBIFAM	complete
	attc_001	3712	3771	attC	attC	complete
496	DACRZT010000062.1_3	1542	1889	protein	SMR_qac_E-NCBIFAM	complete
	attc_001	1992	2063	attC	attC	complete
	DACRZT010000062.1_4	2058	2687	protein	AAC_6p_Ib-NCBIFAM	complete
	P_intI1	2800	2834	Promoter	Pint_1	complete
	DACRZT010000062.1_5	2855	3607	protein	intI	complete
	DACRZT010000126.1_1	5	796	protein	intI	complete
	DACRZT010000126.1_2	964	1593	protein	AAC_6p_Ib-NCBIFAM	complete
	attc_001	1588	1659	attC	attC	complete
	DACRZT010000126.1_3	1762	2109	protein	SMR_qac_E-NCBIFAM	complete

498	DACRZU010000061.1_3	1542	1889	protein	SMR_qac_E-NCBIFAM	complete
	attc_001	1992	2063	attC	attC	complete
	DACRZU010000061.1_4	2058	2687	protein	AAC_6p_Ib-NCBIFAM	complete
	P_intI1	2800	2834	Promoter	Pint_1	complete
	DACRZU010000061.1_5	2855	3607	protein	intI	complete
	DACRZU010000120.1_1	5	796	protein	intI	complete
	DACRZU010000120.1_2	964	1593	protein	AAC_6p_Ib-NCBIFAM	complete
	attc_001	1588	1659	attC	attC	complete
	DACRZU010000120.1_3	1762	2109	protein	SMR_qac_E-NCBIFAM	complete
499	DACRZY010000058.1_3	1542	1889	protein	SMR_qac_E-NCBIFAM	complete
	attc_001	1992	2063	attC	attC	complete
	DACRZY010000058.1_4	2058	2687	protein	AAC_6p_Ib-NCBIFAM	complete
	P_intI1	2800	2834	Promoter	Pint_1	complete
	DACRZY010000058.1_5	2855	3607	protein	intI	complete
	DACRZY010000124.1_1	5	796	protein	intI	complete
	DACRZY010000124.1_2	964	1593	protein	AAC_6p_Ib-NCBIFAM	complete
	attc_001	1588	1659	attC	attC	complete
	DACRZY010000124.1_3	1762	2109	protein	SMR_qac_E-NCBIFAM	complete
501	DACRZZ010000048.1_1	3	122	protein	protein	complete
	attc_001	238	297	attC	attC	complete
	DACRZZ010000048.1_2	299	1174	protein	ANT_3pp_AadA1-NCBIFAM	complete
	DACRZZ010000048.1_3	1226	1651	protein	protein	complete
	DACRZZ010000048.1_4	1656	2213	protein	AAC_6p_Ia_fam-NCBIFAM	complete
	P_intI1	2338	2372	Promoter	Pint_1	complete
	DACRZZ010000048.1_5	2393	3184	protein	intI	complete
	DACRZZ010000086.1_2	825	1616	protein	intI	complete
	P_intI1	1636	1670	Promoter	Pint_1	complete
	attI1	1695	1753	attI	attI_1	complete
	DACRZZ010000086.1_3	1796	2353	protein	AAC_6p_Ia_fam-NCBIFAM	complete
	DACRZZ010000086.1_4	2358	2783	protein	protein	complete
	DACRZZ010000086.1_5	2835	3710	protein	ANT_3pp_AadA1-NCBIFAM	complete
	attc_001	3712	3771	attC	attC	complete
505	DACSAE010000055.1_4	1738	2085	protein	SMR_qac_E-NCBIFAM	complete
	attc_001	2188	2259	attC	attC	complete
	DACSAE010000055.1_5	2254	2883	protein	AAC_6p_Ib-NCBIFAM	complete
	attI1	2913	2971	attI	attI_1	complete
	P_intI1	2996	3030	Promoter	Pint_1	complete
	DACSAE010000055.1_6	3051	3842	protein	intI	complete
	DACSAE010000117.1_2	825	1616	protein	intI	complete
	P_intI1	1636	1670	Promoter	Pint_1	complete
	attI1	1695	1753	attI	attI_1	complete

	DACSAE010000117.1_3	1784	2413	protein	AAC_6p_Ib-NCBIFAM	complete
	attc_001	2408	2479	attC	attC	complete
	DACSAE010000117.1_4	2582	2929	protein	SMR_qac_E-NCBIFAM	complete
507	DACSAJ010000058.1_3	1542	1889	protein	SMR_qac_E-NCBIFAM	complete
	attc_001	1992	2063	attC	attC	complete
	DACSAJ010000058.1_4	2058	2687	protein	AAC_6p_Ib-NCBIFAM	complete
	attI1	2717	2775	attI	attI_1	complete
	P_intI1	2800	2834	Promoter	Pint_1	complete
	DACSAJ010000058.1_5	2855	3646	protein	intI	complete
	DACSAJ010000121.1_2	825	1616	protein	intI	complete
	P_intI1	1636	1670	Promoter	Pint_1	complete
	attI1	1695	1753	attI	attI_1	complete
	DACSAJ010000121.1_3	1784	2413	protein	AAC_6p_Ib-NCBIFAM	complete
	attc_001	2408	2479	attC	attC	complete
	DACSAJ010000121.1_4	2582	2929	protein	SMR_qac_E-NCBIFAM	complete
514	DACSAK010000074.1_1	3	122	protein	protein	complete
	attc_001	238	297	attC	attC	complete
	DACSAK010000074.1_2	299	1174	protein	ANT_3pp_AadA1-NCBIFAM	complete
	DACSAK010000074.1_3	1226	1651	protein	protein	complete
	DACSAK010000074.1_4	1656	2213	protein	AAC_6p_Ia_fam-NCBIFAM	complete
	P_intI1	2338	2372	Promoter	Pint_1	complete
	DACSAK010000074.1_5	2393	3184	protein	intI	complete
	DACSAK010000116.1_2	825	1616	protein	intI	complete
	P_intI1	1636	1670	Promoter	Pint_1	complete
	attI1	1695	1753	attI	attI_1	complete
	DACSAK010000116.1_3	1796	2353	protein	AAC_6p_Ia_fam-NCBIFAM	complete
	DACSAK010000116.1_4	2358	2783	protein	protein	complete
	attc_001	2798	2844	attC	attC	complete
515	DACSAN010000049.1_1	3	122	protein	protein	complete
	attc_001	238	297	attC	attC	complete
	DACSAN010000049.1_2	299	1174	protein	ANT_3pp_AadA1-NCBIFAM	complete
	DACSAN010000049.1_3	1226	1651	protein	protein	complete
	DACSAN010000049.1_4	1656	2213	protein	AAC_6p_Ia_fam-NCBIFAM	complete
	P_intI1	2338	2372	Promoter	Pint_1	complete
	DACSAN010000049.1_5	2393	3184	protein	intI	complete
	DACSAN010000093.1_2	825	1616	protein	intI	complete
	P_intI1	1636	1670	Promoter	Pint_1	complete
	attI1	1695	1753	attI	attI_1	complete
	DACSAN010000093.1_3	1796	2353	protein	AAC_6p_Ia_fam-NCBIFAM	complete
	DACSAN010000093.1_4	2358	2783	protein	protein	complete
	DACSAN010000093.1_5	2835	3710	protein	ANT_3pp_AadA1-NCBIFAM	complete

	attc_001	3712	3771	attC	attC	complete
517	DACSBM010000061.1_3	1542	1889	protein	SMR_qac_E-NCBIFAM	complete
	attc_001	1992	2063	attC	attC	complete
	DACSBM010000061.1_4	2058	2687	protein	AAC_6p_Ib-NCBIFAM	complete
	attI1	2717	2775	attI	attI_1	complete
	P_intI1	2800	2834	Promoter	Pint_1	complete
	DACSBM010000061.1_5	2855	3646	protein	intI	complete
	DACSBM010000131.1_2	825	1616	protein	intI	complete
	P_intI1	1636	1670	Promoter	Pint_1	complete
	attI1	1695	1753	attI	attI_1	complete
	DACSBM010000131.1_3	1784	2413	protein	AAC_6p_Ib-NCBIFAM	complete
	attc_001	2408	2479	attC	attC	complete
	DACSBM010000131.1_4	2582	2929	protein	SMR_qac_E-NCBIFAM	complete
518	DACSBH010000060.1_3	1542	1889	protein	SMR_qac_E-NCBIFAM	complete
	attc_001	1992	2063	attC	attC	complete
	DACSBH010000060.1_4	2058	2687	protein	AAC_6p_Ib-NCBIFAM	complete
	attI1	2717	2775	attI	attI_1	complete
	P_intI1	2800	2834	Promoter	Pint_1	complete
	DACSBH010000060.1_5	2855	3646	protein	intI	complete
	DACSBH010000141.1_2	825	1616	protein	intI	complete
	P_intI1	1636	1670	Promoter	Pint_1	complete
	attI1	1695	1753	attI	attI_1	complete
	DACSBH010000141.1_3	1784	2413	protein	AAC_6p_Ib-NCBIFAM	complete
	attc_001	2408	2479	attC	attC	complete
	DACSBH010000141.1_4	2582	2929	protein	SMR_qac_E-NCBIFAM	complete
521	DACSAP010000057.1_3	1542	1889	protein	SMR_qac_E-NCBIFAM	complete
	attc_001	1992	2063	attC	attC	complete
	DACSAP010000057.1_4	2058	2687	protein	AAC_6p_Ib-NCBIFAM	complete
	P_intI1	2800	2834	Promoter	Pint_1	complete
	DACSAP010000057.1_5	2855	3607	protein	intI	complete
	DACSAP010000125.1_1	5	796	protein	intI	complete
	DACSAP010000125.1_2	964	1593	protein	AAC_6p_Ib-NCBIFAM	complete
	attc_001	1588	1659	attC	attC	complete
	DACSAP010000125.1_3	1762	2109	protein	SMR_qac_E-NCBIFAM	complete
522	DACSBK010000053.1_1	3	122	protein	protein	complete
	attc_001	238	297	attC	attC	complete
	DACSBK010000053.1_2	299	1174	protein	ANT_3pp_AadA1-NCBIFAM	complete
	DACSBK010000053.1_3	1226	1651	protein	protein	complete
	DACSBK010000053.1_4	1656	2213	protein	AAC_6p_Ia_fam-NCBIFAM	complete
	P_intI1	2338	2372	Promoter	Pint_1	complete
	DACSBK010000053.1_5	2393	3184	protein	intI	complete

	DACSBK010000089.1_2	825	1616	protein	intI	complete
	P_intI1	1636	1670	Promoter	Pint_1	complete
	attI1	1695	1753	attI	attI_1	complete
	DACSBK010000089.1_3	1796	2353	protein	AAC_6p_Ia_fam-NCBIFAM	complete
	DACSBK010000089.1_4	2358	2783	protein	protein	complete
	DACSBK010000089.1_5	2835	3710	protein	ANT_3pp_AadA1-NCBIFAM	complete
	attc_001	3712	3771	attC	attC	complete
523	attc_001	46	105	attC	attC	complete
	DACSBP010000049.1_1	107	982	protein	ANT_3pp_AadA1-NCBIFAM	complete
	DACSBP010000049.1_2	1034	1459	protein	protein	complete
	DACSBP010000049.1_3	1464	2021	protein	AAC_6p_Ia_fam-NCBIFAM	complete
	P_intI1	2146	2180	Promoter	Pint_1	complete
	DACSBP010000049.1_4	2201	2992	protein	intI	complete
	DACSBP010000088.1_2	825	1616	protein	intI	complete
	P_intI1	1636	1670	Promoter	Pint_1	complete
	attI1	1695	1753	attI	attI_1	complete
	DACSBP010000088.1_3	1796	2353	protein	AAC_6p_Ia_fam-NCBIFAM	complete
	DACSBP010000088.1_4	2358	2783	protein	protein	complete
	DACSBP010000088.1_5	2835	3710	protein	ANT_3pp_AadA1-NCBIFAM	complete
	attc_001	3712	3771	attC	attC	complete
524	DACSBL010000053.1_3	1542	1889	protein	SMR_qac_E-NCBIFAM	complete
	attc_001	1992	2063	attC	attC	complete
	DACSBL010000053.1_4	2058	2687	protein	AAC_6p_Ib-NCBIFAM	complete
	P_intI1	2800	2834	Promoter	Pint_1	complete
	DACSBL010000053.1_5	2855	3607	protein	intI	complete
	DACSBL010000115.1_1	5	796	protein	intI	complete
	DACSBL010000115.1_2	964	1593	protein	AAC_6p_Ib-NCBIFAM	complete
	attc_001	1588	1659	attC	attC	complete
	DACSBL010000115.1_3	1762	2109	protein	SMR_qac_E-NCBIFAM	complete
527	DACSBI010000054.1_1	3	122	protein	protein	complete
	attc_001	238	297	attC	attC	complete
	DACSBI010000054.1_2	299	1174	protein	ANT_3pp_AadA1-NCBIFAM	complete
	DACSBI010000054.1_3	1226	1651	protein	protein	complete
	DACSBI010000054.1_4	1656	2213	protein	AAC_6p_Ia_fam-NCBIFAM	complete
	P_intI1	2338	2372	Promoter	Pint_1	complete
	DACSBI010000054.1_5	2393	3184	protein	intI	complete
	DACSBI010000095.1_2	825	1616	protein	intI	complete
	P_intI1	1636	1670	Promoter	Pint_1	complete
	attI1	1695	1753	attI	attI_1	complete
	DACSBI010000095.1_3	1796	2353	protein	AAC_6p_Ia_fam-NCBIFAM	complete
	DACSBI010000095.1_4	2358	2783	protein	protein	complete

	DACSBI010000095.1_5	2835	3710	protein	ANT_3pp_AadA1-NCBIFAM	complete
	attc_001	3712	3771	attC	attC	complete
528	DACSAX010000054.1_1	3	122	protein	protein	complete
	attc_001	238	297	attC	attC	complete
	DACSAX010000054.1_2	299	1174	protein	ANT_3pp_AadA1-NCBIFAM	complete
	DACSAX010000054.1_3	1226	1651	protein	protein	complete
	DACSAX010000054.1_4	1656	2213	protein	AAC_6p_Ia_fam-NCBIFAM	complete
	P_intI1	2338	2372	Promoter	Pint_1	complete
	DACSAX010000054.1_5	2393	3184	protein	intI	complete
	DACSAX010000095.1_2	825	1616	protein	intI	complete
	P_intI1	1636	1670	Promoter	Pint_1	complete
	attI1	1695	1753	attI	attI_1	complete
	DACSAX010000095.1_3	1796	2353	protein	AAC_6p_Ia_fam-NCBIFAM	complete
	DACSAX010000095.1_4	2358	2783	protein	protein	complete
	DACSAX010000095.1_5	2835	3710	protein	ANT_3pp_AadA1-NCBIFAM	complete
	attc_001	3712	3771	attC	attC	complete
835	NZ_VCOZ01000051.1_2	912	1703	protein	intI	complete
	P_intI1	1723	1757	Promoter	Pint_1	complete
	attI1	1782	1840	attI	attI_1	complete
	NZ_VCOZ01000051.1_3	1883	2440	protein	AAC_6p_Ia_fam-NCBIFAM	complete
	NZ_VCOZ01000051.1_4	2445	2870	protein	protein	complete
	NZ_VCOZ01000051.1_5	2922	3797	protein	ANT_3pp_AadA1-NCBIFAM	complete
	attc_001	3799	3858	attC	attC	complete
	NZ_VCOZ01000051.1_6	3974	4090	protein	protein	complete
836	NZ_VCOY01000046.1_1	3	230	protein	protein	complete
	attc_001	346	405	attC	attC	complete
	NZ_VCOY01000046.1_2	407	1282	protein	ANT_3pp_AadA1-NCBIFAM	complete
	NZ_VCOY01000046.1_3	1334	1759	protein	protein	complete
	NZ_VCOY01000046.1_4	1764	2321	protein	AAC_6p_Ia_fam-NCBIFAM	complete
	P_intI1	2446	2480	Promoter	Pint_1	complete
	NZ_VCOY01000046.1_5	2501	3292	protein	intI	complete
850	DACTXH010000051.1_1	3	122	protein	protein	complete
	attc_001	238	297	attC	attC	complete
	DACTXH010000051.1_2	299	1174	protein	ANT_3pp_AadA1-NCBIFAM	complete
	DACTXH010000051.1_3	1226	1651	protein	protein	complete
	DACTXH010000051.1_4	1656	2213	protein	AAC_6p_Ia_fam-NCBIFAM	complete
	P_intI1	2338	2372	Promoter	Pint_1	complete
	DACTXH010000051.1_5	2393	3184	protein	intI	complete
	DACTXH010000084.1_2	785	1576	protein	intI	complete
	P_intI1	1596	1630	Promoter	Pint_1	complete
	attI1	1655	1713	attI	attI_1	complete

	DACTXH010000084.1_3	1756	2313	protein	AAC_6p_Ia_fam-NCBIFAM	complete
	DACTXH010000084.1_4	2318	2743	protein	protein	complete
	DACTXH010000084.1_5	2795	3670	protein	ANT_3pp_AadA1-NCBIFAM	complete
	attc_001	3672	3731	attC	attC	complete
854	DACTXK010000048.1_1	3	122	protein	protein	complete
	attc_001	238	297	attC	attC	complete
	DACTXK010000048.1_2	299	1174	protein	ANT_3pp_AadA1-NCBIFAM	complete
	DACTXK010000048.1_3	1226	1651	protein	protein	complete
	DACTXK010000048.1_4	1656	2213	protein	AAC_6p_Ia_fam-NCBIFAM	complete
	P_intI1	2338	2372	Promoter	Pint_1	complete
	DACTXK010000048.1_5	2393	3184	protein	intI	complete
	DACTXK010000088.1_2	785	1576	protein	intI	complete
	P_intI1	1596	1630	Promoter	Pint_1	complete
	attI1	1655	1713	attI	attI_1	complete
	DACTXK010000088.1_3	1756	2313	protein	AAC_6p_Ia_fam-NCBIFAM	complete
	DACTXK010000088.1_4	2318	2743	protein	protein	complete
	DACTXK010000088.1_5	2795	3670	protein	ANT_3pp_AadA1-NCBIFAM	complete
	attc_001	3672	3731	attC	attC	complete
856	attc_001	45	104	attC	attC	complete
	DACTXP010000050.1_1	106	981	protein	ANT_3pp_AadA1-NCBIFAM	complete
	DACTXP010000050.1_2	1033	1458	protein	protein	complete
	DACTXP010000050.1_3	1463	2020	protein	AAC_6p_Ia_fam-NCBIFAM	complete
	P_intI1	2145	2179	Promoter	Pint_1	complete
	DACTXP010000050.1_4	2200	2991	protein	intI	complete
	DACTXP010000086.1_2	785	1576	protein	intI	complete
	P_intI1	1596	1630	Promoter	Pint_1	complete
	attI1	1655	1713	attI	attI_1	complete
	DACTXP010000086.1_3	1756	2313	protein	AAC_6p_Ia_fam-NCBIFAM	complete
	DACTXP010000086.1_4	2318	2743	protein	protein	complete
	DACTXP010000086.1_5	2795	3670	protein	ANT_3pp_AadA1-NCBIFAM	complete
	attc_001	3672	3731	attC	attC	complete
857	DACTXY010000056.1_3	1542	1889	protein	SMR_qac_E-NCBIFAM	complete
	attc_001	1992	2063	attC	attC	complete
	DACTXY010000056.1_4	2058	2687	protein	AAC_6p_Ib-NCBIFAM	complete
	P_intI1	2800	2834	Promoter	Pint_1	complete
	DACTXY010000056.1_5	2855	3607	protein	intI	complete
	DACTXY010000121.1_1	1	756	protein	intI	complete
	DACTXY010000121.1_2	924	1553	protein	AAC_6p_Ib-NCBIFAM	complete
	attc_001	1548	1619	attC	attC	complete
	DACTXY010000121.1_3	1722	2069	protein	SMR_qac_E-NCBIFAM	complete
859	DACTXV010000047.1_1	3	122	protein	protein	complete

	attc_001	238	297	attC	attC	complete
	DACTXV010000047.1_2	299	1174	protein	ANT_3pp_AadA1-NCBIFAM	complete
	DACTXV010000047.1_3	1226	1651	protein	protein	complete
	DACTXV010000047.1_4	1656	2213	protein	AAC_6p_Ia_fam-NCBIFAM	complete
	P_intI1	2338	2372	Promoter	Pint_1	complete
	DACTXV010000047.1_5	2393	3184	protein	intI	complete
	DACTXV010000082.1_2	785	1576	protein	intI	complete
	P_intI1	1596	1630	Promoter	Pint_1	complete
	attI1	1655	1713	attI	attI_1	complete
	DACTXV010000082.1_3	1756	2313	protein	AAC_6p_Ia_fam-NCBIFAM	complete
	DACTXV010000082.1_4	2318	2743	protein	protein	complete
	DACTXV010000082.1_5	2795	3670	protein	ANT_3pp_AadA1-NCBIFAM	complete
	attc_001	3672	3731	attC	attC	complete
860	DACTXW010000055.1_3	1542	1889	protein	SMR_qac_E-NCBIFAM	complete
	attc_001	1992	2063	attC	attC	complete
	DACTXW010000055.1_4	2058	2687	protein	AAC_6p_Ib-NCBIFAM	complete
	P_intI1	2800	2834	Promoter	Pint_1	complete
	DACTXW010000055.1_5	2855	3607	protein	intI	complete
	DACTXW010000119.1_1	1	756	protein	intI	complete
	DACTXW010000119.1_2	924	1553	protein	AAC_6p_Ib-NCBIFAM	complete
	attc_001	1548	1619	attC	attC	complete
	DACTXW010000119.1_3	1722	2069	protein	SMR_qac_E-NCBIFAM	complete
861	DACTXT010000060.1_1	3	122	protein	protein	complete
	attc_001	238	297	attC	attC	complete
	DACTXT010000060.1_2	299	1174	protein	ANT_3pp_AadA1-NCBIFAM	complete
	DACTXT010000060.1_3	1226	1651	protein	protein	complete
	DACTXT010000060.1_4	1656	2213	protein	AAC_6p_Ia_fam-NCBIFAM	complete
	P_intI1	2338	2372	Promoter	Pint_1	complete
	DACTXT010000060.1_5	2393	3184	protein	intI	complete
	DACTXT010000099.1_2	785	1576	protein	intI	complete
	P_intI1	1596	1630	Promoter	Pint_1	complete
	attI1	1655	1713	attI	attI_1	complete
	DACTXT010000099.1_3	1756	2313	protein	AAC_6p_Ia_fam-NCBIFAM	complete
	DACTXT010000099.1_4	2318	2743	protein	protein	complete
	DACTXT010000099.1_5	2795	3670	protein	ANT_3pp_AadA1-NCBIFAM	complete
	attc_001	3672	3731	attC	attC	complete
863	DACTYA010000052.1_1	3	122	protein	protein	complete
	attc_001	238	297	attC	attC	complete
	DACTYA010000052.1_2	299	1174	protein	ANT_3pp_AadA1-NCBIFAM	complete
	DACTYA010000052.1_3	1226	1651	protein	protein	complete
	DACTYA010000052.1_4	1656	2213	protein	AAC_6p_Ia_fam-NCBIFAM	complete

	P_intI1	2338	2372	Promoter	Pint_1	complete
	DACTYA010000052.1_5	2393	3184	protein	intI	complete
	DACTYA010000087.1_2	785	1576	protein	intI	complete
	P_intI1	1596	1630	Promoter	Pint_1	complete
	attI1	1655	1713	attI	attI_1	complete
	DACTYA010000087.1_3	1756	2313	protein	AAC_6p_Ia_fam-NCBIFAM	complete
	DACTYA010000087.1_4	2318	2743	protein	protein	complete
	DACTYA010000087.1_5	2795	3670	protein	ANT_3pp_AadA1-NCBIFAM	complete
	attc_001	3672	3731	attC	attC	complete
864	DACTXR010000048.1_1	3	122	protein	protein	complete
	attc_001	238	297	attC	attC	complete
	DACTXR010000048.1_2	299	1174	protein	ANT_3pp_AadA1-NCBIFAM	complete
	DACTXR010000048.1_3	1226	1651	protein	protein	complete
	DACTXR010000048.1_4	1656	2213	protein	AAC_6p_Ia_fam-NCBIFAM	complete
	DACTXR010000048.1_5	2393	3184	protein	intI	complete
	DACTXR010000086.1_2	785	1576	protein	intI	complete
	attI1	1655	1713	attI	attI_1	complete
	DACTXR010000086.1_3	1756	2313	protein	AAC_6p_Ia_fam-NCBIFAM	complete
	DACTXR010000086.1_4	2318	2743	protein	protein	complete
	DACTXR010000086.1_5	2795	3670	protein	ANT_3pp_AadA1-NCBIFAM	complete
	attc_001	3672	3731	attC	attC	complete
865	DACTXS010000056.1_3	1542	1889	protein	SMR_qac_E-NCBIFAM	complete
	attc_001	1992	2063	attC	attC	complete
	DACTXS010000056.1_4	2058	2687	protein	AAC_6p_Ib-NCBIFAM	complete
	P_intI1	2800	2834	Promoter	Pint_1	complete
	DACTXS010000056.1_5	2855	3607	protein	intI	complete
	DACTXS010000122.1_1	1	756	protein	intI	complete
	DACTXS010000122.1_2	924	1553	protein	AAC_6p_Ib-NCBIFAM	complete
	attc_001	1548	1619	attC	attC	complete
	DACTXS010000122.1_3	1722	2069	protein	SMR_qac_E-NCBIFAM	complete
867	DACTYE010000058.1_3	1542	1889	protein	SMR_qac_E-NCBIFAM	complete
	attc_001	1992	2063	attC	attC	complete
	DACTYE010000058.1_4	2058	2687	protein	AAC_6p_Ib-NCBIFAM	complete
	P_intI1	2800	2834	Promoter	Pint_1	complete
	DACTYE010000058.1_5	2855	3607	protein	intI	complete
	DACTYE010000122.1_1	1	756	protein	intI	complete
	DACTYE010000122.1_2	924	1553	protein	AAC_6p_Ib-NCBIFAM	complete
	attc_001	1548	1619	attC	attC	complete
	DACTYE010000122.1_3	1722	2069	protein	SMR_qac_E-NCBIFAM	complete
868	DACTXX010000056.1_3	1542	1889	protein	SMR_qac_E-NCBIFAM	complete
	attc_001	1992	2063	attC	attC	complete

	DACTXX010000056.1_4	2058	2687	protein	AAC_6p_Ib-NCBIFAM	complete
	P_intI1	2800	2834	Promoter	Pint_1	complete
	DACTXX010000056.1_5	2855	3607	protein	intI	complete
	DACTXX010000118.1_1	1	756	protein	intI	complete
	DACTXX010000118.1_2	924	1553	protein	AAC_6p_Ib-NCBIFAM	complete
	attc_001	1548	1619	attC	attC	complete
	DACTXX010000118.1_3	1722	2069	protein	SMR_qac_E-NCBIFAM	complete
869	DACTYK010000054.1_1	3	122	protein	protein	complete
	attc_001	238	297	attC	attC	complete
	DACTYK010000054.1_2	299	1174	protein	ANT_3pp_AadA1-NCBIFAM	complete
	DACTYK010000054.1_3	1226	1651	protein	protein	complete
	DACTYK010000054.1_4	1656	2213	protein	AAC_6p_Ia_fam-NCBIFAM	complete
	P_intI1	2338	2372	Promoter	Pint_1	complete
	DACTYK010000054.1_5	2393	3184	protein	intI	complete
	DACTYK010000094.1_2	785	1576	protein	intI	complete
	P_intI1	1596	1630	Promoter	Pint_1	complete
	attI1	1655	1713	attI	attI_1	complete
	DACTYK010000094.1_3	1756	2313	protein	AAC_6p_Ia_fam-NCBIFAM	complete
	DACTYK010000094.1_4	2318	2743	protein	protein	complete
	DACTYK010000094.1_5	2795	3670	protein	ANT_3pp_AadA1-NCBIFAM	complete
	attc_001	3672	3731	attC	attC	complete
871	DACTYJ010000048.1_1	3	224	protein	protein	complete
	attc_001	340	399	attC	attC	complete
	DACTYJ010000048.1_2	401	1276	protein	ANT_3pp_AadA1-NCBIFAM	complete
	DACTYJ010000048.1_3	1328	1753	protein	protein	complete
	DACTYJ010000048.1_4	1758	2315	protein	AAC_6p_Ia_fam-NCBIFAM	complete
	P_intI1	2440	2474	Promoter	Pint_1	complete
	DACTYJ010000048.1_5	2495	3286	protein	intI	complete
	DACTYJ010000091.1_2	785	1576	protein	intI	complete
	P_intI1	1596	1630	Promoter	Pint_1	complete
	attI1	1655	1713	attI	attI_1	complete
	DACTYJ010000091.1_3	1756	2313	protein	AAC_6p_Ia_fam-NCBIFAM	complete
	DACTYJ010000091.1_4	2318	2743	protein	protein	complete
	DACTYJ010000091.1_5	2795	3670	protein	ANT_3pp_AadA1-NCBIFAM	complete
	attc_001	3672	3731	attC	attC	complete
872	DACTYI010000049.1_1	3	122	protein	protein	complete
	attc_001	238	297	attC	attC	complete
	DACTYI010000049.1_2	299	1174	protein	ANT_3pp_AadA1-NCBIFAM	complete
	DACTYI010000049.1_3	1226	1651	protein	protein	complete
	DACTYI010000049.1_4	1656	2213	protein	AAC_6p_Ia_fam-NCBIFAM	complete
	P_intI1	2338	2372	Promoter	Pint_1	complete

	DACTYI010000049.1_5	2393	3184	protein	intI	complete
	DACTYI010000087.1_2	785	1576	protein	intI	complete
	P_intI1	1596	1630	Promoter	Pint_1	complete
	attI1	1655	1713	attI	attI_1	complete
	DACTYI010000087.1_3	1756	2313	protein	AAC_6p_Ia_fam-NCBIFAM	complete
	DACTYI010000087.1_4	2318	2743	protein	protein	complete
	DACTYI010000087.1_5	2795	3670	protein	ANT_3pp_AadA1-NCBIFAM	complete
	attc_001	3672	3731	attC	attC	complete
873	DACTYC010000048.1_1	3	122	protein	protein	complete
	attc_001	238	297	attC	attC	complete
	DACTYC010000048.1_2	299	1174	protein	ANT_3pp_AadA1-NCBIFAM	complete
	DACTYC010000048.1_3	1226	1651	protein	protein	complete
	DACTYC010000048.1_4	1656	2213	protein	AAC_6p_Ia_fam-NCBIFAM	complete
	P_intI1	2338	2372	Promoter	Pint_1	complete
	DACTYC010000048.1_5	2393	3184	protein	intI	complete
	DACTYC010000088.1_2	785	1576	protein	intI	complete
	P_intI1	1596	1630	Promoter	Pint_1	complete
	attI1	1655	1713	attI	attI_1	complete
	DACTYC010000088.1_3	1756	2313	protein	AAC_6p_Ia_fam-NCBIFAM	complete
	DACTYC010000088.1_4	2318	2743	protein	protein	complete
	DACTYC010000088.1_5	2795	3670	protein	ANT_3pp_AadA1-NCBIFAM	complete
	attc_001	3672	3731	attC	attC	complete
876	DACTYH010000050.1_1	3	122	protein	protein	complete
	attc_001	238	297	attC	attC	complete
	DACTYH010000050.1_2	299	1174	protein	ANT_3pp_AadA1-NCBIFAM	complete
	DACTYH010000050.1_3	1226	1651	protein	protein	complete
	DACTYH010000050.1_4	1656	2213	protein	AAC_6p_Ia_fam-NCBIFAM	complete
	P_intI1	2338	2372	Promoter	Pint_1	complete
	DACTYH010000050.1_5	2393	3184	protein	intI	complete
	DACTYH010000090.1_2	785	1576	protein	intI	complete
	P_intI1	1596	1630	Promoter	Pint_1	complete
	attI1	1655	1713	attI	attI_1	complete
	DACTYH010000090.1_3	1756	2313	protein	AAC_6p_Ia_fam-NCBIFAM	complete
	DACTYH010000090.1_4	2318	2743	protein	protein	complete
	DACTYH010000090.1_5	2795	3670	protein	ANT_3pp_AadA1-NCBIFAM	complete
	attc_001	3672	3731	attC	attC	complete
877	DACTXM010000051.1_1	3	122	protein	protein	complete
	attc_001	238	297	attC	attC	complete
	DACTXM010000051.1_2	299	1174	protein	ANT_3pp_AadA1-NCBIFAM	complete
	DACTXM010000051.1_3	1226	1651	protein	protein	complete
	DACTXM010000051.1_4	1656	2213	protein	AAC_6p_Ia_fam-NCBIFAM	complete

	P_intI1	2338	2372	Promoter	Pint_1	complete
	DACTXM010000051.1_5	2393	3184	protein	intI	complete
	DACTXM010000088.1_2	785	1576	protein	intI	complete
	P_intI1	1596	1630	Promoter	Pint_1	complete
	attI1	1655	1713	attI	attI_1	complete
	DACTXM010000088.1_3	1756	2313	protein	AAC_6p_Ia_fam-NCBIFAM	complete
	DACTXM010000088.1_4	2318	2743	protein	protein	complete
	DACTXM010000088.1_5	2795	3670	protein	ANT_3pp_AadA1-NCBIFAM	complete
	attc_001	3672	3731	attC	attC	complete
878	DACTYG010000049.1_1	3	122	protein	protein	complete
	attc_001	238	297	attC	attC	complete
	DACTYG010000049.1_2	299	1174	protein	ANT_3pp_AadA1-NCBIFAM	complete
	DACTYG010000049.1_3	1226	1651	protein	protein	complete
	DACTYG010000049.1_4	1656	2213	protein	AAC_6p_Ia_fam-NCBIFAM	complete
	P_intI1	2338	2372	Promoter	Pint_1	complete
	DACTYG010000049.1_5	2393	3184	protein	intI	complete
	DACTYG010000084.1_2	785	1576	protein	intI	complete
	P_intI1	1596	1630	Promoter	Pint_1	complete
	attI1	1655	1713	attI	attI_1	complete
	DACTYG010000084.1_3	1756	2313	protein	AAC_6p_Ia_fam-NCBIFAM	complete
	DACTYG010000084.1_4	2318	2743	protein	protein	complete
	DACTYG010000084.1_5	2795	3670	protein	ANT_3pp_AadA1-NCBIFAM	complete
	attc_001	3672	3731	attC	attC	complete
880	DACTYM010000052.1_1	3	122	protein	protein	complete
	attc_001	238	297	attC	attC	complete
	DACTYM010000052.1_2	299	1174	protein	ANT_3pp_AadA1-NCBIFAM	complete
	DACTYM010000052.1_3	1226	1651	protein	protein	complete
	DACTYM010000052.1_4	1656	2213	protein	AAC_6p_Ia_fam-NCBIFAM	complete
	P_intI1	2338	2372	Promoter	Pint_1	complete
	DACTYM010000052.1_5	2393	3184	protein	intI	complete
	DACTYM010000086.1_2	785	1576	protein	intI	complete
	P_intI1	1596	1630	Promoter	Pint_1	complete
	attI1	1655	1713	attI	attI_1	complete
	DACTYM010000086.1_3	1756	2313	protein	AAC_6p_Ia_fam-NCBIFAM	complete
	DACTYM010000086.1_4	2318	2743	protein	protein	complete
	DACTYM010000086.1_5	2795	3670	protein	ANT_3pp_AadA1-NCBIFAM	complete
	attc_001	3672	3731	attC	attC	complete
881	DACTYL010000052.1_1	3	122	protein	protein	complete
	attc_001	238	297	attC	attC	complete
	DACTYL010000052.1_2	299	1174	protein	ANT_3pp_AadA1-NCBIFAM	complete
	DACTYL010000052.1_3	1226	1651	protein	protein	complete

	DACTYL010000052.1_4	1656	2213	protein	AAC_6p_Ia_fam-NCBIFAM	complete
	P_intI1	2338	2372	Promoter	Pint_1	complete
	DACTYL010000052.1_5	2393	3184	protein	intI	complete
	DACTYL010000090.1_2	785	1576	protein	intI	complete
	P_intI1	1596	1630	Promoter	Pint_1	complete
	attI1	1655	1713	attI	attI_1	complete
	DACTYL010000090.1_3	1756	2313	protein	AAC_6p_Ia_fam-NCBIFAM	complete
	DACTYL010000090.1_4	2318	2743	protein	protein	complete
	DACTYL010000090.1_5	2795	3670	protein	ANT_3pp_AadA1-NCBIFAM	complete
	attc_001	3672	3731	attC	attC	complete
882	DACTYP010000051.1_1	3	122	protein	protein	complete
	attc_001	238	297	attC	attC	complete
	DACTYP010000051.1_2	299	1174	protein	ANT_3pp_AadA1-NCBIFAM	complete
	DACTYP010000051.1_3	1226	1651	protein	protein	complete
	DACTYP010000051.1_4	1656	2213	protein	AAC_6p_Ia_fam-NCBIFAM	complete
	P_intI1	2338	2372	Promoter	Pint_1	complete
	DACTYP010000051.1_5	2393	3184	protein	intI	complete
	DACTYP010000094.1_2	785	1576	protein	intI	complete
	P_intI1	1596	1630	Promoter	Pint_1	complete
	attI1	1655	1713	attI	attI_1	complete
	DACTYP010000094.1_3	1756	2313	protein	AAC_6p_Ia_fam-NCBIFAM	complete
	DACTYP010000094.1_4	2318	2743	protein	protein	complete
	DACTYP010000094.1_5	2795	3670	protein	ANT_3pp_AadA1-NCBIFAM	complete
	attc_001	3672	3731	attC	attC	complete
884	attc_001	46	105	attC	attC	complete
	DACTYO010000052.1_1	107	982	protein	ANT_3pp_AadA1-NCBIFAM	complete
	DACTYO010000052.1_2	1034	1459	protein	protein	complete
	DACTYO010000052.1_3	1464	2021	protein	AAC_6p_Ia_fam-NCBIFAM	complete
	P_intI1	2146	2180	Promoter	Pint_1	complete
	DACTYO010000052.1_4	2201	2992	protein	intI	complete
	DACTYO010000084.1_2	785	1576	protein	intI	complete
	P_intI1	1596	1630	Promoter	Pint_1	complete
	attI1	1655	1713	attI	attI_1	complete
	DACTYO010000084.1_3	1756	2313	protein	AAC_6p_Ia_fam-NCBIFAM	complete
	DACTYO010000084.1_4	2318	2743	protein	protein	complete
	DACTYO010000084.1_5	2795	3670	protein	ANT_3pp_AadA1-NCBIFAM	complete
	attc_001	3672	3731	attC	attC	complete
885	DACTYQ010000059.1_1	3	122	protein	protein	complete
	attc_001	238	297	attC	attC	complete
	DACTYQ010000059.1_2	299	1174	protein	ANT_3pp_AadA1-NCBIFAM	complete
	DACTYQ010000059.1_3	1226	1651	protein	protein	complete

	DACTYQ010000059.1_4	1656	2213	protein	AAC_6p_Ia_fam-NCBIFAM	complete
	P_intI1	2338	2372	Promoter	Pint_1	complete
	DACTYQ010000059.1_5	2393	3184	protein	intI	complete
	DACTYQ010000098.1_2	785	1576	protein	intI	complete
	P_intI1	1596	1630	Promoter	Pint_1	complete
	attI1	1655	1713	attI	attI_1	complete
	DACTYQ010000098.1_3	1756	2313	protein	AAC_6p_Ia_fam-NCBIFAM	complete
	DACTYQ010000098.1_4	2318	2743	protein	protein	complete
	DACTYQ010000098.1_5	2795	3670	protein	ANT_3pp_AadA1-NCBIFAM	complete
	attc_001	3672	3731	attC	attC	complete
886	DACTYS010000057.1_3	1542	1889	protein	SMR_qac_E-NCBIFAM	complete
	attc_001	1992	2063	attC	attC	complete
	DACTYS010000057.1_4	2058	2687	protein	AAC_6p_Ib-NCBIFAM	complete
	P_intI1	2800	2834	Promoter	Pint_1	complete
	DACTYS010000057.1_5	2855	3607	protein	intI	complete
	DACTYS010000117.1_1	1	756	protein	intI	complete
	DACTYS010000117.1_2	924	1553	protein	AAC_6p_Ib-NCBIFAM	complete
	attc_001	1548	1619	attC	attC	complete
	DACTYS010000117.1_3	1722	2069	protein	SMR_qac_E-NCBIFAM	complete
887	DACTYR010000057.1_3	1542	1889	protein	SMR_qac_E-NCBIFAM	complete
	attc_001	1992	2063	attC	attC	complete
	DACTYR010000057.1_4	2058	2687	protein	AAC_6p_Ib-NCBIFAM	complete
	P_intI1	2800	2834	Promoter	Pint_1	complete
	DACTYR010000057.1_5	2855	3607	protein	intI	complete
	DACTYR010000121.1_1	1	756	protein	intI	complete
	DACTYR010000121.1_2	924	1553	protein	AAC_6p_Ib-NCBIFAM	complete
	attc_001	1548	1619	attC	attC	complete
	DACTYR010000121.1_3	1722	2069	protein	SMR_qac_E-NCBIFAM	complete
888	DACTYT010000051.1_1	3	122	protein	protein	complete
	attc_001	238	297	attC	attC	complete
	DACTYT010000051.1_2	299	1174	protein	ANT_3pp_AadA1-NCBIFAM	complete
	DACTYT010000051.1_3	1226	1651	protein	protein	complete
	DACTYT010000051.1_4	1656	2237	protein	AAC_6p_Ia_fam-NCBIFAM	complete
	P_intI1	2362	2396	Promoter	Pint_1	complete
	DACTYT010000051.1_5	2417	3208	protein	intI	complete
	DACTYT010000089.1_2	785	1576	protein	intI	complete
	P_intI1	1596	1630	Promoter	Pint_1	complete
	attI1	1655	1713	attI	attI_1	complete
	DACTYT010000089.1_3	1756	2337	protein	AAC_6p_Ia_fam-NCBIFAM	complete
	DACTYT010000089.1_4	2342	2767	protein	protein	complete
	DACTYT010000089.1_5	2819	3694	protein	ANT_3pp_AadA1-NCBIFAM	complete

	attc_001	3696	3755	attC	attC	complete
895	DACYPP010000045.1_1	3	122	protein	protein	complete
	attc_001	238	297	attC	attC	complete
	DACYPP010000045.1_2	299	1174	protein	ANT_3pp_AadA1-NCBIFAM	complete
	DACYPP010000045.1_3	1226	1651	protein	protein	complete
	DACYPP010000045.1_4	1656	2213	protein	AAC_6p_Ia_fam-NCBIFAM	complete
	P_intI1	2338	2372	Promoter	Pint_1	complete
	DACYPP010000045.1_5	2393	3184	protein	intI	complete
	DACYPP010000083.1_2	825	1616	protein	intI	complete
	P_intI1	1636	1670	Promoter	Pint_1	complete
	attI1	1695	1753	attI	attI_1	complete
	DACYPP010000083.1_3	1796	2353	protein	AAC_6p_Ia_fam-NCBIFAM	complete
	DACYPP010000083.1_4	2358	2783	protein	protein	complete
	DACYPP010000083.1_5	2835	3710	protein	ANT_3pp_AadA1-NCBIFAM	complete
	attc_001	3712	3771	attC	attC	complete
928	NZ_RAQW01000005.1_144	142685	143698	protein	intI	complete
	P_intI1	143718	143752	Promoter	Pint_1	complete
	NZ_RAQW01000005.1_145	143799	144428	protein	AAC_6p_Ib-NCBIFAM	complete
	attc_001	144423	144494	attC	attC	complete
	NZ_RAQW01000005.1_146	144510	145286	protein	ANT_3pp_AadA1-NCBIFAM	complete
	attc_002	145288	145347	attC	attC	complete
	NZ_RAQW01000005.1_147	145450	145797	protein	SMR_qac_E-NCBIFAM	complete
1372	DACTWZ010000049.1_1	3	122	protein	protein	complete
	attc_001	238	297	attC	attC	complete
	DACTWZ010000049.1_2	299	1174	protein	ANT_3pp_AadA1-NCBIFAM	complete
	DACTWZ010000049.1_3	1226	1651	protein	protein	complete
	DACTWZ010000049.1_4	1656	2213	protein	AAC_6p_Ia_fam-NCBIFAM	complete
	P_intI1	2338	2372	Promoter	Pint_1	complete
	DACTWZ010000049.1_5	2393	3184	protein	intI	complete
	DACTWZ010000092.1_2	785	1576	protein	intI	complete
	P_intI1	1596	1630	Promoter	Pint_1	complete
	attI1	1655	1713	attI	attI_1	complete
	DACTWZ010000092.1_3	1756	2313	protein	AAC_6p_Ia_fam-NCBIFAM	complete
	DACTWZ010000092.1_4	2318	2743	protein	protein	complete
	DACTWZ010000092.1_5	2795	3670	protein	ANT_3pp_AadA1-NCBIFAM	complete
	attc_001	3672	3731	attC	attC	complete
1373	DACTXA010000059.1_3	1542	1889	protein	SMR_qac_E-NCBIFAM	complete
	attc_001	1992	2063	attC	attC	complete
	DACTXA010000059.1_4	2058	2687	protein	AAC_6p_Ib-NCBIFAM	complete
	P_intI1	2800	2834	Promoter	Pint_1	complete
	DACTXA010000059.1_5	2855	3607	protein	intI	complete

	DACTXA010000124.1_1	1	756	protein	intI	complete
	DACTXA010000124.1_2	924	1553	protein	AAC_6p_Ib-NCBIFAM	complete
	attc_001	1548	1619	attC	attC	complete
	DACTXA010000124.1_3	1722	2069	protein	SMR_qac_E-NCBIFAM	complete
1374	DACTXC010000055.1_3	1542	1889	protein	SMR_qac_E-NCBIFAM	complete
	attc_001	1992	2063	attC	attC	complete
	DACTXC010000055.1_4	2058	2687	protein	AAC_6p_Ib-NCBIFAM	complete
	attI1	2717	2775	attI	attI_1	complete
	P_intI1	2800	2834	Promoter	Pint_1	complete
	DACTXC010000055.1_5	2855	3646	protein	intI	complete
	DACTXC010000128.1_2	785	1576	protein	intI	complete
	P_intI1	1596	1630	Promoter	Pint_1	complete
	attI1	1655	1713	attI	attI_1	complete
	DACTXC010000128.1_3	1744	2373	protein	AAC_6p_Ib-NCBIFAM	complete
	attc_001	2368	2439	attC	attC	complete
	DACTXC010000128.1_4	2542	2889	protein	SMR_qac_E-NCBIFAM	complete
1375	DACTXG010000051.1_1	3	122	protein	protein	complete
	attc_001	238	297	attC	attC	complete
	DACTXG010000051.1_2	299	1174	protein	ANT_3pp_AadA1-NCBIFAM	complete
	DACTXG010000051.1_3	1226	1651	protein	protein	complete
	DACTXG010000051.1_4	1656	2213	protein	AAC_6p_Ia_fam-NCBIFAM	complete
	P_intI1	2338	2372	Promoter	Pint_1	complete
	DACTXG010000051.1_5	2393	3184	protein	intI	complete
	DACTXG010000090.1_2	785	1576	protein	intI	complete
	P_intI1	1596	1630	Promoter	Pint_1	complete
	attI1	1655	1713	attI	attI_1	complete
	DACTXG010000090.1_3	1756	2313	protein	AAC_6p_Ia_fam-NCBIFAM	complete
	DACTXG010000090.1_4	2318	2743	protein	protein	complete
	DACTXG010000090.1_5	2795	3670	protein	ANT_3pp_AadA1-NCBIFAM	complete
	attc_001	3672	3731	attC	attC	complete
1378	DACTXD010000047.1_1	3	122	protein	protein	complete
	attc_001	238	297	attC	attC	complete
	DACTXD010000047.1_2	299	1174	protein	ANT_3pp_AadA1-NCBIFAM	complete
	DACTXD010000047.1_3	1226	1651	protein	protein	complete
	DACTXD010000047.1_4	1656	2213	protein	AAC_6p_Ia_fam-NCBIFAM	complete
	P_intI1	2338	2372	Promoter	Pint_1	complete
	DACTXD010000047.1_5	2393	3184	protein	intI	complete
	DACTXD010000084.1_2	785	1576	protein	intI	complete
	P_intI1	1596	1630	Promoter	Pint_1	complete
	attI1	1655	1713	attI	attI_1	complete
	DACTXD010000084.1_3	1756	2313	protein	AAC_6p_Ia_fam-NCBIFAM	complete

	DACTXD010000084.1_4	2318	2743	protein	protein	complete
	DACTXD010000084.1_5	2795	3670	protein	ANT_3pp_AadA1-NCBIFAM	complete
	attc_001	3672	3731	attC	attC	complete
1379	DACTXI010000052.1_1	3	122	protein	protein	complete
	attc_001	238	297	attC	attC	complete
	DACTXI010000052.1_2	299	1174	protein	ANT_3pp_AadA1-NCBIFAM	complete
	DACTXI010000052.1_3	1226	1651	protein	protein	complete
	DACTXI010000052.1_4	1656	2213	protein	AAC_6p_Ia_fam-NCBIFAM	complete
	P_intI1	2338	2372	Promoter	Pint_1	complete
	DACTXI010000052.1_5	2393	3184	protein	intI	complete
	DACTXI010000085.1_2	785	1576	protein	intI	complete
	P_intI1	1596	1630	Promoter	Pint_1	complete
	attI1	1655	1713	attI	attI_1	complete
	DACTXI010000085.1_3	1756	2313	protein	AAC_6p_Ia_fam-NCBIFAM	complete
	DACTXI010000085.1_4	2318	2743	protein	protein	complete
	DACTXI010000085.1_5	2795	3670	protein	ANT_3pp_AadA1-NCBIFAM	complete
	attc_001	3672	3731	attC	attC	complete
1381	DACSAR010000051.1_1	3	122	protein	protein	complete
	attc_001	238	297	attC	attC	complete
	DACSAR010000051.1_2	299	1174	protein	ANT_3pp_AadA1-NCBIFAM	complete
	DACSAR010000051.1_3	1226	1651	protein	protein	complete
	DACSAR010000051.1_4	1656	2213	protein	AAC_6p_Ia_fam-NCBIFAM	complete
	P_intI1	2338	2372	Promoter	Pint_1	complete
	DACSAR010000051.1_5	2393	3184	protein	intI	complete
	DACSAR010000084.1_2	825	1616	protein	intI	complete
	P_intI1	1636	1670	Promoter	Pint_1	complete
	attI1	1695	1753	attI	attI_1	complete
	DACSAR010000084.1_3	1796	2353	protein	AAC_6p_Ia_fam-NCBIFAM	complete
	DACSAR010000084.1_4	2358	2783	protein	protein	complete
	attc_001	2798	2844	attC	attC	complete
1382	attc_001	46	105	attC	attC	complete
	DACSBA010000055.1_1	107	982	protein	ANT_3pp_AadA1-NCBIFAM	complete
	DACSBA010000055.1_2	1034	1459	protein	protein	complete
	DACSBA010000055.1_3	1464	2021	protein	AAC_6p_Ia_fam-NCBIFAM	complete
	P_intI1	2146	2180	Promoter	Pint_1	complete
	DACSBA010000055.1_4	2201	2992	protein	intI	complete
	DACSBA010000095.1_2	825	1616	protein	intI	complete
	P_intI1	1636	1670	Promoter	Pint_1	complete
	attI1	1695	1753	attI	attI_1	complete
	DACSBA010000095.1_3	1796	2353	protein	AAC_6p_Ia_fam-NCBIFAM	complete
	DACSBA010000095.1_4	2358	2783	protein	protein	complete

	attc_001	2798	2844	attC	attC	complete
1383	DACSAV010000062.1_1	3	122	protein	protein	complete
	attc_001	238	297	attC	attC	complete
	DACSAV010000062.1_2	299	1174	protein	ANT_3pp_AadA1-NCBIFAM	complete
	DACSAV010000062.1_3	1226	1651	protein	protein	complete
	DACSAV010000062.1_4	1656	2213	protein	AAC_6p_Ia_fam-NCBIFAM	complete
	P_intI1	2338	2372	Promoter	Pint_1	complete
	DACSAV010000062.1_5	2393	3184	protein	intI	complete
	DACSAV010000105.1_2	825	1616	protein	intI	complete
	P_intI1	1636	1670	Promoter	Pint_1	complete
	attI1	1695	1753	attI	attI_1	complete
	DACSAV010000105.1_3	1796	2353	protein	AAC_6p_Ia_fam-NCBIFAM	complete
	DACSAV010000105.1_4	2358	2783	protein	protein	complete
	DACSAV010000105.1_5	2835	3710	protein	ANT_3pp_AadA1-NCBIFAM	complete
	attc_001	3712	3771	attC	attC	complete
1386	DACSAT010000052.1_3	1542	1889	protein	SMR_qac_E-NCBIFAM	complete
	attc_001	1992	2063	attC	attC	complete
	DACSAT010000052.1_4	2058	2687	protein	AAC_6p_Ib-NCBIFAM	complete
	P_intI1	2800	2834	Promoter	Pint_1	complete
	DACSAT010000052.1_5	2855	3607	protein	intI	complete
	DACSAT010000111.1_1	5	796	protein	intI	complete
	DACSAT010000111.1_2	964	1593	protein	AAC_6p_Ib-NCBIFAM	complete
	attc_001	1588	1652	attC	attC	complete
1387	DACSBB010000054.1_1	3	122	protein	protein	complete
	attc_001	238	297	attC	attC	complete
	DACSBB010000054.1_2	299	1174	protein	ANT_3pp_AadA1-NCBIFAM	complete
	DACSBB010000054.1_3	1226	1651	protein	protein	complete
	DACSBB010000054.1_4	1656	2213	protein	AAC_6p_Ia_fam-NCBIFAM	complete
	P_intI1	2338	2372	Promoter	Pint_1	complete
	DACSBB010000054.1_5	2393	3184	protein	intI	complete
	DACSBB010000096.1_2	825	1616	protein	intI	complete
	P_intI1	1636	1670	Promoter	Pint_1	complete
	attI1	1695	1753	attI	attI_1	complete
	DACSBB010000096.1_3	1796	2353	protein	AAC_6p_Ia_fam-NCBIFAM	complete
	DACSBB010000096.1_4	2358	2783	protein	protein	complete
	DACSBB010000096.1_5	2835	3710	protein	ANT_3pp_AadA1-NCBIFAM	complete
	attc_001	3712	3771	attC	attC	complete
	DACSBB010000097.1_2	825	1616	protein	intI	complete
	P_intI1	1636	1670	Promoter	Pint_1	complete
	attI1	1695	1753	attI	attI_1	complete
	DACSBB010000097.1_3	1796	2353	protein	AAC_6p_Ia_fam-NCBIFAM	complete

	DACSBB010000097.1_4	2358	2783	protein	protein	complete
	DACSBB010000097.1_5	2835	3710	protein	ANT_3pp_AadA1-NCBIFAM	complete
	attc_001	3712	3771	attC	attC	complete
1388	DACSBC010000051.1_1	3	122	protein	protein	complete
	attc_001	238	297	attC	attC	complete
	DACSBC010000051.1_2	299	1174	protein	ANT_3pp_AadA1-NCBIFAM	complete
	DACSBC010000051.1_3	1226	1651	protein	protein	complete
	DACSBC010000051.1_4	1656	2213	protein	AAC_6p_Ia_fam-NCBIFAM	complete
	P_intI1	2338	2372	Promoter	Pint_1	complete
	DACSBC010000051.1_5	2393	3184	protein	intI	complete
	DACSBC010000089.1_2	825	1616	protein	intI	complete
	P_intI1	1636	1670	Promoter	Pint_1	complete
	attI1	1695	1753	attI	attI_1	complete
	DACSBC010000089.1_3	1796	2353	protein	AAC_6p_Ia_fam-NCBIFAM	complete
	DACSBC010000089.1_4	2358	2783	protein	protein	complete
	DACSBC010000089.1_5	2835	3710	protein	ANT_3pp_AadA1-NCBIFAM	complete
	attc_001	3712	3771	attC	attC	complete
1389	DACSAU010000050.1_1	3	122	protein	protein	complete
	attc_001	238	297	attC	attC	complete
	DACSAU010000050.1_2	299	1174	protein	ANT_3pp_AadA1-NCBIFAM	complete
	DACSAU010000050.1_3	1226	1651	protein	protein	complete
	DACSAU010000050.1_4	1656	2213	protein	AAC_6p_Ia_fam-NCBIFAM	complete
	P_intI1	2338	2372	Promoter	Pint_1	complete
	DACSAU010000050.1_5	2393	3184	protein	intI	complete
	DACSAU010000088.1_2	825	1616	protein	intI	complete
	P_intI1	1636	1670	Promoter	Pint_1	complete
	attI1	1695	1753	attI	attI_1	complete
	DACSAU010000088.1_3	1796	2353	protein	AAC_6p_Ia_fam-NCBIFAM	complete
	DACSAU010000088.1_4	2358	2783	protein	protein	complete
	DACSAU010000088.1_5	2835	3710	protein	ANT_3pp_AadA1-NCBIFAM	complete
	attc_001	3712	3771	attC	attC	complete
1390	attc_001	46	105	attC	attC	complete
	DACSBN010000049.1_1	107	982	protein	ANT_3pp_AadA1-NCBIFAM	complete
	DACSBN010000049.1_2	1034	1459	protein	protein	complete
	DACSBN010000049.1_3	1464	2021	protein	AAC_6p_Ia_fam-NCBIFAM	complete
	P_intI1	2146	2180	Promoter	Pint_1	complete
	DACSBN010000049.1_4	2201	2992	protein	intI	complete
	DACSBN010000092.1_2	825	1616	protein	intI	complete
	P_intI1	1636	1670	Promoter	Pint_1	complete
	attI1	1695	1753	attI	attI_1	complete
	DACSBN010000092.1_3	1796	2353	protein	AAC_6p_Ia_fam-NCBIFAM	complete

	DACSBN010000092.1_4	2358	2783	protein	protein	complete
	DACSBN010000092.1_5	2835	3710	protein	ANT_3pp_AadA1-NCBIFAM	complete
	attc_001	3712	3771	attC	attC	complete
	DACSBN010000093.1_2	825	1616	protein	intI	complete
	P_intI1	1636	1670	Promoter	Pint_1	complete
	attI1	1695	1753	attI	attI_1	complete
	DACSBN010000093.1_3	1796	2353	protein	AAC_6p_Ia_fam-NCBIFAM	complete
	DACSBN010000093.1_4	2358	2783	protein	protein	complete
	DACSBN010000093.1_5	2835	3710	protein	ANT_3pp_AadA1-NCBIFAM	complete
	attc_001	3712	3771	attC	attC	complete
1391	DACSBE010000047.1_1	3	122	protein	protein	complete
	attc_001	238	297	attC	attC	complete
	DACSBE010000047.1_2	299	1174	protein	ANT_3pp_AadA1-NCBIFAM	complete
	DACSBE010000047.1_3	1226	1651	protein	protein	complete
	DACSBE010000047.1_4	1656	2213	protein	AAC_6p_Ia_fam-NCBIFAM	complete
	P_intI1	2338	2372	Promoter	Pint_1	complete
	DACSBE010000047.1_5	2393	3184	protein	intI	complete
	DACSBE010000081.1_2	825	1616	protein	intI	complete
	P_intI1	1636	1670	Promoter	Pint_1	complete
	attI1	1695	1753	attI	attI_1	complete
	DACSBE010000081.1_3	1796	2353	protein	AAC_6p_Ia_fam-NCBIFAM	complete
	DACSBE010000081.1_4	2358	2783	protein	protein	complete
	DACSBE010000081.1_5	2835	3710	protein	ANT_3pp_AadA1-NCBIFAM	complete
	attc_001	3712	3771	attC	attC	complete
1392	DACSBQ010000050.1_1	3	224	protein	protein	complete
	attc_001	340	399	attC	attC	complete
	DACSBQ010000050.1_2	401	1276	protein	ANT_3pp_AadA1-NCBIFAM	complete
	DACSBQ010000050.1_3	1328	1753	protein	protein	complete
	DACSBQ010000050.1_4	1758	2315	protein	AAC_6p_Ia_fam-NCBIFAM	complete
	P_intI1	2440	2474	Promoter	Pint_1	complete
	DACSBQ010000050.1_5	2495	3286	protein	intI	complete
	DACSBQ010000088.1_2	825	1616	protein	intI	complete
	P_intI1	1636	1670	Promoter	Pint_1	complete
	attI1	1695	1753	attI	attI_1	complete
	DACSBQ010000088.1_3	1796	2353	protein	AAC_6p_Ia_fam-NCBIFAM	complete
	DACSBQ010000088.1_4	2358	2783	protein	protein	complete
	DACSBQ010000088.1_5	2835	3710	protein	ANT_3pp_AadA1-NCBIFAM	complete
	attc_001	3712	3771	attC	attC	complete
1393	DACSBD010000059.1_3	1542	1889	protein	SMR_qac_E-NCBIFAM	complete
	attc_001	1992	2063	attC	attC	complete
	DACSBD010000059.1_4	2058	2687	protein	AAC_6p_Ib-NCBIFAM	complete

	attI1	2717	2775	attI	attI_1	complete
	P_intI1	2800	2834	Promoter	Pint_1	complete
	DACSBD010000059.1_5	2855	3646	protein	intI	complete
	DACSBD010000122.1_2	825	1616	protein	intI	complete
	P_intI1	1636	1670	Promoter	Pint_1	complete
	attI1	1695	1753	attI	attI_1	complete
	DACSBD010000122.1_3	1784	2413	protein	AAC_6p_Ib-NCBIFAM	complete
	attc_001	2408	2479	attC	attC	complete
	DACSBD010000122.1_4	2582	2929	protein	SMR_qac_E-NCBIFAM	complete
1394	DACSBG010000049.1_1	3	122	protein	protein	complete
	attc_001	238	297	attC	attC	complete
	DACSBG010000049.1_2	299	1174	protein	ANT_3pp_AadA1-NCBIFAM	complete
	DACSBG010000049.1_3	1226	1651	protein	protein	complete
	DACSBG010000049.1_4	1656	2213	protein	AAC_6p_Ia_fam-NCBIFAM	complete
	P_intI1	2338	2372	Promoter	Pint_1	complete
	DACSBG010000049.1_5	2393	3184	protein	intI	complete
	DACSBG010000090.1_2	825	1616	protein	intI	complete
	P_intI1	1636	1670	Promoter	Pint_1	complete
	attI1	1695	1753	attI	attI_1	complete
	DACSBG010000090.1_3	1796	2353	protein	AAC_6p_Ia_fam-NCBIFAM	complete
	DACSBG010000090.1_4	2358	2783	protein	protein	complete
	DACSBG010000090.1_5	2835	3710	protein	ANT_3pp_AadA1-NCBIFAM	complete
	attc_001	3712	3771	attC	attC	complete
1396	DACSBS010000053.1_1	3	122	protein	protein	complete
	attc_001	238	297	attC	attC	complete
	DACSBS010000053.1_2	299	1174	protein	ANT_3pp_AadA1-NCBIFAM	complete
	DACSBS010000053.1_3	1226	1651	protein	protein	complete
	DACSBS010000053.1_4	1656	2213	protein	AAC_6p_Ia_fam-NCBIFAM	complete
	P_intI1	2338	2372	Promoter	Pint_1	complete
	DACSBS010000053.1_5	2393	3184	protein	intI	complete
	DACSBS010000099.1_2	825	1616	protein	intI	complete
	P_intI1	1636	1670	Promoter	Pint_1	complete
	attI1	1695	1753	attI	attI_1	complete
	DACSBS010000099.1_3	1796	2353	protein	AAC_6p_Ia_fam-NCBIFAM	complete
	DACSBS010000099.1_4	2358	2783	protein	protein	complete
	DACSBS010000099.1_5	2835	3710	protein	ANT_3pp_AadA1-NCBIFAM	complete
	attc_001	3712	3771	attC	attC	complete
1397	DACSBW010000056.1_3	1542	1889	protein	SMR_qac_E-NCBIFAM	complete
	attc_001	1992	2063	attC	attC	complete
	DACSBW010000056.1_4	2058	2687	protein	AAC_6p_Ib-NCBIFAM	complete
	P_intI1	2800	2834	Promoter	Pint_1	complete

	DACSBW010000056.1_5	2855	3607	protein	intI	complete
	DACSBW010000114.1_1	5	796	protein	intI	complete
	DACSBW010000114.1_2	964	1593	protein	AAC_6p_Ib-NCBIFAM	complete
	attc_001	1588	1659	attC	attC	complete
	DACSBW010000114.1_3	1762	2109	protein	SMR_qac_E-NCBIFAM	complete
1398	attc_001	46	105	attC	attC	complete
	DACSBU010000050.1_1	107	982	protein	ANT_3pp_AadA1-NCBIFAM	complete
	DACSBU010000050.1_2	1034	1459	protein	protein	complete
	DACSBU010000050.1_3	1464	2021	protein	AAC_6p_Ia_fam-NCBIFAM	complete
	P_intI1	2146	2180	Promoter	Pint_1	complete
	DACSBU010000050.1_4	2201	2992	protein	intI	complete
	DACSBU010000088.1_2	825	1616	protein	intI	complete
	P_intI1	1636	1670	Promoter	Pint_1	complete
	attI1	1695	1753	attI	attI_1	complete
	DACSBU010000088.1_3	1796	2353	protein	AAC_6p_Ia_fam-NCBIFAM	complete
	DACSBU010000088.1_4	2358	2783	protein	protein	complete
	DACSBU010000088.1_5	2835	3710	protein	ANT_3pp_AadA1-NCBIFAM	complete
	attc_001	3712	3771	attC	attC	complete
1400	DACSBT010000062.1_1	3	122	protein	protein	complete
	attc_001	238	297	attC	attC	complete
	DACSBT010000062.1_2	299	1174	protein	ANT_3pp_AadA1-NCBIFAM	complete
	DACSBT010000062.1_3	1226	1651	protein	protein	complete
	DACSBT010000062.1_4	1656	2213	protein	AAC_6p_Ia_fam-NCBIFAM	complete
	P_intI1	2338	2372	Promoter	Pint_1	complete
	DACSBT010000062.1_5	2393	3184	protein	intI	complete
	DACSBT010000105.1_2	825	1616	protein	intI	complete
	P_intI1	1636	1670	Promoter	Pint_1	complete
	attI1	1695	1753	attI	attI_1	complete
	DACSBT010000105.1_3	1796	2353	protein	AAC_6p_Ia_fam-NCBIFAM	complete
	DACSBT010000105.1_4	2358	2783	protein	protein	complete
	attc_001	2798	2844	attC	attC	complete
1401	DACSBY010000060.1_3	1542	1889	protein	SMR_qac_E-NCBIFAM	complete
	attc_001	1992	2063	attC	attC	complete
	DACSBY010000060.1_4	2058	2687	protein	AAC_6p_Ib-NCBIFAM	complete
	attI1	2717	2775	attI	attI_1	complete
	P_intI1	2800	2834	Promoter	Pint_1	complete
	DACSBY010000060.1_5	2855	3646	protein	intI	complete
	DACSBY010000131.1_2	825	1616	protein	intI	complete
	P_intI1	1636	1670	Promoter	Pint_1	complete
	attI1	1695	1753	attI	attI_1	complete
	DACSBY010000131.1_3	1784	2413	protein	AAC_6p_Ib-NCBIFAM	complete

	attc_001	2408	2479	attC	attC	complete
	DACSBY010000131.1_4	2582	2929	protein	SMR_qac_E-NCBIFAM	complete
1402	DACSBX010000054.1_1	3	122	protein	protein	complete
	attc_001	238	297	attC	attC	complete
	DACSBX010000054.1_2	299	1174	protein	ANT_3pp_AadA1-NCBIFAM	complete
	DACSBX010000054.1_3	1226	1651	protein	protein	complete
	DACSBX010000054.1_4	1656	2213	protein	AAC_6p_Ia_fam-NCBIFAM	complete
	P_intI1	2338	2372	Promoter	Pint_1	complete
	DACSBX010000054.1_5	2393	3184	protein	intI	complete
	DACSBX010000088.1_2	825	1616	protein	intI	complete
	P_intI1	1636	1670	Promoter	Pint_1	complete
	attI1	1695	1753	attI	attI_1	complete
	DACSBX010000088.1_3	1796	2353	protein	AAC_6p_Ia_fam-NCBIFAM	complete
	DACSBX010000088.1_4	2358	2783	protein	protein	complete
	DACSBX010000088.1_5	2835	3710	protein	ANT_3pp_AadA1-NCBIFAM	complete
	attc_001	3712	3771	attC	attC	complete
1403	DACSCB010000049.1_1	3	122	protein	protein	complete
	attc_001	238	297	attC	attC	complete
	DACSCB010000049.1_2	299	1174	protein	ANT_3pp_AadA1-NCBIFAM	complete
	DACSCB010000049.1_3	1226	1651	protein	protein	complete
	DACSCB010000049.1_4	1656	2213	protein	AAC_6p_Ia_fam-NCBIFAM	complete
	P_intI1	2338	2372	Promoter	Pint_1	complete
	DACSCB010000049.1_5	2393	3184	protein	intI	complete
	DACSCB010000086.1_2	825	1616	protein	intI	complete
	P_intI1	1636	1670	Promoter	Pint_1	complete
	attI1	1695	1753	attI	attI_1	complete
	DACSCB010000086.1_3	1796	2353	protein	AAC_6p_Ia_fam-NCBIFAM	complete
	DACSCB010000086.1_4	2358	2783	protein	protein	complete
	DACSCB010000086.1_5	2835	3710	protein	ANT_3pp_AadA1-NCBIFAM	complete
	attc_001	3712	3771	attC	attC	complete
1404	DACSBZ010000053.1_1	3	122	protein	protein	complete
	attc_001	238	297	attC	attC	complete
	DACSBZ010000053.1_2	299	1174	protein	ANT_3pp_AadA1-NCBIFAM	complete
	DACSBZ010000053.1_3	1226	1651	protein	protein	complete
	DACSBZ010000053.1_4	1656	2213	protein	AAC_6p_Ia_fam-NCBIFAM	complete
	P_intI1	2338	2372	Promoter	Pint_1	complete
	DACSBZ010000053.1_5	2393	3184	protein	intI	complete
	DACSBZ010000093.1_2	825	1616	protein	intI	complete
	P_intI1	1636	1670	Promoter	Pint_1	complete
	attI1	1695	1753	attI	attI_1	complete
	DACSBZ010000093.1_3	1796	2353	protein	AAC_6p_Ia_fam-NCBIFAM	complete

	DACSBZ010000093.1_4	2358	2783	protein	protein	complete
	attc_001	2798	2844	attC	attC	complete
1405	DACSCA010000056.1_3	1542	1889	protein	SMR_qac_E-NCBIFAM	complete
	attc_001	1992	2063	attC	attC	complete
	DACSCA010000056.1_4	2058	2687	protein	AAC_6p_Ib-NCBIFAM	complete
	attI1	2717	2775	attI	attI_1	complete
	P_intI1	2800	2834	Promoter	Pint_1	complete
	DACSCA010000056.1_5	2855	3646	protein	intI	complete
	DACSCA010000121.1_2	825	1616	protein	intI	complete
	P_intI1	1636	1670	Promoter	Pint_1	complete
	attI1	1695	1753	attI	attI_1	complete
	DACSCA010000121.1_3	1784	2413	protein	AAC_6p_Ib-NCBIFAM	complete
	attc_001	2408	2479	attC	attC	complete
	DACSCA010000121.1_4	2582	2929	protein	SMR_qac_E-NCBIFAM	complete
1406	DACSCF010000049.1_1	3	122	protein	protein	complete
	attc_001	238	297	attC	attC	complete
	DACSCF010000049.1_2	299	1174	protein	ANT_3pp_AadA1-NCBIFAM	complete
	DACSCF010000049.1_3	1226	1651	protein	protein	complete
	DACSCF010000049.1_4	1656	2213	protein	AAC_6p_Ia_fam-NCBIFAM	complete
	P_intI1	2338	2372	Promoter	Pint_1	complete
	DACSCF010000049.1_5	2393	3184	protein	intI	complete
	DACSCF010000087.1_2	825	1616	protein	intI	complete
	P_intI1	1636	1670	Promoter	Pint_1	complete
	attI1	1695	1753	attI	attI_1	complete
	DACSCF010000087.1_3	1796	2353	protein	AAC_6p_Ia_fam-NCBIFAM	complete
	DACSCF010000087.1_4	2358	2783	protein	protein	complete
	attc_001	2798	2844	attC	attC	complete
1407	DACSCE010000063.1_3	1542	1889	protein	SMR_qac_E-NCBIFAM	complete
	attc_001	1992	2063	attC	attC	complete
	DACSCE010000063.1_4	2058	2687	protein	AAC_6p_Ib-NCBIFAM	complete
	P_intI1	2800	2834	Promoter	Pint_1	complete
	DACSCE010000063.1_5	2855	3607	protein	intI	complete
	DACSCE010000130.1_1	5	796	protein	intI	complete
	DACSCE010000130.1_2	964	1593	protein	AAC_6p_Ib-NCBIFAM	complete
	attc_001	1588	1659	attC	attC	complete
	DACSCE010000130.1_3	1762	2109	protein	SMR_qac_E-NCBIFAM	complete
1408	DACSCD010000056.1_3	1542	1889	protein	SMR_qac_E-NCBIFAM	complete
	attc_001	1992	2063	attC	attC	complete
	DACSCD010000056.1_4	2058	2687	protein	AAC_6p_Ib-NCBIFAM	complete
	attI1	2717	2775	attI	attI_1	complete
	P_intI1	2800	2834	Promoter	Pint_1	complete

	DACSCD010000056.1_5	2855	3646	protein	intI	complete
	DACSCD010000143.1_2	825	1616	protein	intI	complete
	P_intI1	1636	1670	Promoter	Pint_1	complete
	attI1	1695	1753	attI	attI_1	complete
	DACSCD010000143.1_3	1784	2413	protein	AAC_6p_Ib-NCBIFAM	complete
	attc_001	2408	2472	attC	attC	complete
1411	DACSCX010000058.1_3	1542	1889	protein	SMR_qac_E-NCBIFAM	complete
	attc_001	1992	2063	attC	attC	complete
	DACSCX010000058.1_4	2058	2687	protein	AAC_6p_Ib-NCBIFAM	complete
	P_intI1	2800	2834	Promoter	Pint_1	complete
	DACSCX010000058.1_5	2855	3607	protein	intI	complete
	DACSCX010000118.1_1	5	796	protein	intI	complete
	DACSCX010000118.1_2	964	1593	protein	AAC_6p_Ib-NCBIFAM	complete
	attc_001	1588	1659	attC	attC	complete
	DACSCX010000118.1_3	1762	2109	protein	SMR_qac_E-NCBIFAM	complete
1412	DACSCG010000047.1_1	3	122	protein	protein	complete
	attc_001	238	297	attC	attC	complete
	DACSCG010000047.1_2	299	1174	protein	ANT_3pp_AadA1-NCBIFAM	complete
	DACSCG010000047.1_3	1226	1651	protein	protein	complete
	DACSCG010000047.1_4	1656	2213	protein	AAC_6p_Ia_fam-NCBIFAM	complete
	P_intI1	2338	2372	Promoter	Pint_1	complete
	DACSCG010000047.1_5	2393	3184	protein	intI	complete
	DACSCG010000080.1_2	825	1616	protein	intI	complete
	P_intI1	1636	1670	Promoter	Pint_1	complete
	attI1	1695	1753	attI	attI_1	complete
	DACSCG010000080.1_3	1796	2353	protein	AAC_6p_Ia_fam-NCBIFAM	complete
	DACSCG010000080.1_4	2358	2783	protein	protein	complete
	DACSCG010000080.1_5	2835	3710	protein	ANT_3pp_AadA1-NCBIFAM	complete
	attc_001	3712	3771	attC	attC	complete
1414	DACSCI010000049.1_1	3	122	protein	protein	complete
	attc_001	238	297	attC	attC	complete
	DACSCI010000049.1_2	299	1174	protein	ANT_3pp_AadA1-NCBIFAM	complete
	DACSCI010000049.1_3	1226	1651	protein	protein	complete
	DACSCI010000049.1_4	1656	2213	protein	AAC_6p_Ia_fam-NCBIFAM	complete
	P_intI1	2338	2372	Promoter	Pint_1	complete
	DACSCI010000049.1_5	2393	3184	protein	intI	complete
	DACSCI010000087.1_2	825	1616	protein	intI	complete
	P_intI1	1636	1670	Promoter	Pint_1	complete
	attI1	1695	1753	attI	attI_1	complete
	DACSCI010000087.1_3	1796	2353	protein	AAC_6p_Ia_fam-NCBIFAM	complete
	DACSCI010000087.1_4	2358	2783	protein	protein	complete

	DACSCI010000087.1_5	2835	3710	protein	ANT_3pp_AadA1-NCBIFAM	complete
	attc_001	3712	3771	attC	attC	complete
1415	DACSCK010000044.1_5	2464	2811	protein	SMR_qac_E-NCBIFAM	complete
	attc_001	2914	2985	attC	attC	complete
	DACSCK010000044.1_6	2980	3609	protein	AAC_6p_Ib-NCBIFAM	complete
	attI1	3639	3697	attI	attI_1	complete
	Pc_int1	3748	3774	Promoter	Pc_1	complete
	DACSCK010000044.1_7	3778	4659	protein	intI	complete
	DACSCK010000080.1_4	1589	2470	protein	intI	complete
	Pc_int1	2473	2499	Promoter	Pc_1	complete
	attI1	2550	2608	attI	attI_1	complete
	DACSCK010000080.1_5	2639	3268	protein	AAC_6p_Ib-NCBIFAM	complete
	attc_001	3263	3334	attC	attC	complete
	DACSCK010000080.1_6	3437	3784	protein	SMR_qac_E-NCBIFAM	complete
1416	DACSCJ010000051.1_1	3	122	protein	protein	complete
	attc_001	238	297	attC	attC	complete
	DACSCJ010000051.1_2	299	1174	protein	ANT_3pp_AadA1-NCBIFAM	complete
	DACSCJ010000051.1_3	1226	1651	protein	protein	complete
	DACSCJ010000051.1_4	1656	2213	protein	AAC_6p_Ia_fam-NCBIFAM	complete
	P_intI1	2338	2372	Promoter	Pint_1	complete
	DACSCJ010000051.1_5	2393	3184	protein	intI	complete
	DACSCJ010000086.1_2	825	1616	protein	intI	complete
	P_intI1	1636	1670	Promoter	Pint_1	complete
	attI1	1695	1753	attI	attI_1	complete
	DACSCJ010000086.1_3	1796	2353	protein	AAC_6p_Ia_fam-NCBIFAM	complete
	DACSCJ010000086.1_4	2358	2783	protein	protein	complete
	DACSCJ010000086.1_5	2835	3710	protein	ANT_3pp_AadA1-NCBIFAM	complete
	attc_001	3712	3771	attC	attC	complete
1417	DACSCM010000059.1_3	1542	1889	protein	SMR_qac_E-NCBIFAM	complete
	attc_001	1992	2063	attC	attC	complete
	DACSCM010000059.1_4	2058	2687	protein	AAC_6p_Ib-NCBIFAM	complete
	P_intI1	2800	2834	Promoter	Pint_1	complete
	DACSCM010000059.1_5	2855	3607	protein	intI	complete
	DACSCM010000123.1_1	5	796	protein	intI	complete
	DACSCM010000123.1_2	964	1593	protein	AAC_6p_Ib-NCBIFAM	complete
	attc_001	1588	1659	attC	attC	complete
	DACSCM010000123.1_3	1762	2109	protein	SMR_qac_E-NCBIFAM	complete
1719	DACSCL010000050.1_1	3	122	protein	protein	complete
	attc_001	238	297	attC	attC	complete
	DACSCL010000050.1_2	299	1174	protein	ANT_3pp_AadA1-NCBIFAM	complete
	DACSCL010000050.1_3	1226	1651	protein	protein	complete

	DACSCL010000050.1_4	1656	2213	protein	AAC_6p_Ia_fam-NCBIFAM	complete
	P_intI1	2338	2372	Promoter	Pint_1	complete
	DACSCL010000050.1_5	2393	3184	protein	intI	complete
	DACSCL010000086.1_2	825	1616	protein	intI	complete
	P_intI1	1636	1670	Promoter	Pint_1	complete
	attI1	1695	1753	attI	attI_1	complete
	DACSCL010000086.1_3	1796	2353	protein	AAC_6p_Ia_fam-NCBIFAM	complete
	DACSCL010000086.1_4	2358	2783	protein	protein	complete
	DACSCL010000086.1_5	2835	3710	protein	ANT_3pp_AadA1-NCBIFAM	complete
	attc_001	3712	3771	attC	attC	complete
1420	DACSCN010000050.1_1	3	122	protein	protein	complete
	attc_001	238	297	attC	attC	complete
	DACSCN010000050.1_2	299	1174	protein	ANT_3pp_AadA1-NCBIFAM	complete
	DACSCN010000050.1_3	1226	1651	protein	protein	complete
	DACSCN010000050.1_4	1656	2213	protein	AAC_6p_Ia_fam-NCBIFAM	complete
	P_intI1	2338	2372	Promoter	Pint_1	complete
	DACSCN010000050.1_5	2393	3184	protein	intI	complete
	DACSCN010000091.1_2	825	1616	protein	intI	complete
	P_intI1	1636	1670	Promoter	Pint_1	complete
	attI1	1695	1753	attI	attI_1	complete
	DACSCN010000091.1_3	1796	2353	protein	AAC_6p_Ia_fam-NCBIFAM	complete
	DACSCN010000091.1_4	2358	2783	protein	protein	complete
	DACSCN010000091.1_5	2835	3710	protein	ANT_3pp_AadA1-NCBIFAM	complete
	attc_001	3712	3771	attC	attC	complete
1424	DACSCS010000059.1_3	1542	1889	protein	SMR_qac_E-NCBIFAM	complete
	attc_001	1992	2063	attC	attC	complete
	DACSCS010000059.1_4	2058	2687	protein	AAC_6p_Ib-NCBIFAM	complete
	P_intI1	2800	2834	Promoter	Pint_1	complete
	DACSCS010000059.1_5	2855	3607	protein	intI	complete
	DACSCS010000126.1_1	5	796	protein	intI	complete
	DACSCS010000126.1_2	964	1593	protein	AAC_6p_Ib-NCBIFAM	complete
	attc_001	1588	1659	attC	attC	complete
	DACSCS010000126.1_3	1762	2109	protein	SMR_qac_E-NCBIFAM	complete
1425	DACSCT010000061.1_3	1542	1889	protein	SMR_qac_E-NCBIFAM	complete
	attc_001	1992	2063	attC	attC	complete
	DACSCT010000061.1_4	2058	2687	protein	AAC_6p_Ib-NCBIFAM	complete
	P_intI1	2800	2834	Promoter	Pint_1	complete
	DACSCT010000061.1_5	2855	3607	protein	intI	complete
	DACSCT010000117.1_1	5	796	protein	intI	complete
	DACSCT010000117.1_2	964	1593	protein	AAC_6p_Ib-NCBIFAM	complete
	attc_001	1588	1659	attC	attC	complete

	DACSCT010000117.1_3	1762	2109	protein	SMR_qac_E-NCBIFAM	complete
1426	DACSCU010000064.1_3	1542	1889	protein	SMR_qac_E-NCBIFAM	complete
	attc_001	1992	2063	attC	attC	complete
	DACSCU010000064.1_4	2058	2687	protein	AAC_6p_Ib-NCBIFAM	complete
	P_intI1	2800	2834	Promoter	Pint_1	complete
	DACSCU010000064.1_5	2855	3607	protein	intI	complete
	DACSCU010000116.1_1	5	796	protein	intI	complete
	DACSCU010000116.1_2	964	1593	protein	AAC_6p_Ib-NCBIFAM	complete
	attc_001	1588	1659	attC	attC	complete
	DACSCU010000116.1_3	1762	2109	protein	SMR_qac_E-NCBIFAM	complete
1427	DACSDA010000064.1_3	1542	1889	protein	SMR_qac_E-NCBIFAM	complete
	attc_001	1992	2063	attC	attC	complete
	DACSDA010000064.1_4	2058	2687	protein	AAC_6p_Ib-NCBIFAM	complete
	P_intI1	2800	2834	Promoter	Pint_1	complete
	DACSDA010000064.1_5	2855	3607	protein	intI	complete
	DACSDA010000128.1_1	5	796	protein	intI	complete
	DACSDA010000128.1_2	964	1593	protein	AAC_6p_Ib-NCBIFAM	complete
	attc_001	1588	1659	attC	attC	complete
	DACSDA010000128.1_3	1762	2109	protein	SMR_qac_E-NCBIFAM	complete
1428	DACSCW010000066.1_3	1542	1889	protein	SMR_qac_E-NCBIFAM	complete
	attc_001	1992	2063	attC	attC	complete
	DACSCW010000066.1_4	2058	2687	protein	AAC_6p_Ib-NCBIFAM	complete
	P_intI1	2800	2834	Promoter	Pint_1	complete
	DACSCW010000066.1_5	2855	3607	protein	intI	complete
	DACSCW010000129.1_1	5	796	protein	intI	complete
	DACSCW010000129.1_2	964	1593	protein	AAC_6p_Ib-NCBIFAM	complete
	attc_001	1588	1659	attC	attC	complete
	DACSCW010000129.1_3	1762	2109	protein	SMR_qac_E-NCBIFAM	complete
1430	DACSCZ010000055.1_3	1542	1889	protein	SMR_qac_E-NCBIFAM	complete
	attc_001	1992	2063	attC	attC	complete
	DACSCZ010000055.1_4	2058	2687	protein	AAC_6p_Ib-NCBIFAM	complete
	P_intI1	2800	2834	Promoter	Pint_1	complete
	DACSCZ010000055.1_5	2855	3607	protein	intI	complete
	DACSCZ010000118.1_1	5	796	protein	intI	complete
	DACSCZ010000118.1_2	964	1593	protein	AAC_6p_Ib-NCBIFAM	complete
	attc_001	1588	1659	attC	attC	complete
	DACSCZ010000118.1_3	1762	2109	protein	SMR_qac_E-NCBIFAM	complete
1436	DACTVL010000057.1_3	1542	1889	protein	SMR_qac_E-NCBIFAM	complete
	attc_001	1992	2063	attC	attC	complete
	DACTVL010000057.1_4	2058	2687	protein	AAC_6p_Ib-NCBIFAM	complete
	P_intI1	2800	2834	Promoter	Pint_1	complete

	DACTVL010000057.1_5	2855	3607	protein	intI	complete
	DACTVL010000124.1_1	1	756	protein	intI	complete
	DACTVL010000124.1_2	924	1553	protein	AAC_6p_Ib-NCBIFAM	complete
	attc_001	1548	1619	attC	attC	complete
	DACTVL010000124.1_3	1722	2069	protein	SMR_qac_E-NCBIFAM	complete
1437	DACTVK010000050.1_1	3	122	protein	protein	complete
	attc_001	238	297	attC	attC	complete
	DACTVK010000050.1_2	299	1174	protein	ANT_3pp_AadA1-NCBIFAM	complete
	DACTVK010000050.1_3	1226	1651	protein	protein	complete
	DACTVK010000050.1_4	1656	2213	protein	AAC_6p_Ia_fam-NCBIFAM	complete
	P_intI1	2338	2372	Promoter	Pint_1	complete
	DACTVK010000050.1_5	2393	3184	protein	intI	complete
	DACTVK010000089.1_2	785	1576	protein	intI	complete
	P_intI1	1596	1630	Promoter	Pint_1	complete
	attI1	1655	1713	attI	attI_1	complete
	DACTVK010000089.1_3	1756	2313	protein	AAC_6p_Ia_fam-NCBIFAM	complete
	DACTVK010000089.1_4	2318	2743	protein	protein	complete
	attc_001	2758	2804	attC	attC	complete
1438	DACTVN010000056.1_3	1542	1889	protein	SMR_qac_E-NCBIFAM	complete
	attc_001	1992	2063	attC	attC	complete
	DACTVN010000056.1_4	2058	2687	protein	AAC_6p_Ib-NCBIFAM	complete
	P_intI1	2800	2834	Promoter	Pint_1	complete
	DACTVN010000056.1_5	2855	3607	protein	intI	complete
	DACTVN010000116.1_1	1	756	protein	intI	complete
	DACTVN010000116.1_2	924	1553	protein	AAC_6p_Ib-NCBIFAM	complete
	attc_001	1548	1619	attC	attC	complete
	DACTVN010000116.1_3	1722	2069	protein	SMR_qac_E-NCBIFAM	complete
1439	DACTVP010000053.1_1	3	122	protein	protein	complete
	attc_001	238	297	attC	attC	complete
	DACTVP010000053.1_2	299	1174	protein	ANT_3pp_AadA1-NCBIFAM	complete
	DACTVP010000053.1_3	1226	1651	protein	protein	complete
	DACTVP010000053.1_4	1656	2213	protein	AAC_6p_Ia_fam-NCBIFAM	complete
	P_intI1	2338	2372	Promoter	Pint_1	complete
	DACTVP010000053.1_5	2393	3184	protein	intI	complete
	DACTVP010000091.1_2	785	1576	protein	intI	complete
	P_intI1	1596	1630	Promoter	Pint_1	complete
	attI1	1655	1713	attI	attI_1	complete
	DACTVP010000091.1_3	1756	2313	protein	AAC_6p_Ia_fam-NCBIFAM	complete
	DACTVP010000091.1_4	2318	2743	protein	protein	complete
	DACTVP010000091.1_5	2795	3670	protein	ANT_3pp_AadA1-NCBIFAM	complete
	attc_001	3672	3731	attC	attC	complete

1440	DACTVF010000057.1_3	1542	1889	protein	SMR_qac_E-NCBIFAM	complete
	attc_001	1992	2063	attC	attC	complete
	DACTVF010000057.1_4	2058	2687	protein	AAC_6p_Ib-NCBIFAM	complete
	P_intI1	2800	2834	Promoter	Pint_1	complete
	DACTVF010000057.1_5	2855	3607	protein	intI	complete
	DACTVF010000123.1_1	1	756	protein	intI	complete
	DACTVF010000123.1_2	924	1553	protein	AAC_6p_Ib-NCBIFAM	complete
	attc_001	1548	1619	attC	attC	complete
	DACTVF010000123.1_3	1722	2069	protein	SMR_qac_E-NCBIFAM	complete
1441	DACTVE010000057.1_3	1542	1889	protein	SMR_qac_E-NCBIFAM	complete
	attc_001	1992	2063	attC	attC	complete
	DACTVE010000057.1_4	2058	2687	protein	AAC_6p_Ib-NCBIFAM	complete
	P_intI1	2800	2834	Promoter	Pint_1	complete
	DACTVE010000057.1_5	2855	3607	protein	intI	complete
	DACTVE010000120.1_1	1	756	protein	intI	complete
	DACTVE010000120.1_2	924	1553	protein	AAC_6p_Ib-NCBIFAM	complete
	attc_001	1548	1619	attC	attC	complete
	DACTVE010000120.1_3	1722	2069	protein	SMR_qac_E-NCBIFAM	complete
1442	DACTVO010000059.1_3	1542	1889	protein	SMR_qac_E-NCBIFAM	complete
	attc_001	1992	2063	attC	attC	complete
	DACTVO010000059.1_4	2058	2687	protein	AAC_6p_Ib-NCBIFAM	complete
	attI1	2717	2775	attI	attI_1	complete
	P_intI1	2800	2834	Promoter	Pint_1	complete
	DACTVO010000059.1_5	2855	3646	protein	intI	complete
	DACTVO010000136.1_2	785	1576	protein	intI	complete
	P_intI1	1596	1630	Promoter	Pint_1	complete
	attI1	1655	1713	attI	attI_1	complete
	DACTVO010000136.1_3	1744	2373	protein	AAC_6p_Ib-NCBIFAM	complete
	attc_001	2368	2439	attC	attC	complete
	DACTVO010000136.1_4	2542	2889	protein	SMR_qac_E-NCBIFAM	complete
1443	DACTUZ010000057.1_3	1542	1889	protein	SMR_qac_E-NCBIFAM	complete
	attc_001	1992	2063	attC	attC	complete
	DACTUZ010000057.1_4	2058	2687	protein	AAC_6p_Ib-NCBIFAM	complete
	P_intI1	2800	2834	Promoter	Pint_1	complete
	DACTUZ010000057.1_5	2855	3607	protein	intI	complete
	DACTUZ010000119.1_1	1	756	protein	intI	complete
	DACTUZ010000119.1_2	924	1553	protein	AAC_6p_Ib-NCBIFAM	complete
	attc_001	1548	1619	attC	attC	complete
	DACTUZ010000119.1_3	1722	2069	protein	SMR_qac_E-NCBIFAM	complete
1444	DACTVH010000050.1_1	3	122	protein	protein	complete
	attc_001	238	297	attC	attC	complete

	DACTVH010000050.1_2	299	1174	protein	ANT_3pp_AadA1-NCBIFAM	complete
	DACTVH010000050.1_3	1226	1651	protein	protein	complete
	DACTVH010000050.1_4	1656	2213	protein	AAC_6p_Ia_fam-NCBIFAM	complete
	P_intI1	2338	2372	Promoter	Pint_1	complete
	DACTVH010000050.1_5	2393	3184	protein	intI	complete
	DACTVH010000078.1_2	785	1576	protein	intI	complete
	P_intI1	1596	1630	Promoter	Pint_1	complete
	attI1	1655	1713	attI	attI_1	complete
	DACTVH010000078.1_3	1756	2313	protein	AAC_6p_Ia_fam-NCBIFAM	complete
	DACTVH010000078.1_4	2318	2743	protein	protein	complete
	DACTVH010000078.1_5	2795	3670	protein	ANT_3pp_AadA1-NCBIFAM	complete
	attc_001	3672	3731	attC	attC	complete
1445	DACTVI010000057.1_3	1542	1889	protein	SMR_qac_E-NCBIFAM	complete
	attc_001	1992	2063	attC	attC	complete
	DACTVI010000057.1_4	2058	2687	protein	AAC_6p_Ib-NCBIFAM	complete
	P_intI1	2800	2834	Promoter	Pint_1	complete
	DACTVI010000057.1_5	2855	3607	protein	intI	complete
	DACTVI010000117.1_1	1	756	protein	intI	complete
	DACTVI010000117.1_2	924	1553	protein	AAC_6p_Ib-NCBIFAM	complete
	attc_001	1548	1619	attC	attC	complete
	DACTVI010000117.1_3	1722	2069	protein	SMR_qac_E-NCBIFAM	complete
1447	DACTWT010000057.1_1	3	122	protein	protein	complete
	attc_001	238	297	attC	attC	complete
	DACTWT010000057.1_2	299	1174	protein	ANT_3pp_AadA1-NCBIFAM	complete
	DACTWT010000057.1_3	1226	1651	protein	protein	complete
	DACTWT010000057.1_4	1656	2213	protein	AAC_6p_Ia_fam-NCBIFAM	complete
	P_intI1	2338	2372	Promoter	Pint_1	complete
	DACTWT010000057.1_5	2393	3184	protein	intI	complete
	DACTWT010000091.1_2	785	1576	protein	intI	complete
	P_intI1	1596	1630	Promoter	Pint_1	complete
	attI1	1655	1713	attI	attI_1	complete
	DACTWT010000091.1_3	1756	2313	protein	AAC_6p_Ia_fam-NCBIFAM	complete
	DACTWT010000091.1_4	2318	2743	protein	protein	complete
	DACTWT010000091.1_5	2795	3670	protein	ANT_3pp_AadA1-NCBIFAM	complete
	attc_001	3672	3731	attC	attC	complete
1450	DACTWW010000056.1_3	1542	1889	protein	SMR_qac_E-NCBIFAM	complete
	attc_001	1992	2063	attC	attC	complete
	DACTWW010000056.1_4	2058	2687	protein	AAC_6p_Ib-NCBIFAM	complete
	attI1	2717	2775	attI	attI_1	complete
	P_intI1	2800	2834	Promoter	Pint_1	complete
	DACTWW010000056.1_5	2855	3646	protein	intI	complete

	DACTWW010000128.1_2	785	1576	protein	intI	complete
	P_intI1	1596	1630	Promoter	Pint_1	complete
	attI1	1655	1713	attI	attI_1	complete
	DACTWW010000128.1_3	1744	2373	protein	AAC_6p_Ib-NCBIFAM	complete
	attc_001	2368	2439	attC	attC	complete
	DACTWW010000128.1_4	2542	2889	protein	SMR_qac_E-NCBIFAM	complete
1451	DACTWA010000051.1_1	3	122	protein	protein	complete
	attc_001	238	297	attC	attC	complete
	DACTWA010000051.1_2	299	1174	protein	ANT_3pp_AadA1-NCBIFAM	complete
	DACTWA010000051.1_3	1226	1651	protein	protein	complete
	DACTWA010000051.1_4	1656	2213	protein	AAC_6p_Ia_fam-NCBIFAM	complete
	P_intI1	2338	2372	Promoter	Pint_1	complete
	DACTWA010000051.1_5	2393	3184	protein	intI	complete
	DACTWA010000083.1_2	785	1576	protein	intI	complete
	P_intI1	1596	1630	Promoter	Pint_1	complete
	attI1	1655	1713	attI	attI_1	complete
	DACTWA010000083.1_3	1756	2313	protein	AAC_6p_Ia_fam-NCBIFAM	complete
	DACTWA010000083.1_4	2318	2743	protein	protein	complete
	DACTWA010000083.1_5	2795	3670	protein	ANT_3pp_AadA1-NCBIFAM	complete
	attc_001	3672	3731	attC	attC	complete
1452	DACTWU010000051.1_1	3	122	protein	protein	complete
	attc_001	238	297	attC	attC	complete
	DACTWU010000051.1_2	299	1174	protein	ANT_3pp_AadA1-NCBIFAM	complete
	DACTWU010000051.1_3	1226	1651	protein	protein	complete
	DACTWU010000051.1_4	1656	2213	protein	AAC_6p_Ia_fam-NCBIFAM	complete
	P_intI1	2338	2372	Promoter	Pint_1	complete
	DACTWU010000051.1_5	2393	3184	protein	intI	complete
	DACTWU010000086.1_2	785	1576	protein	intI	complete
	P_intI1	1596	1630	Promoter	Pint_1	complete
	attI1	1655	1713	attI	attI_1	complete
	DACTWU010000086.1_3	1756	2313	protein	AAC_6p_Ia_fam-NCBIFAM	complete
	DACTWU010000086.1_4	2318	2743	protein	protein	complete
	DACTWU010000086.1_5	2795	3670	protein	ANT_3pp_AadA1-NCBIFAM	complete
	attc_001	3672	3731	attC	attC	complete
1453	DACTWB010000056.1_3	1542	1889	protein	SMR_qac_E-NCBIFAM	complete
	attc_001	1992	2063	attC	attC	complete
	DACTWB010000056.1_4	2058	2687	protein	AAC_6p_Ib-NCBIFAM	complete
	P_intI1	2800	2834	Promoter	Pint_1	complete
	DACTWB010000056.1_5	2855	3607	protein	intI	complete
	DACTWB010000114.1_1	1	756	protein	intI	complete
	DACTWB010000114.1_2	924	1553	protein	AAC_6p_Ib-NCBIFAM	complete

	attc_001	1548	1619	attC	attC	complete
	DACTWB010000114.1_3	1722	2069	protein	SMR_qac_E-NCBIFAM	complete
1454	DACTWE010000049.1_1	3	122	protein	protein	complete
	attc_001	238	297	attC	attC	complete
	DACTWE010000049.1_2	299	1174	protein	ANT_3pp_AadA1-NCBIFAM	complete
	DACTWE010000049.1_3	1226	1651	protein	protein	complete
	DACTWE010000049.1_4	1656	2213	protein	AAC_6p_Ia_fam-NCBIFAM	complete
	P_intI1	2338	2372	Promoter	Pint_1	complete
	DACTWE010000049.1_5	2393	3184	protein	intI	complete
	DACTWE010000086.1_2	785	1576	protein	intI	complete
	P_intI1	1596	1630	Promoter	Pint_1	complete
	attI1	1655	1713	attI	attI_1	complete
	DACTWE010000086.1_3	1756	2313	protein	AAC_6p_Ia_fam-NCBIFAM	complete
	DACTWE010000086.1_4	2318	2743	protein	protein	complete
	DACTWE010000086.1_5	2795	3670	protein	ANT_3pp_AadA1-NCBIFAM	complete
	attc_001	3672	3731	attC	attC	complete
1455	DACTWC010000052.1_1	3	122	protein	protein	complete
	attc_001	238	297	attC	attC	complete
	DACTWC010000052.1_2	299	1174	protein	ANT_3pp_AadA1-NCBIFAM	complete
	DACTWC010000052.1_3	1226	1651	protein	protein	complete
	DACTWC010000052.1_4	1656	2213	protein	AAC_6p_Ia_fam-NCBIFAM	complete
	P_intI1	2338	2372	Promoter	Pint_1	complete
	DACTWC010000052.1_5	2393	3184	protein	intI	complete
	DACTWC010000083.1_2	785	1576	protein	intI	complete
	P_intI1	1596	1630	Promoter	Pint_1	complete
	attI1	1655	1713	attI	attI_1	complete
	DACTWC010000083.1_3	1756	2313	protein	AAC_6p_Ia_fam-NCBIFAM	complete
	DACTWC010000083.1_4	2318	2743	protein	protein	complete
	DACTWC010000083.1_5	2795	3670	protein	ANT_3pp_AadA1-NCBIFAM	complete
	attc_001	3672	3731	attC	attC	complete
1456	DACTWX010000047.1_1	3	122	protein	protein	complete
	attc_001	238	297	attC	attC	complete
	DACTWX010000047.1_2	299	1174	protein	ANT_3pp_AadA1-NCBIFAM	complete
	DACTWX010000047.1_3	1226	1651	protein	protein	complete
	DACTWX010000047.1_4	1656	2213	protein	AAC_6p_Ia_fam-NCBIFAM	complete
	P_intI1	2338	2372	Promoter	Pint_1	complete
	DACTWX010000047.1_5	2393	3184	protein	intI	complete
	DACTWX010000087.1_2	785	1576	protein	intI	complete
	P_intI1	1596	1630	Promoter	Pint_1	complete
	attI1	1655	1713	attI	attI_1	complete
	DACTWX010000087.1_3	1756	2313	protein	AAC_6p_Ia_fam-NCBIFAM	complete

	DACTWX010000087.1_4	2318	2743	protein	protein	complete
	DACTWX010000087.1_5	2795	3670	protein	ANT_3pp_AadA1-NCBIFAM	complete
	attc_001	3672	3731	attC	attC	complete
1457	DACTWK010000046.1_1	3	122	protein	protein	complete
	attc_001	238	297	attC	attC	complete
	DACTWK010000046.1_2	299	1174	protein	ANT_3pp_AadA1-NCBIFAM	complete
	DACTWK010000046.1_3	1226	1651	protein	protein	complete
	DACTWK010000046.1_4	1656	2213	protein	AAC_6p_Ia_fam-NCBIFAM	complete
	P_intI1	2338	2372	Promoter	Pint_1	complete
	DACTWK010000046.1_5	2393	3184	protein	intI	complete
	DACTWK010000077.1_2	785	1576	protein	intI	complete
	P_intI1	1596	1630	Promoter	Pint_1	complete
	attI1	1655	1713	attI	attI_1	complete
	DACTWK010000077.1_3	1756	2313	protein	AAC_6p_Ia_fam-NCBIFAM	complete
	DACTWK010000077.1_4	2318	2743	protein	protein	complete
	DACTWK010000077.1_5	2795	3670	protein	ANT_3pp_AadA1-NCBIFAM	complete
	attc_001	3672	3731	attC	attC	complete
1459	DACTWL010000050.1_1	3	122	protein	protein	complete
	attc_001	238	297	attC	attC	complete
	DACTWL010000050.1_2	299	1174	protein	ANT_3pp_AadA1-NCBIFAM	complete
	DACTWL010000050.1_3	1226	1651	protein	protein	complete
	DACTWL010000050.1_4	1656	2213	protein	AAC_6p_Ia_fam-NCBIFAM	complete
	P_intI1	2338	2372	Promoter	Pint_1	complete
	DACTWL010000050.1_5	2393	3184	protein	intI	complete
	DACTWL010000084.1_2	785	1576	protein	intI	complete
	P_intI1	1596	1630	Promoter	Pint_1	complete
	attI1	1655	1713	attI	attI_1	complete
	DACTWL010000084.1_3	1756	2313	protein	AAC_6p_Ia_fam-NCBIFAM	complete
	DACTWL010000084.1_4	2318	2743	protein	protein	complete
	DACTWL010000084.1_5	2795	3670	protein	ANT_3pp_AadA1-NCBIFAM	complete
	attc_001	3672	3731	attC	attC	complete
1460	DACTWG010000051.1_1	3	122	protein	protein	complete
	attc_001	238	297	attC	attC	complete
	DACTWG010000051.1_2	299	1174	protein	ANT_3pp_AadA1-NCBIFAM	complete
	DACTWG010000051.1_3	1226	1651	protein	protein	complete
	DACTWG010000051.1_4	1656	2213	protein	AAC_6p_Ia_fam-NCBIFAM	complete
	P_intI1	2338	2372	Promoter	Pint_1	complete
	DACTWG010000051.1_5	2393	3184	protein	intI	complete
	DACTWG010000078.1_2	785	1576	protein	intI	complete
	P_intI1	1596	1630	Promoter	Pint_1	complete
	attI1	1655	1713	attI	attI_1	complete

	DACTWG010000078.1_3	1756	2313	protein	AAC_6p_Ia_fam-NCBIFAM	complete
	DACTWG010000078.1_4	2318	2743	protein	protein	complete
	DACTWG010000078.1_5	2795	3670	protein	ANT_3pp_AadA1-NCBIFAM	complete
	attc_001	3672	3731	attC	attC	complete
1462	DACTWP010000051.1_1	3	122	protein	protein	complete
	attc_001	238	297	attC	attC	complete
	DACTWP010000051.1_2	299	1174	protein	ANT_3pp_AadA1-NCBIFAM	complete
	DACTWP010000051.1_3	1226	1651	protein	protein	complete
	DACTWP010000051.1_4	1656	2213	protein	AAC_6p_Ia_fam-NCBIFAM	complete
	P_intI1	2338	2372	Promoter	Pint_1	complete
	DACTWP010000051.1_5	2393	3184	protein	intI	complete
	DACTWP010000088.1_2	785	1576	protein	intI	complete
	P_intI1	1596	1630	Promoter	Pint_1	complete
	attI1	1655	1713	attI	attI_1	complete
	DACTWP010000088.1_3	1756	2313	protein	AAC_6p_Ia_fam-NCBIFAM	complete
	DACTWP010000088.1_4	2318	2743	protein	protein	complete
	DACTWP010000088.1_5	2795	3670	protein	ANT_3pp_AadA1-NCBIFAM	complete
	attc_001	3672	3731	attC	attC	complete
1463	DACTWO010000047.1_1	3	122	protein	protein	complete
	attc_001	238	297	attC	attC	complete
	DACTWO010000047.1_2	299	1174	protein	ANT_3pp_AadA1-NCBIFAM	complete
	DACTWO010000047.1_3	1226	1651	protein	protein	complete
	DACTWO010000047.1_4	1656	2213	protein	AAC_6p_Ia_fam-NCBIFAM	complete
	P_intI1	2338	2372	Promoter	Pint_1	complete
	DACTWO010000047.1_5	2393	3184	protein	intI	complete
	DACTWO010000075.1_2	785	1576	protein	intI	complete
	P_intI1	1596	1630	Promoter	Pint_1	complete
	attI1	1655	1713	attI	attI_1	complete
	DACTWO010000075.1_3	1756	2313	protein	AAC_6p_Ia_fam-NCBIFAM	complete
	DACTWO010000075.1_4	2318	2743	protein	protein	complete
	DACTWO010000075.1_5	2795	3670	protein	ANT_3pp_AadA1-NCBIFAM	complete
	attc_001	3672	3731	attC	attC	complete
1465	DACTWR010000047.1_1	3	122	protein	protein	complete
	attc_001	238	297	attC	attC	complete
	DACTWR010000047.1_2	299	1174	protein	ANT_3pp_AadA1-NCBIFAM	complete
	DACTWR010000047.1_3	1226	1651	protein	protein	complete
	DACTWR010000047.1_4	1656	2213	protein	AAC_6p_Ia_fam-NCBIFAM	complete
	P_intI1	2338	2372	Promoter	Pint_1	complete
	DACTWR010000047.1_5	2393	3184	protein	intI	complete
	DACTWR010000076.1_2	785	1576	protein	intI	complete
	P_intI1	1596	1630	Promoter	Pint_1	complete

	attI1	1655	1713	attI	attI_1	complete
	DACTWR010000076.1_3	1756	2313	protein	AAC_6p_Ia_fam-NCBIFAM	complete
	DACTWR010000076.1_4	2318	2743	protein	protein	complete
	DACTWR010000076.1_5	2795	3670	protein	ANT_3pp_AadA1-NCBIFAM	complete
	attc_001	3672	3731	attC	attC	complete
134	NRIP01000037.1_8	4971	5984	protein	intI	In0
	Pc_int1	5987	6013	Promoter	Pc_1	In0
	P_intI1	6005	6039	Promoter	Pint_1	In0
151	P_intI1	10524	10558	Promoter	Pint_1	In0
	NRIL01000023.1_15	10579	11592	protein	intI	In0
152	NRIN01000027.1_8	4965	5978	protein	intI	In0
	Pc_int1	5981	6007	Promoter	Pc_1	In0
	P_intI1	5999	6033	Promoter	Pint_1	In0
299	attI1	1141	1199	attI	attI_1	In0
	P_intI1	1224	1258	Promoter	Pint_1	In0
	DACRZB010000052.1_4	1279	2070	protein	intI	In0
	DACRZB010000081.1_2	825	1616	protein	intI	In0
	P_intI1	1636	1670	Promoter	Pint_1	In0
	attI1	1695	1753	attI	attI_1	In0
845	P_intI1	11774	11808	Promoter	Pint_1	In0
	NZ_CAACYF010000002.1_17	11829	12620	protein	intI	In0
905	P_intI1	11774	11808	Promoter	Pint_1	In0
	CAACYF010000002.1_17	11829	12620	protein	intI	In0
921	P_intI1	10524	10558	Promoter	Pint_1	In0
	NZ_NRIL01000023.1_15	10579	11592	protein	intI	In0
922	NZ_NRIN01000027.1_8	4965	5978	protein	intI	In0
	Pc_int1	5981	6007	Promoter	Pc_1	In0
	P_intI1	5999	6033	Promoter	Pint_1	In0
924	NZ_NRIP01000037.1_8	4971	5984	protein	intI	In0
	Pc_int1	5987	6013	Promoter	Pc_1	In0
	P_intI1	6005	6039	Promoter	Pint_1	In0
